# Molecular profiling of advanced malignancies guides first-line N-of-1 treatments in the I-PREDICT treatment-naïve study

**DOI:** 10.1186/s13073-021-00969-w

**Published:** 2021-10-04

**Authors:** Jason K. Sicklick, Shumei Kato, Ryosuke Okamura, Hitendra Patel, Mina Nikanjam, Paul T. Fanta, Michael E. Hahn, Pradip De, Casey Williams, Jessica Guido, Benjamin M. Solomon, Rana R. McKay, Amy Krie, Sarah G. Boles, Jeffrey S. Ross, J. Jack Lee, Brian Leyland-Jones, Scott M. Lippman, Razelle Kurzrock

**Affiliations:** 1 Department of Surgery, Division of Surgical Oncology, UC San Diego School of Medicine, San Diego, CA USA; 2 Center for Personalized Cancer Therapy, Moores Cancer Center, UC San Diego Health, 3855 Health Sciences Drive, Mail Code 0658, La Jolla, CA 92093-0658 USA; 3 Department of Medicine, Division of Hematology Oncology, UC San Diego School of Medicine, San Diego, CA USA; 4 Department of Radiology, UC San Diego School of Medicine, San Diego, CA USA; 5 Avera Cancer Institute, Sioux Falls, SD USA; 6 Foundation Medicine, Inc., Cambridge, MA USA; 7 Departments of Pathology and Urology, SUNY Upstate Medical University, Syracuse, NY USA; 8 Department of Biostatistics, University of Texas MD Anderson Cancer Center, Houston, TX USA

**Keywords:** Precision, Personalized, Genomics, Targeted, Clinical trial, Immunotherapy

## Abstract

**Background:**

Malignancies are molecularly complex and become more resistant with each line of therapy. We hypothesized that offering matched, individualized combination therapies to patients with treatment-naïve, advanced cancers would be feasible and efficacious. Patients with newly diagnosed unresectable/metastatic, poor-prognosis cancers were enrolled in a cross-institutional prospective study.

**Methods:**

A total of 145 patients were included in the study. Genomic profiling (tissue and/or circulating tumor DNA) was performed in all patients, and PD-L1 immunohistochemistry, tumor mutational burden, and microsatellite status assessment were performed in a subset of patients. We evaluated safety and outcomes: disease-control rate (stable disease for ≥ 6 months or partial or complete response), progression-free survival (PFS), and overall survival (OS).

**Results:**

Seventy-six of 145 patients (52%) were treated, most commonly for non-colorectal gastrointestinal cancers, carcinomas of unknown primary, and hepatobiliary malignancies (53% women; median age, 63 years). The median number of deleterious genomic alterations per patient was 5 (range, 0–15). Fifty-four treated patients (71%) received ≥ 1 molecularly matched therapy, demonstrating the feasibility of administering molecularly matched therapy. The Matching Score, which reflects the percentage of targeted alterations, correlated linearly with progression-free survival (*R*^2^ = 0.92; *P* = 0.01), and high (≥ 60%) Matching Score was an independent predictor of improved disease control rate [OR 3.31 (95% CI 1.01–10.83), *P* = 0.048], PFS [HR 0.55 (0.28–1.07), *P* = 0.08], and OS [HR 0.42 (0.21–0.85), *P* = 0.02]. Serious adverse event rates were similar in the unmatched and matched groups.

**Conclusions:**

Personalized combination therapies targeting a majority of a patient’s molecular alterations have antitumor activity as first-line treatment. These findings underscore the feasibility and importance of using tailored N-of-1 combination therapies early in the course of lethal malignancies.

**Trial registration:**

I-PREDICT (NCT02534675) was registered on August 25, 2015.

**Supplementary Information:**

The online version contains supplementary material available at 10.1186/s13073-021-00969-w.

## Background

Precision cancer therapy entails identifying the molecular abnormalities in a patient’s tumor(s) and fashioning a therapeutic strategy that precisely targets those aberrations [1]. Genomic sequencing of tumors has unveiled remarkably complicated molecular landscapes with most cancers possessing unique biologic heterogeneity and complexity, even if they originate from the same organ and/or share histopathologies [2–4]. To precisely target cancers with complicated molecular portfolios that differ from patient to patient, it is necessary to individualize (or personalize) treatment regimens. The most common approaches to precision cancer therapy use both single gene polymerase chain reaction (PCR)-based sequencing and next generation sequencing (NGS)-based genomic profiling of tumors to identify genomic alterations that can serve as predictive biomarkers and match each of these potential targets to corresponding therapeutic agents. Often, this approach is deployed late in a patient’s disease course when cancers are more refractory to treatment. We previously demonstrated that administering customized (N-of-1), molecularly matched combination therapies is safe and effective in heavily treated patients with advanced cancers [5]. Our new precision-personalized paradigm departs from conventional approaches to cancer therapies in that it is patient-centric rather than drug-centric. Thus, each patient receives a personalized, N-of-1 treatment plan that optimally matches agent(s) to their tumor’s biology, while also appreciating other variables (such as co-morbidities) unique to that patient.

We hypothesized that further improvement in personalized-precision outcomes could be achieved by treating patients with N-of-1 individualized, molecularly matched therapies earlier in their disease course, when less intra- and/or inter-tumoral heterogeneity may exist. Herein, we report the feasibility, safety, and efficacy results of our prospective, non-randomized, navigational trial that evaluated the use of molecular profile-based evidence to determine individualized cancer therapy for patients with treatment-naïve, advanced lethal malignancies.

## Methods

### Patients

A total of 145 patients were included in the study. The eligibility criteria were as follows: adult patients with an incurable (i.e., surgically unresectable or metastatic disease) and lethal (i.e., ≥ 50% 2-year cancer-associated mortality) cancer; patients with cancer of unknown primary or a rare tumor with no approved therapies; patients with at least one of the following: unresectable disease, metastatic disease, medically unfit for surgical resection but with an expected survival of > 3 months, disease where no conventional therapy leads to a survival benefit > 6 months, actionable alterations determined by FoundationOne; no prior systemic cancer treatment; no prior anti-tumor agents; ability to understand and the willingness to sign a written informed consent; Eastern Cooperative Oncology Group Performance Status of 0 to 1; measurable disease; New York Heart Association Functional Classification I-II; adequate organ function; able to swallow and retain oral medication; must have evaluable tissue/blood for testing; and negative serum pregnancy test and use of one form of pregnancy prevention. Exclusion criteria were as follows: two oncologists disagree on prognosis or resectability; medical disorder that would confound study analyses; and pregnant, breast-feeding, or not using pregnancy prevention. All participants provided written informed consent to the Investigation of Profile-Related Evidence Determining Individualized Cancer Therapy (I-PREDICT) study (groups 1 and 2), as well as separate written informed consent for any investigational drug trials to which they were navigated, per Internal Review Board approval guidelines. The trial opened to enrollment on February 13, 2015. The data cutoff date was November 1, 2019. All data for each patient are included in Additional file 1: Table S1.

### Study design and treatment

I-PREDICT (NCT02534675, https://clinicaltrials.gov/ct2/show/NCT02534675) is a cross-institutional, prospective navigation trial. The study design and outcomes for patients with previously treated, unresectable or metastatic cancers (group 3) have been reported [5]. Herein, we utilized the same study protocol (Additional file 2) to investigate the feasibility, efficacy, and safety of administering customized, molecularly matched combination therapies to patients with treatment-naïve unresectable (group 1) or metastatic (group 2) lethal cancers with an expected 2-year survival of less than 50%.

Genomic profiling of tumor tissues (236–405 genes) or blood-derived circulating tumor DNA (ctDNA) (62 genes) was conducted by hybrid capture-based next generation sequencing (NGS). In a subset of patients had PD-L1 immunohistochemistry (IHC) [antibody SP142 (Ventana) or 22C3 (Dako)], tumor mutational burden (TMB) and microsatellite status were also assessed, using previously described methods (*Foundation Medicine, Inc.*; CLIA-licensed and CAP-accredited laboratory; Cambridge, MA. https://www.foundationmedicine.com) [6–13]. TMB results were reported as follows: TMB-High corresponds to ≥ 20 Muts/Mb, TMB-Intermediate corresponds to 6-19 Muts/Mb, and TMB-Low corresponds to ≤ 5 Muts/Mb.

Treatment recommendations and potential overlapping drug toxicities were discussed by a molecular tumor board (MTB; either ad hoc just-in-time electronic exchange or weekly face-to-face meetings). The ad hoc just-in-time electronic MTB always included the co-authors (namely, Jason Sicklick, Shumei Kato, Ryosuke Okamura, Pradip De, Casey Williams, Brian Leyland-Jones, Razelle Kurzrock) and the treating physician. The in-person weekly MTB also included other oncologists (medical, surgical, gynecologic and radiation), pharmacologists, cancer biologists, geneticists, radiologists, pathologists, basic scientists, and bioinformaticians, as well study coordinators/navigators and medication acquisition specialists as we previously described [14–17]. These individuals varied from week to week. Unique to I-PREDICT, there was not a predesignated set of drugs and drug combinations determined by the MTB. Instead, all possible drug combinations were used, unless the MTB felt that there was reason to believe or reported/known evidence that a combination would be toxic and therefore contraindicated. The administration of customized, molecularly matched combinations (including targeted, chemotherapeutic, hormonal agents, biologic agents, and immunotherapies) was emphasized. The treating oncologist rendered the final decision regarding therapy choice. Thus, this study was uniquely patient centered since the combination of drugs was determined by the molecular alterations present in the patient’s tumor, rather than by having a limited set of preconceived options.

This study was cross-institutional [two centers: University of California San Diego (UCSD) Moores Cancer Center and Avera Cancer Institute in South Dakota] with all physicians at each site able to consent and enroll patients. Investigators updated the study information by teleconference (twice monthly) and via face-to-face meetings for UCSD study members. Data review retreats were held every 1–2 months.

### Matching score

We compared differences in outcomes according to a previously reported molecular Matching Score (MS) [5, 18–20]. In short, Matching Score is defined as the number of alterations (excluding variants of unknown significance, VUS) targeted by administered drugs divided by the total number of characterized alterations (excluding VUS) identified. We did not distinguish between potential driver versus passenger mutations. The higher the score (range, 0–100%), the “better” the match.

For instance, if a patient’s tumor harbored six characterized genomic alterations and they were given two agents that targeted three of these alterations, the score would be calculated as 50% (3 of 6). Investigators that calculated the scores (JKS, SK, RK) were blinded to patient outcomes at the time of calculations. If a patient had ≥ 2 genomic reports, the abnormalities in each test report were counted, because there can be heterogeneity between two tissue biopsies or between blood and tissue samples.

Other considerations were also relevant: (i) if a participant had two or more genomic aberrations that were in the same gene and potentially had the same signal/pathway impact, these aberrations were counted as one; (ii) two aberrations in the same gene that potentially had different oncogenic impacts or were structurally distinct (e.g., amplification and mutation) were counted as two since they have different or additive effects; (iii) if two drugs simultaneously targeted the same aberration in a well-established synergistic manner (e.g., the FDA-approved combinations of dabrafenib and trametinib for *BRAF* aberrations, or pertuzumab and trastuzumab for *ERBB2* aberrations), the impact was counted twice in both the numerator and denominator; and (iv) estrogen receptor-positive or androgen receptor-positive expression by IHC targeted by a hormone modulator (such as, letrozole) was also counted as one in both the numerator and denominator.

For small molecule inhibitors, matching was based on preclinical low inhibitory concentration 50% (IC_50_) of the drug for the target (generally less than 100 nM) or for signal transduction effectors immediately downstream of the aberrant gene product. Antibodies were considered matched if their primary target was the product of the molecular alterations. Patients whose cancers harbored a *BRCA-*related gene anomaly were considered matched if they received platinum agents or PARP inhibitors. Also, if a participant was given checkpoint blockade immunotherapy, the score was assigned as 100% for results of microsatellite instability-high (MSI-High) or high tumor mutational burden (TMB) or high positive programmed death ligand 1 (PD-L1) expression (≥ 30%) on IHC; the score was 50% for results of low positive PD-L1 on IHC or TMB-intermediate. Individuals in the immunotherapy-treated group who had, as an example, TMB-intermediate and were scored at 50%, and received matched targeted agents, had the total score calculated as 50% + (*X*% ÷2) [note: *X* = (number of alterations targeted by agents administered)/(total number of alterations)]. For example, if a malignancy had intermediate TMB and the participant was given checkpoint blockade, but also had an *PIK3CA* and *a FGFR* alteration and received an FGFR inhibitor in addition to checkpoint blockade, the score was 50% + 25% = 75%. We also considered *TP53* abnormalities as matched to drugs with anti-VEGF/VEGFR activity because several reports have demonstrated that *TP53* alterations are correlated with VEGFA up-regulation and that VEGFA inhibitory therapies associated with better treatment outcomes in patients with *TP53*-mutant cancers [21–26]. No match was scored at over 100%. More details on Matching Score calculations are in our previous report [5].

We stratified patients according to Matching Scores ≥ 60% versus 1–59% versus unmatched (0%). The matching score cut-off point of 60% was determined by using ROC-AUC of 0.733 for the Disease Control Rate (DCR = SD ≥ 6 months + PR + CR rate). We determined that the optimal DCR cutoff (for maximal rate in high matching group) was 17 of 25 pts (68%) versus 13 of 43 pts (30%) (*P* = 0.005). A second stratification of Matching Score > 50% versus ≤ 50% was also analyzed to recapitulate the assessment method used in our previously published study in patients with pretreated metastatic disease (*5*). Further gradations in Matching Score (0%, 1–39%, 40–59%, 60–99%, and 100%) were also used for some analyses. Median PFS data for the correlation analysis with these Matching Score grades were calculated by drawing the Kaplan-Meier curve.

### Primary endpoint and study objective

The primary study objective was to determine the feasibility of using molecular testing to determine therapy for patients with previously treated cancers with incurable biology (≥ 50% 2- year cancer-associated mortality). The primary endpoint was the proportion of patients who receive molecularly targeted matched treatment after recommendations based on genomic analysis.

### Secondary and exploratory endpoints

Secondary and exploratory endpoints included the following: proportion of patients with actionable genomic alterations, incidence of high-grade adverse events, disease control rate [DCR; stable disease (SD) ≥ 6 months or partial/complete response (PR/CR)], progression-free survival (PFS), and overall survival (OS). Treatment response was evaluated using Response Evaluation Criteria in Solid Tumors (RECIST), version 1.1 [27]. Tumor assessments were performed by means of computed tomography and magnetic resonance imaging, at baseline and about every eight weeks thereafter. All RECIST measurements were performed by independent central radiology review. DCRs between groups were compared using Fisher’s exact test. With the sample size of 75 evaluable patients and the accrual ratio of 1:2 (high Matching Score group [≥ 60%]: low Matching Score group [< 60%]), we would have > 80% power for detecting the estimated DCR of 65% in the high Matching Score group versus 30% in the low Matching Score with 0.05 type I error. This was calculated a priori by J.J.L. (biostatistician). The PFS and OS were calculated from the date of treatment initiation to disease progression, or any cause of death, respectively. Patients who were progression-free (for PFS) or alive (for OS) at the date of last analysis were censored at that date. The investigators calculated Matching Scores blinded to patients’ outcomes. Serious adverse events (SAEs) were graded according to the Common Terminology Criteria for Adverse Events, version 4.03 [28]. Given that toxicity and drug dosages are major concerns when administering de novo combinations, we elected to re-visit the data after the initial data cutoff of 11/01/2019. Thus, for the SAEs and dose adjustments, we re-analyzed the data thru 03/24/2021.

### Dosing drug combinations

The UCSD Moores Cancer Center and Avera Cancer Institute Data Safety Monitoring Committees monitored the safety of this study at the respective institutions. To minimize toxicities due to customized de novo combinations, dosing of each combination was discussed by the MTB as we previously reported [5]. Recommended dosing was based on the safety data gleaned from previous studies and PharmD recommendations [29–32]. For the most part, two-drug de novo combinations were started at ~ 50% of the usual dose of each drug. Three-drug de novo combinations were started at ~ 33% of the usual dose of each drug. Patients were fully informed of risks when treated with regimens lacking phase 1 data. Based upon tolerability, patients received escalating doses of drugs with regular monitoring by their treating physician. Additional file 3: Fig. S1 demonstrates the intra-patient dose adjustments, last drug dose (as a percentage of the standard drug dose for each agent), and the median percent of standard dose for each drug. No two patients had the same drug combinations and therefore, there is no toxicity correlation with any specific combination.

### Statistical analysis

All data was compiled in a Microsoft Access 2013 (version 15.0) database. In the nature of a hypothesis-driven trial, we performed sample size calculations and originally planned to enroll 75 evaluable treated patients and estimated that 40% (*N* = 30) of the patients would be assigned to the matched group (Arm A) versus 60% (*N* = 45) to the unmatched group (Arm B). However, as precision medicine has become more established, the rate of matching has increased. We ultimately enrolled 76 evaluable treated patients (of 145 treatment-naïve patients consented); 54 (71% of evaluable patients; 37% of enrolled patients) were administered > 1 drug matched to their molecular profile (Additional file 3: Fig. S2; all molecular, treatment, Matching Score, and outcomes data for each patient are included in Additional file 1: Table S1). Logistic regression was performed for binary endpoints to estimate the odds ratio (OR). Variables with *P* < 0.15 in the univariate analyses were entered into the multivariate analyses. The Kaplan–Meier method was used for PFS and OS analyses. Survival comparisons were made by the log-rank test. Cox regression models were used to estimate the hazard ratio (HR) in multivariable analysis. Statistical analyses were performed using SPSS version 24.0.

## Results

### Patients

Overall, 145 patients with treatment-naïve unresectable or metastatic cancers that had 2-year cancer-associated mortalities of ≥ 50% were enrolled. Seventy-six patients (52%) were treated and considered evaluable for analyses (nine with surgically unresectable tumors (6.2%); 67, with metastatic disease (46%). Fifty newly diagnosed cancer patients were inevaluable (untreated) and 19 newly diagnosed cancer patients were inevaluable (treated). As detailed in Additional file 1: Fig. S2, these 69 patients (48%) were inevaluable due to the following reasons: inadequate organ function, clinical deterioration, or death (*N* = 34, 23%); patient declined treatment/treatment was not started ≥ 6 months after consent (*N* = 17, 12%); lack of measurable disease (*N* = 6); lost to follow-up (*N* = 5); inadequate restaging scan (*N* = 2); and inconsistent treatment/follow-up (*N* = 2) consistent with our recent report on attrition of patients on I-PREDICT [33]. The median overall survival was 3.0 months (untreated) versus 6.9 months (treated; *P* = 0.097, data not shown). This speaks to the aggressive biology of these cancers as many of these patients did not even receive standard of care treatment as opposed to a failure of a precision medicine approach. Failure of molecular profiling (tissue and blood) affected only 3 (2%) consented patients. Thus, it was feasible to perform molecular profiling in 98% of patients.

The characteristics of the 76 treated patients are summarized in Table 1 and detailed in Additional file 3: Table S2. Their median age was 63 years (range, 23–93), 53% (*N* = 40) of the patients were women, and 19 unique cancer types were identified. The most common tumor type was non-colorectal gastrointestinal cancer [12, 16%; includes gastroesophageal adenocarcinoma (*N* = 7); adenocarcinoma of the ampulla Vater (*N* = 2); anorectal squamous cell carcinoma (*N* = 1); esophageal squamous cell carcinoma (*N* = 1); and small bowel adenocarcinoma (*N* = 1)], followed by carcinoma of unknown primary (10, 13%), non-pancreatic hepatobiliary cancer (9, 12%), and colorectal cancer (7, 9.2%). The median number of deleterious genomic alterations (excluding all variants of unknown significance) detected by NGS in tissue DNA and/or blood circulating tumor DNA (ctDNA) was 5 (range, 0–15) per patient. The median interval between study consent and treatment initiation was 0.9 months (range, 0–3.3). Most patients (62, 82%) were treated with ≥ 2 drugs (Additional file 3: Table S2 and Additional file 1: Fig. S1).

**Table 1:** Demographic and clinical characteristics of 76 treated patients

***Characteristics***	***All treated patients*** ***[N = 76]***	Matching Score			***P***-values		
		≥ 60% [***N*** = 27]	1-59% [***N*** = 27]	Unmatched [***N*** = 22]	≥ 60% vs. 1–59%	≥ 60% vs. unmatched	1–59% vs. unmatched
Age, years*							
Median (range)	63.0 (22.5-92.7)	63.5 (36.4-82.6)	62.3 (35.7-92.7)	63.4 (22.5-86.4)	0.82	0.92	0.85
≥ 63	38 (50%)	14 (51.9%)	13 (48.1%)	11 (50.0%)	> 0.99	> 0.99	> 0.99
< 63	38 (50%)	13 (48.1%)	14 (51.9%)	11 (50.0%)	--	--	--
**Gender**							
Women	40 (52.6%)	14 (51.9%)	14 (51.9%)	12 (54.5%)	> 0.99	> 0.99	> 0.99
Men	36 (47.4%)	13 (48.1%)	13 (48.1%)	10 (45.5%)	--	--	--
**Ethnicity**							
Caucasian	45 (59.2%)	15 (55.6%)	19 (70.4%)	11 (50.0%)	0.40	0.78	0.24
Hispanic	15 (19.7%)	6 (22.2%)	6 (22.2%)	3 (13.6%)	> 0.99	0.49	0.49
Asian	9 (11.8%)	4 (14.8%)	2 (7.4%)	3 (13.6%)	0.67	> 0.99	0.65
African American	3 (3.9%)	2 (7.4%)	0 (0.0%)	1 (4.5%)	0.49	> 0.99	0.45
Others	4 (5.3%)	0 (0.0%)	0 (0.0%)	4 (18.2%)	--	**0.04**	**0.04**
**Tumor type**							
Gastrointestinal, non-colorectal	12 (15.8%)	4 (14.8%)	3 (11.1%)	5 (22.7%)	> 0.99	0.71	0.44
Carcinoma of unknown primary	10 (13.2%)	6 (22.2%)	3 (11.1%)	1 (4.5%)	0.47	0.11	0.62
Hepatobiliary	9 (11.8%)	4 (14.8%)	4 (14.8%)	1 (4.5%)	> 0.99	0.36	0.36
Colorectal	7 (9.2%)	3 (11.1%)	3 (11.1%)	1 (4.5%)	> 0.99	0.62	0.62
Pancreatic	7 (9.2%)	1 (3.7%)	2 (7.4%)	4 (18.2%)	> 0.99	0.16	0.39
Head and neck	5 (6.6%)	2 (7.4%)	2 (7.4%)	1 (4.5%)	> 0.99	> 0.99	> 0.99
Appendiceal	4 (5.3%)	2 (7.4%)	0 (0.0%)	2 (9.1%)	0.49	> 0.99	0.20
Gynecologic	4 (5.3%)	0 (0.0%)	2 (7.4%)	2 (9.1%)	0.49	0.20	> 0.99
Breast	3 (3.9%)	1 (3.7%)	2 (7.4%)	0 (0.0%)	> 0.99	> 0.99	0.50
Lung, non-small cell	3 (3.9%)	2 (7.4%)	1 (3.7%)	0 (0.0%)	> 0.99	0.50	> 0.99
Genitourinary	2 (2.6%)	0 (0.0%)	0 (0.0%)	2 (9.1%)	--	0.20	0.20
Skin/melanoma	2 (2.6%)	1 (3.7%)	1 (3.7%)	0 (0.0%)	> 0.99	> 0.99	> 0.99
Others^†^	8 (10.5%)	1 (3.7%)	4 (14.8%)	3 (13.6%)	0.35	0.31	> 0.99
**Number of genomic alterations per patient**^‡^							
Median (range)	5 (0-15)	5 (1-15)	6 (0-11)	4.5 (0-11)	0.11	0.59	0.42
**Number of drugs administered**							
≥ 2 drugs	62 (81.6%)	25 (92.6%)	20 (74.1%)	17 (77.3%)	0.14	0.22	> 0.99
Single drug	14 (18.4%)	2 (7.4%)	7 (25.9%)	5 (22.7%)	--	--	--

*Age at date of treatment start

^†^Neuroendocrine tumor (*N* = 2), adrenocortical carcinoma (*N* = 1), Erdheim-Chester disease (*N* = 1), Ewing's sarcoma (*N* = 1), myxofibrosarcoma (*N* = 1), perivascular epithelioid cell neoplasm (*N* = 1), and pleomorphic sarcoma (*N* = 1)

^‡^Variants of unknown significance were excluded. Two pathogenic variants in the same gene were counted as two alterations

### Objectives

The objectives of the study included determining the feasibility of enrolling newly diagnosed patients with lethal cancers into a molecular profiling study, finding actionable alterations, and matching them to therapy. Of the 76 treated patients, 54 (71% of the treated subset or 37.2% of 145 consented subjects) were administered ≥ 1 drug matched to their molecular profile. Overall, 133 of 145 enrolled patients (91.7%) had ≥ 1 theoretically targetable alteration. Hence, the objectives of demonstrating feasibility, identifying druggable alterations, and matching tumors to treatment were met. Commonly targeted pathways included MAPK (35.2%), the cell cycle (14.8%), the ERBB family (13%), PI3K/AKT/mTOR (PI3K; 13%), and BRCA-associated DNA repair (9.3%) (Additional file 1: Fig. S1 and S3). Immune checkpoint inhibitors were administered to 26% of the matched patients (*N* = 14) based upon a ≥ 1% positive tumor cell (equals “low”) expression of PD-L1 by IHC and/or *PD-L1* (*CD274*) amplification (*N* = 11), TMB-high/intermediate (*N* = 8), and/or MSI-high (*N* = 1). There was no difference in the immunotherapy administration rate among matching groups [9 of 27 with Matching Score ≥ 60% versus 10 of 49 patients Matching Score < 60% (*P*=NS)] or matched versus unmatched patients [14 of 54 with matched versus 5 of 22 unmatched patients (*P*=NS)].

### Efficacy

In addition to feasibility, the objectives included assessing efficacy outcomes. At the data-cutoff date, there were 68 patients evaluable for DCR (i.e., 47% of the 145 enrolled patients and 89% of the 76 treated patients, with most inevaluable patients being too early for assessment (i.e., having ongoing SD for < 6 months). All 76 patients were evaluable for PFS and OS.

**Fig. 1:**
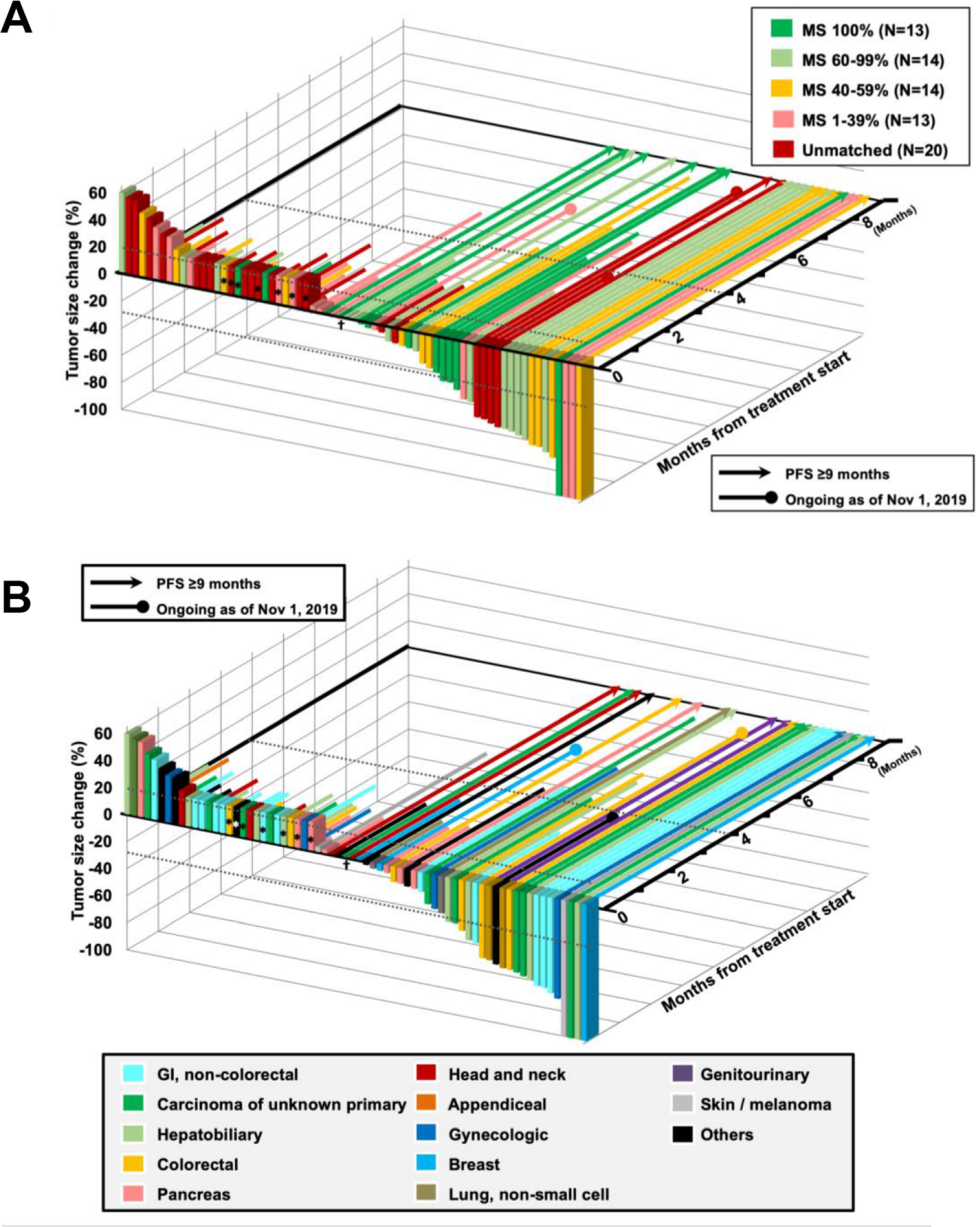
Efficacy and duration of treatment. **A** Maximum change in tumor size and duration of treatment, color coded by Matching Score. **B** Maximum change in tumor size and duration of treatment, color coded by tumor type. The 3-dimensional waterfall and swimmers plot for best response during treatment according to Matching Score (68 patients). Eight patients were excluded from this analysis, as 5 had ongoing SD for < 6 months and 3 were not yet staged at data cutoff. Asterisk symbol indicates the following: labeled as + 21% change if a patient clinically deteriorated without any restaging imaging. Dagger symbol indicates the following: one patient [ID#248] showed pseudo-progression followed by stable disease for 10.5 months. Thus, this patient was labeled as 0% change

For assessment of the Matching Score with treatment outcomes, we stratified the 76 treated patients into five categories [Matching Score: 100% (13 patients), 60–99% (14 patients), 40–59% (14 patients), 1–39% (13 patients), and 0% (unmatched, 22 patients)]. Improved DCR and PFS correlated strongly (and linearly) with the Matching Score (Pearson coefficients: for DCR, *R*^2^ 0.99, *P* < 0.001; PFS, *R*^2^ 0.96, *P* = 0.01) (Additional file 1: Fig. S4). Figure 1 demonstrates a 3-dimensional waterfall plot for the best radiological response according to Matching Score (A) and tumor type (B).

**Fig. 2:**
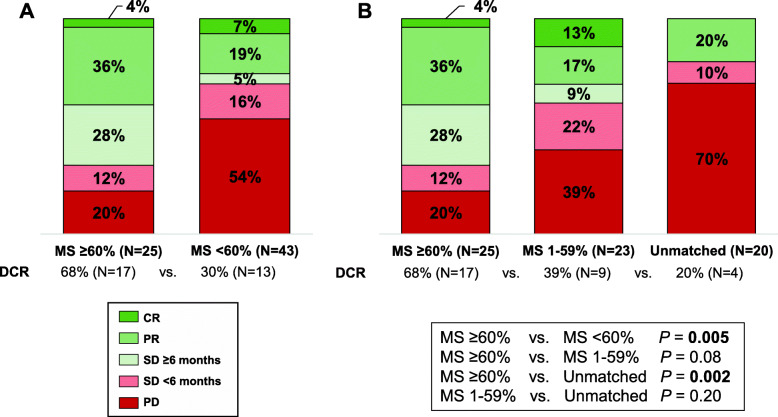
Response to therapy according to Matching Score. **A** Bar graph analyzing the percentage of patients with progressive disease (PD), stable disease (SD) < 6 months, SD ≥ 6 months, partial response (PR), and complete response (CR) for patients with a Matching Score of ≥ 60% (*N* = 25) versus < 60% (*N* = 43). *P* values were computed using a binary logistic regression test. **B** Bar graph analyzing the percentage of patients with PD, SD < 6 months, SD ≥ 6 months, PR, and CR for patients with a Matching Score of ≥ 60% (*N* = 25) versus 1–59% (*N* = 23) versus unmatched (*N* = 20). *P* values were computed as above. Sixty-eight of 76 treated patients were evaluable for response [Matching Score ≥ 60% (*N* = 25) versus < 60% (*N* = 43)]. The remaining 8 patients were excluded from this analysis as 5 had ongoing SD for < 6 months and 3 were not yet staged at data cutoff. At the data-cutoff date of November 1, 2019, the overall response rate was 14.5% for all patients. A total of 4 (2.8%) of the patients had a CR, 17 (11.7%) had a PR, 24 (16.6%) had SD (nine with SD ≥ 6 months, ten with SD < 6 months, five with on-going SD for < 6 months), 28 (19.3%) had progressive disease (PD), 3 (19.3%) were too early in treatment for response measurements, and 69 (48%) could not be evaluated, most often owing to early withdrawal for clinical deterioration. All the patients were accounted for in this analysis. CR, complete response; DCR, disease control rate (SD ≥ 6 months with PR/CR); MS, Matching Score; ORR, objective response rate (PR and CR); PD, progressive disease; PR, partial response; SD, stable disease

Overall, 27 patients (36% of 76 treated; 50% of 54 matched) had a Matching Score ≥ 60%. The remaining 49 patients had either Matching Scores of 1–59% (*N* = 27, 36% of treated) or Matching Scores of 0% (unmatched; *N* = 22, 29% of treated) (Additional file 1: Fig. S2). There were no clinically significant differences in demographic characteristics between the groups with either high (≥ 60%) or low (< 60%, including 0%) Matching Scores (Table 1). The 68% DCR in the Matching Score ≥ 60% group was significantly higher than the 30% DCR in the Matching Score < 60% group (*P* = 0.005) (Fig. 2). Multivariate analysis, adjusting for confounding factors (including cancer subtypes), demonstrated that Matching Score (≥60% versus < 60%) was the only independent predictor of higher DCR [OR (95% CI), 3.31 (1.01–10.83), *P* = 0.048] (Table 2).

**Table 2:** Multivariate analyses of disease control rate, progression-free survival, and overall survival in 76 treatment-naïve patients (*N* = 76)

	***DCR (SD ≥ 6mos/PR/CR) (N = 68***^**†**^***)***					***Progression-free Survival (N = 76)***					***Overall Survival (N = 76)***				
***Parameters***	***Univariate***			***Multivariate***		***Univariate***			***Multivariate***		***Univariate***			***Multivariate***	
	***N***	***Rate***	***P value***	***OR (95% CI)***	***P value***	***N***	***Median (months)***	***P value***	***HR (95% CI)***	***P value***	***N***	***Median (months)***	***P value***	***HR (95% CI)***	***P value***
**Age, years**															
≥63	36	44%	> 0.99	--	--	38	4.5	0.47	--	--	38	15.6	0.18	--	--
< 63	32	44%				38	4.3				38	NR			
**Gender**															
Men	29	48%	0.63	--	--	36	4.3	0.75	--	--	36	17.3	0.50	--	--
Women	39	41%				40	4.3				40	14.7			
**Treatment**															
Matched	48	54%	**0.02**	2.57 (0.65-10.21)	0.18	54	5.7	**0.007**	0.61 (0.31-1.19)	0.14	54	17.7	0.27	--	--
Unmatched	20	20%				22	2.1				22	15.6			
**Matching Score**															
≥ 60%	25	68%	**0.005**	3.31 (1.01-10.83)	**0.048**	27	11.6	**0.008**	0.55 (0.28-1.07)	0.08	27	18.7	0.053	0.42 (0.21-0.85)	**0.02**
< 60%	43	30%				49	2.8				49	11.6			
**Number of drugs**															
≥ 2 drugs	56	45%	> 0.99	--	--	62	4.3	0.88	--	--	62	14.3	0.14	2.87 (1.00-8.23)	**0.050**
Single drug	12	42%				14	5.7				14	23.0			
**GI or HPB cancer**^**‡**^															
Yes	34	38%	0.46	--	--	39	3.1	0.41	--	--	39	17.3	0.74	--	--
No	34	50%				37	5.7				37	15.6			
**CUP**															
Yes	10	60%	0.32	--	--	10	4.3	0.74	--	--	10	8.3	0.57	--	--
No	58	41%				66	4.3				66	15.6			
**Breast cancer**															
Yes	3	67%	0.58	--	--	3	NR	0.20	--	--	3	14.7	0.42	--	--
No	65	43%				73	4.3				73	16.0			

*Variables with *P* < 0.15 in the univariate were entered into the multivariate analysis

^†^Eight patients were not evaluable for the SD ≥ 6mos/PR/CR analysis because they had ongoing SD < 6 months or were too early; all patients were evaluable for PFS and OS. After data cutoff date, a re-analysis of the data showed that, included in the analysis, were two patients without target lesions by RECIST, but with evaluable disease (one with progressive disease and one with prolonged stable disease) and one patient who achieved a PR who had a baseline scan outside the four week window

^‡^Includes colorectal, GI-non-colorectal, appendiceal, hepatobiliary, and pancreatic cancers

**Fig. 3:**
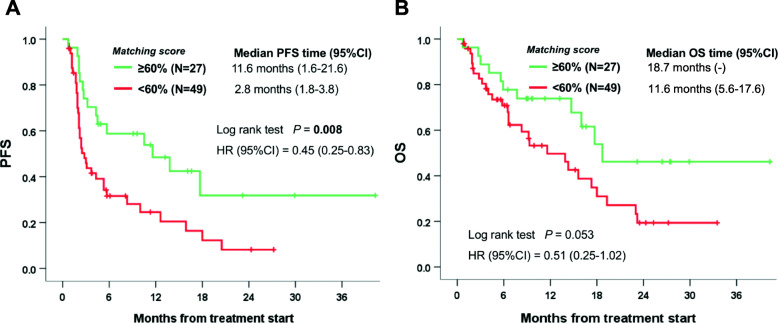
Kaplan–Meier plots of duration of progression-free survival and overall survival among all 76 treated patients by Matching Score. **A** Progression-free survival. **B** Overall survival. CI, confidence interval; HR, hazard ratio; MS, Matching Score; OS, overall survival; PFS, progression-free survival

Furthermore, the Matching Score ≥ 60% group had a significantly longer PFS versus the < 60% group (median PFS: 11.6 versus 2.8 months, *P* = 0.008) and trended toward longer OS (median OS, 18.7 months versus 11.6 months, univariate *P* = 0.053) (Fig. 3). Multivariate analyses (Table 2) indicated that Matching Score ≥ 60% was independently correlated with a longer OS [HR (95% CI), 0.42 [0.21–0.85], *P* = 0.02). Thus, our data would suggest that the Matching Score is a predictive, rather than a prognostic, marker for outcomes.

There is mounting evidence, including a recent report from the NRG Oncology cooperative group, demonstrating that mutated *TP53* predicts response to VEGF pathway inhibitors (VEGFi), including bevacizumab and pazopanib [21–23, 25, 26]. The reason for this relationship appears to be upregulation of the VEGF/VEGFR axis as one of the consequences of *TP53* mutations [21–23, 25, 26]. However, we performed a post hoc analysis to determine if eliminating patients matched on the basis of this relationship would impact the study results. In the current study, 16 patients received targeted therapies including VEGFi matched to *TP53* (Study IDs 12, 142, 148, 166, 175, 197, 198, 215, 258, 319, 322, 331, 419, 437, A005, A019). When these 16 patients were eliminated, the Matching Score ≥ 60% group had a significantly longer PFS versus the < 60% group (median PFS: 13.8 versus 2.5 months, *P* = 0.003) and significantly longer OS (median OS, Not reached versus 13.9 months, *P* = 0.02) (Additional file 1: Table S3). Moreover, the 79% DCR in the Matching Score ≥ 60% group was significantly higher than the 30% DCR in the Matching Score < 60% group (*P* = 0.004). Thus, the findings in the trial remained consistent whether the patients matched on the basis of *TP53* alterations to VEGFi were included or excluded.”

In our original I-PREDICT report [5], we described the treatment outcomes of 83 evaluable patients that were mostly heavily pretreated. To perform a more in-depth analysis of the impact of the degree of matching, we combined these 83 evaluable patients with the current 76 evaluable, treatment-naïve patients. We then analyzed the treatment outcomes of these 159 patients according to Matching Score. Again, Matching Score ≥ 60% remained an independent predictor for DCR [OR (95% CI), 2.31 (1.01–5.27), *P* = 0.047], as well as longer PFS [HR (95% CI), 0.56 (0.36–0.88), *P* = 0.01] and longer OS [HR (95% CI), 0.48 (0.28–0.82), *P* = 0.007] (Additional file 1: Table S4). Additionally, when the Matching Score was dichotomized at > 50% versus ≤ 50%, as per our prior report [5], similar results were observed (Additional file 1: Table S5-6 and Additional file 1: Fig. S5-7). In all multivariable analyses, simply classifying the patients as matched (1–100%) versus unmatched (0%) failed to discriminate the treatment outcomes very well (Table 2 and Additional file 1: Table S4-6), suggesting that the degree of matching and outcomes are consistently and positively correlated (Additional file 1: Fig. S3).

### Adverse events

The exploratory objectives included evaluation of safety. Fifty-five clinically significant serious adverse events (SAEs) at least possibly/probably related to treatment were uncommon, occurred in 18 patients (23.6% of 76 treated patients) and were divided equally among those with high or low Matching Score, as well as unmatched patients (Additional file 1: Table S7-9). There were no treatment-related deaths.

## Discussion

Precision-personalized medicine unlocks a new paradigm for addressing the cancer problem. This approach to the treatment of cancer patients began in the early 1970s with the introduction of estrogen receptor (ER) testing and reached a major milestone with the approvals of the first combination of a drug, trastuzumab and a companion diagnostic test, the HercepTest for the treatment of patients with ER-positive and HER2-positive breast cancer [34, 35]. Some of the results to date have been remarkable, including the near-normalization of life expectancy for chronic myelogenous leukemia—a previously fatal disease—when the molecular *BCR-ABL* aberration is targeted by cognate inhibitors early in the disease [36]. Examples in solid tumors include the high rate of durable responses in *ALK*-rearranged or *NTRK* fusion-bearing malignancies [37, 38]. However, optimizing precision medicine has been confounded by the complexity of metastatic cancers, which often have multiple molecular alterations that differ from patient to patient [39]. Here, we demonstrate, for the first time, that individualized, precision-matched N-of-1 therapies targeting a majority of identified molecular alterations had marked antitumor activity in patients with a spectrum of fatal, treatment-naïve cancers.

One of the reasons that we pursued a study in treatment-naïve patients was the high rate of attrition due to early death or deterioration that we observed in our prior study of heavily pretreated patients [5, 33]. Yet, even in this study, 23% of patients were not evaluable due to early clinical deterioration or death—a rate lower than the ~ 38% in our prior study [5], but still substantial. This suggests that physicians chose the present trial for patients with heavy disease burdens and/or anticipated aggressive courses, despite being newly diagnosed, consistent with our previous reports. Even so, we were able to demonstrate that matching patients to therapies was associated with improved outcomes. Importantly, analyzing matching as a dichotomous variable (matched versus unmatched), as is usually performed [40–44], did not significantly correlate with outcomes in multivariate analysis; rather, the degree of matching was important. Indeed, Matching Score (which reflects the degree of matching) correlated strongly with outcomes. Higher degrees of matching directly and linearly correlated with better DCR (*R*^2^ 0.99; *P* < 0.001) and PFS (*R*^2^ 0.92; *P* = 0.01). Moreover, higher degrees of matching were also independently associated with longer OS (*P* = 0.02) (Additional file 1: Fig. S4 and Table 2). The DCR (rate of SD ≥ 6 months with PR/CR) was 68% versus 20% for high Matching Score (MS ≥ 60%) versus unmatched patients (*P* = 0.002) (Figure 2). The need for tailored combination therapies is not surprising, as metastatic tumors have remarkably complex molecular portfolios [2–4, 39]. Of interest, the DCRs and ORRs seen herein are in the same range as those that have led to Food and Drug Administration approvals [45, 46], albeit with a smaller number of patients in our study, highlighting the need for larger trials to validate our findings. The pan-cancer setting of the current study limited our ability to assess histology-specific impact but, if confirmed in larger studies, may point to the generalizability of the results across histologies as recently demonstrated by the pan-cancer approvals of pembrolizumab for all MSI-high solid tumors [47] and the NRTK inhibitors [38] for all *NTRK* fusion cancers.

This type of trial design has several limitations. First, the study lacks a control group and therefore, there is no comparison to standard of care or other control arms that provide evidence that this approach is better than standard of care. Considering the trial design and lack of a control group, we are unable to perform an intention-to-treat (ITT) analysis, which is best reserved for randomized controlled trials of unselected patients [48]. However, it is highly unlikely that disease control and/or objective responses would occur without the impact of therapy, since these patients had, by eligibility criteria, lethal malignancies with 2-year mortality rates of at least 50%. However, other than through a randomized trial, it is impossible to rule out all confounders. In contrast, per-protocol analysis, as performed here, is best for precision medicine studies wherein biomarker-based patient selection occurs. In addition, with expansion of gene panels, as well as application of other NGS panels, the number of genomic alterations detected may also differ per assay. As a result, a specific Matching Score may also differ between panels due to differences in the denominators of genes assayed and found to be altered. Although we used a uniform genomic panel in this study, we also recognize that dichotomizing Matching Scores can be somewhat arbitrary for this reason. And, in fact, we found that Matching Score (which reflects the degree of matching) is a continuous variable that correlates linearly with outcomes as our data would suggest. As molecular profiling is increasingly done by whole-exome or genome sequencing, the Molecular Matching Score cutoffs will change, but the concept of targeting multiple oncogenic targets will remain. Another limitation relates to the fact that the current study does not account for differences in RNA expression [20], which we also know to be important in defining treatment. In addition, our findings may have physician and/or patient self-selection bias for individuals that sought out enrollment on this trial, as well as the fact that some physicians elected to enroll patients and treat them with targeted agents, rather than chemotherapy, due to concerns about the potential for rapid clinical deterioration on traditional regimens. While perhaps counterintuitive, the physician selection bias may account for the rapid clinical deterioration in many patients even though they were treated early in their disease courses (since physicians appear to have chosen patients with aggressive disease for the trial). But this may also be a study strength as it represents true real-world practice patterns. An additional limitation of the paper is that genomic-based treatment cannot be benchmarked with historical data for standard therapy alone. But we can postulate that like the previously treated patients on the study [5] that have similar diseases and demographics as the current cohort (Additional file 1: Fig. S7) would have eventually failed standard of care therapy. In summary, additional studies with larger sample sizes across multiple institutions are needed to validate our findings.

Despite these limitations, one of the important ingredients of this study is our Molecular Tumor Board (MTB) [14–17]. We recently showed that patients who receive MTB-recommended regimens (versus physician choice) have significantly longer progression-free (PFS) and overall survival (OS), as well as are better matched to therapy [16]. The current work builds upon this in the treatment naïve setting where degree of matching is an independent predictor of improved oncologic outcomes including survival. We believe that any academic center should be capable of reproducing and scaling an MTB. Furthermore, in today’s era of video teleconferences, this participation can be enhanced by increased remote participation. In fact, this in currently being done in the national TCF-001 TRACK (Target Rare Cancer Knowledge) Study (NCT04504604) of precision medicine in rare cancers. Experts from around the US converge on a weekly basis to discuss cases via video teleconference, thus leveraging this powerful new technology. But it is important to note that treating newly diagnosed, treatment-naïve cancer patients with an N-of-1 approach over standard of care requires a strong understanding of molecular results and molecularly guided therapies. For physicians starting to treat patients with customized combination therapies, we would recommend starting with previously treated patients with aggressive malignancies that have failed earlier lines of therapy before considering this approach in treatment naïve patients. With time, experience, and expertise, we believe our data suggest that it may be safe to begin moving this to the first line in select patients as we did from 2015 thru 2019. Finally, and most importantly, we believe that our study should be reported so it provides an impetus and foundation for others to duplicate it. Studies by other groups are needed to validate our findings.

## Conclusions

As we hone our understanding of tumor biology and drug targeting, properly assessing outcomes will require modernizing our methodologies for a patient-centered model that seeks out specific biomarkers of responsiveness and hence represents the tenets of precision-personalized medicine. The current study affirms the benefit of targeting multiple versus single drivers. It also shows that it is feasible to move this approach earlier in the treatment process of lethal malignancies, with the objective of improving outcomes before resistance mutations accumulate. In conclusion, this study provides the first evidence that customized combination therapies administered in an N-of-1 fashion can be given safely and effectively in the first line setting in patients with incurable, poor-prognosis malignancies.

## Supplementary Information

**Additional file 1: Table S1**. Molecular results and drug matches for treated patients only. **Table S2**. Raw data for 145 patients.

**Additional file 2:.** I-PREDICT study protocol

**Additional file 3: Table S3**. Multivariate Analyses of Progression-free Survival, Overall Survival, and Disease Control Rate in All Treatment-naïve Patients (*N* = 76) and Excluding Patients with TP53 Mutations Matched to VEGF Inhibitor (VEGFi) Therapy (*N* = 60). **Table S4**. Variables predicting outcome in I-PREDICT treatment-naïve patients (*N* = 76) combined with I-PREDICT patients with ≥1 prior line of therapy (*N* = 83) (Matching Score dichotomized at ≥60% versus < 60%). **Table S5**. Variables predicting outcomes among I-PREDICT treatment-naïve patients (*N* = 76) (Matching Score dichotomized at > 50% versus ≤50% as per prior report [5]). **Table S6**. Variables predicting outcome in I-PREDICT treatment-naïve patients (*N* = 76) combined with I-PREDICT patients with ≥1 prior line of therapy (*N* = 83) (Matching Score dichotomized at > 50% versus ≤50% as per prior report [5]). **Table S7**. Rates of serious adverse events (SAE; grades 3-5 of CTCAE v4.03) according to Matching Score in 76 treated patients. Table S8. Possibly/Probably Related serious adverse events (SAE; grades 3-5 of CTCAE v4.03, *N* = 45 events) according to Grade, Matching Score, and Relationship to Treatment. **Table S9**. All serious adverse events (SAE; grades 3-5) according to CTCAE v4.03 System Organ Class for all matched and unmatched patients. **Figure S1**. Co-drug plot of numbers of drugs per patient, drug dose adjustments per patient, and percent of standard drug doses for each drug per patient, and median drug dose per agent for matched patients according to Matching Score. **Figure S2**. CONSORT Diagram of I-PREDICT Treatment-Naïve Patients (percent of 145 patients). **Figure S3**. Percentage of Molecularly Matched Patients (*N* = 54) with a Gene or Pathway Targeted. **Figure S4**. Strong Linear Correlation between Matching Score and Outcome. **Figure S5**. Response to treatment among patients evaluable for SD ≥ 6 months/PR/CR (*N* = 68 of 76 treated patients) (Matching Score > 50% [*N* = 27] versus ≤50% [*N* = 41]). **Figure S6**. Kaplan-Meier curves for (A) progression-free survival and (B) overall survival according to Matching Score of > 50% [*N* = 30] versus ≤50% [*N* = 46]. **Figure S7**. Kaplan-Meier curves for (A) progression-free survival and (B) overall survival according to Matching Score among I-PREDICT treatment-naïve patients (*N* = 76) combined with I-PREDICT patients with ≥1 prior line of therapy (*N* = 83).

## Data Availability

All data generated can be found within the main manuscript and its additional files. The clinical trial protocol is available in Additional file 2. All raw patient-level data (excluding raw next generation sequencing data files generated by Foundation Medicine, Inc.) are available in Additional files 1 and 3, which is in compliance with our IRB approvals, patient consent, and confidentiality agreements. Raw next generation sequencing data files generated by Foundation Medicine, Inc. are not publicly available in order to maintain patient confidentiality.

## Abbreviations

ctDNA: Blood-derived circulating tumor DNA
DCR: Disease control rate
ER: Estrogen receptor
HR: Hazard ratio
IHC: Immunohistochemistry
ITT: Intention-to-treat
MS: Matching score
MSI-High: Microsatellite instability-high
MTB: Molecular tumor board
NGS: Next generation sequencing
ORR: Objective response rate
OR: Odds ratio
OS: Overall survival
PR/CR: Partial/complete response
PD-L1: Programmed death ligand 1
PFS: Progression-free survival
ROC: Receiver-operating characteristic curve
RECIST: Response evaluation criteria in solid tumors
SAEs: Serious adverse events
TMB: Tumor mutational burden
TMB: Tumor mutational burden
VUS: Variants of unknown significance
VEGFi: VEGF inhibitor

## References

[1] Subbiah V, Kurzrock R (2018). Challenging standard-of-care paradigms in the precision oncology era. Trends Cancer.

[2] Kurzrock R, Giles FJ (2015). Precision oncology for patients with advanced cancer: the challenges of malignant snowflakes. Cell Cycle.

[3] Bieg-Bourne CC, Millis SZ, Piccioni DE, Fanta PT, Goldberg ME, Chmielecki J, Parker BA, Kurzrock R (2017). Next-generation sequencing in the clinical setting clarifies patient characteristics and potential actionability. Cancer Res.

[4] Bertucci F, Ng CKY, Patsouris A, Droin N, Piscuoglio S, Carbuccia N, Soria JC, Dien AT, Adnani Y, Kamal M, Garnier S, Meurice G, Jimenez M, Dogan S, Verret B, Chaffanet M, Bachelot T, Campone M, Lefeuvre C, Bonnefoi H, Dalenc F, Jacquet A, de Filippo MR, Babbar N, Birnbaum D, Filleron T, le Tourneau C, André F (2019). Genomic characterization of metastatic breast cancers. Nature.

[5] Sicklick JK, Kato S, Okamura R, Schwaederle M, Hahn ME, Williams CB, de P, Krie A, Piccioni DE, Miller VA, Ross JS, Benson A, Webster J, Stephens PJ, Lee JJ, Fanta PT, Lippman SM, Leyland-Jones B, Kurzrock R (2019). Molecular profiling of cancer patients enables personalized combination therapy: the I-PREDICT study. Nat Med.

[6] Thomas RK, Nickerson E, Simons JF, Jänne PA, Tengs T, Yuza Y, Garraway LA, LaFramboise T, Lee JC, Shah K, O'Neill K, Sasaki H, Lindeman N, Wong KK, Borras AM, Gutmann EJ, Dragnev KH, DeBiasi R, Chen TH, Glatt KA, Greulich H, Desany B, Lubeski CK, Brockman W, Alvarez P, Hutchison SK, Leamon JH, Ronan MT, Turenchalk GS, Egholm M, Sellers WR, Rothberg JM, Meyerson M (2006). Sensitive mutation detection in heterogeneous cancer specimens by massively parallel picoliter reactor sequencing. Nat Med.

[7] Fitzgibbons PL, Bradley LA, Fatheree LA, Alsabeh R, Fulton RS, Goldsmith JD, Haas TS, Karabakhtsian RG, Loykasek PA, Marolt MJ, Shen SS, Smith AT, Swanson PE, College of American Pathologists Pathology and Laboratory Quality Center (2014). Principles of analytic validation of immunohistochemical assays: Guideline from the College of American Pathologists Pathology and Laboratory Quality Center. Arch Pathol Lab Med.

[8] Chalmers ZR, Connelly CF, Fabrizio D, Gay L, Ali SM, Ennis R, Schrock A, Campbell B, Shlien A, Chmielecki J, Huang F, He Y, Sun J, Tabori U, Kennedy M, Lieber DS, Roels S, White J, Otto GA, Ross JS, Garraway L, Miller VA, Stephens PJ, Frampton GM (2017). Analysis of 100,000 human cancer genomes reveals the landscape of tumor mutational burden. Genome Med.

[9] Goodman AM, Kato S, Bazhenova L, Patel SP, Frampton GM, Miller V, Stephens PJ, Daniels GA, Kurzrock R (2017). Tumor mutational burden as an independent predictor of response to immunotherapy in diverse cancers. Mol Cancer Ther.

[10] Hall MJ, Gowen K, Sanford EM, Elvin JA, Ali SM, Kaczmar J, White E, Malboeuf C, Ross JS, Miller VA, Stephens P, Yelensky R, Daly MB, Sun J (2016). Evaluation of microsatellite instability (MSI) status in 11,573 diverse solid tumors using comprehensive genomic profiling (CGP). Journal of Clinical Oncology.

[11] Cortes-Ciriano I, Lee S, Park W-Y, Kim T-M, Park PJ (2017). A molecular portrait of microsatellite instability across multiple cancers. Nat Commun.

[12] Clark TA, Chung JH, Kennedy M, Hughes JD, Chennagiri N, Lieber DS, Fendler B, Young L, Zhao M, Coyne M, Breese V, Young G, Donahue A, Pavlick D, Tsiros A, Brennan T, Zhong S, Mughal T, Bailey M, He J, Roels S, Frampton GM, Spoerke JM, Gendreau S, Lackner M, Schleifman E, Peters E, Ross JS, Ali SM, Miller VA, Gregg JP, Stephens PJ, Welsh A, Otto GA, Lipson D (2018). Analytical validation of a hybrid capture-based next-generation sequencing clinical assay for genomic profiling of cell-free circulating tumor DNA. J Mol Diagn.

[13] Trabucco SE, Gowen K, Maund SL, Sanford E, Fabrizio DA, Hall MJ, Yakirevich E, Gregg JP, Stephens PJ, Frampton GM, Hegde PS, Miller VA, Ross JS, Hartmaier RJ, Huang SMA, Sun JX (2019). A novel next-generation sequencing approach to detecting microsatellite instability and pan-tumor characterization of 1000 microsatellite instability-high cases in 67,000 patient Samples. J Mol Diagn.

[14] Schwaederle M, Parker BA, Schwab RB, Fanta PT, Boles SG, Daniels GA, Bazhenova LA, Subramanian R, Coutinho AC, Ojeda-Fournier H, Datnow B, Webster NJ, Lippman SM, Kurzrock R (2014). Molecular tumor board: the University of California-San Diego Moores Cancer Center experience. Oncologist.

[15] Patel M, Kato SM, Kurzrock R (2018). Molecular tumor boards: realizing precision oncology therapy. Clin Pharmacol Ther.

[16] Kato S, Kim KH, Lim HJ, Boichard A, Nikanjam M, Weihe E, Kuo DJ, Eskander RN, Goodman A, Galanina N, Fanta PT, Schwab RB, Shatsky R, Plaxe SC, Sharabi A, Stites E, Adashek JJ, Okamura R, Lee S, Lippman SM, Sicklick JK, Kurzrock R (2020). Real-world data from a molecular tumor board demonstrates improved outcomes with a precision N-of-One strategy. Nat Commun.

[17] Parker BA (2015). Breast cancer experience of the molecular tumor board at the University of California, San Diego Moores Cancer Center. J Oncol Pract.

[18] Schwaederle M, Parker BA, Schwab RB, Daniels GA, Piccioni DE, Kesari S, Helsten TL, Bazhenova LA, Romero J, Fanta PT, Lippman SM, Kurzrock R (2016). Precision Oncology: The UC San Diego Moores Cancer Center PREDICT Experience. Mol Cancer Ther.

[19] Wheler JJ, Janku F, Naing A, Li Y, Stephen B, Zinner R, Subbiah V, Fu S, Karp D, Falchook GS, Tsimberidou AM, Piha-Paul S, Anderson R, Ke D, Miller V, Yelensky R, Lee JJ, Hong DS, Kurzrock R (2016). Cancer therapy directed by comprehensive genomic profiling: a single center study. Cancer Res.

[20] Rodon J, Soria JC, Berger R, Miller WH, Rubin E, Kugel A, Tsimberidou A, Saintigny P, Ackerstein A, Braña I, Loriot Y, Afshar M, Miller V, Wunder F, Bresson C, Martini JF, Raynaud J, Mendelsohn J, Batist G, Onn A, Tabernero J, Schilsky RL, Lazar V, Lee JJ, Kurzrock R (2019). Genomic and transcriptomic profiling expands precision cancer medicine: the WINTHER trial. Nat Med.

[21] Wheler JJ, Janku F, Naing A, Li Y, Stephen B, Zinner R, Subbiah V, Fu S, Karp D, Falchook GS, Tsimberidou AM, Piha-Paul S, Anderson R, Ke D, Miller V, Yelensky R, Lee JJ, Hong D, Kurzrock R (2016). TP53 Alterations correlate with response to VEGF/VEGFR inhibitors: implications for targeted therapeutics. Mol Cancer Ther.

[22] Koehler K, Liebner D, Chen JL (2016). TP53 mutational status is predictive of pazopanib response in advanced sarcomas. Ann Oncol.

[23] Said R (2013). P53 mutations in advanced cancers: clinical characteristics, outcomes, and correlation between progression-free survival and bevacizumab-containing therapy. Oncotarget.

[24] Schwaederlé M, Lazar V, Validire P, Hansson J, Lacroix L, Soria JC, Pawitan Y, Kurzrock R (2015). VEGF-A expression correlates with TP53 mutations in non-small cell lung cancer: implications for antiangiogenesis therapy. Cancer Res.

[25] Li AM, Boichard A, Kurzrock R (2020). Mutated TP53 is a marker of increased VEGF expression: analysis of 7,525 pan-cancer tissues. Cancer Biol Ther.

[26] Leslie KK, Filiaci VL, Mallen AR, Thiel KW, Devor EJ, Moxley K, Richardson D, Mutch D, Secord AA, Tewari KS, McDonald ME, Mathews C, Cosgrove C, Dewdney S, Casablanca Y, Jackson A, Rose PG, Zhou XC, McHale M, Lankes H, Levine DA, Aghajanian C (2021). Mutated p53 portends improvement in outcomes when bevacizumab is combined with chemotherapy in advanced/recurrent endometrial cancer: an NRG Oncology study. Gynecol Oncol.

[27] Eisenhauer EA, Therasse P, Bogaerts J, Schwartz LH, Sargent D, Ford R, Dancey J, Arbuck S, Gwyther S, Mooney M, Rubinstein L, Shankar L, Dodd L, Kaplan R, Lacombe D, Verweij J (2009). New response evaluation criteria in solid tumours: revised RECIST guideline (version 1.1). Eur J Cancer.

[28] Department of Health and Human Services. Common Terminology Criteria for Adverse Events (CTCAE), v. J., 2010. <CTCAE_4.03_2010-06-14_QuickReference_8.5 × 11.pdf > (2010).

[29] Nikanjam M, Patel H, Kurzrock R (2017). Dosing immunotherapy combinations: analysis of 3,526 patients for toxicity and response patterns. Oncoimmunology.

[30] Nikanjam M, Liu S, Yang J, Kurzrock R (2017). Dosing three-drug combinations that include targeted anti-cancer agents: analysis of 37,763 patients. Oncologist.

[31] Nikanjam M, Liu S, Kurzrock R (2016). Dosing targeted and cytotoxic two-drug combinations: lessons learned from analysis of 24,326 patients reported 2010 through 2013. Int J Cancer.

[32] Liu S, Nikanjam M, Kurzrock R (2016). Dosing de novo combinations of two targeted drugs: towards a customized precision medicine approach to advanced cancers. Oncotarget.

[33] Bohan SS, Sicklick JK, Kato S, Okamura R, Miller VA, Leyland-Jones B, Lippman SM, Kurzrock R (2020). Attrition of patients on a precision oncology trial: analysis of the I-PREDICT experience. Oncologist.

[34] McGuire WL, De La Garza M, Chamness GC (1977). Evaluation of estrogen receptor assays in human breast cancer tissue. Cancer Res.

[35] Slamon DJ, Leyland-Jones B, Shak S, Fuchs H, Paton V, Bajamonde A, Fleming T, Eiermann W, Wolter J, Pegram M, Baselga J, Norton L (2001). Use of chemotherapy plus a monoclonal antibody against HER2 for metastatic breast cancer that overexpresses HER2. N Engl J Med.

[36] Bower H, Björkholm M, Dickman PW, Höglund M, Lambert PC, Andersson TML (2016). Life expectancy of patients with chronic myeloid leukemia approaches the life expectancy of the general population. J Clin Oncol.

[37] Shaw AT, Kim DW, Nakagawa K, Seto T, Crinó L, Ahn MJ, de Pas T, Besse B, Solomon BJ, Blackhall F, Wu YL, Thomas M, O'Byrne KJ, Moro-Sibilot D, Camidge DR, Mok T, Hirsh V, Riely GJ, Iyer S, Tassell V, Polli A, Wilner KD, Jänne PA (2013). Crizotinib versus chemotherapy in advanced ALK-positive lung cancer. N Engl J Med.

[38] Drilon A, Laetsch TW, Kummar S, DuBois SG, Lassen UN, Demetri GD, Nathenson M, Doebele RC, Farago AF, Pappo AS, Turpin B, Dowlati A, Brose MS, Mascarenhas L, Federman N, Berlin J, el-Deiry WS, Baik C, Deeken J, Boni V, Nagasubramanian R, Taylor M, Rudzinski ER, Meric-Bernstam F, Sohal DPS, Ma PC, Raez LE, Hechtman JF, Benayed R, Ladanyi M, Tuch BB, Ebata K, Cruickshank S, Ku NC, Cox MC, Hawkins DS, Hong DS, Hyman DM (2018). Efficacy of larotrectinib in TRK fusion-positive cancers in adults and children. N Engl J Med.

[39] Gerlinger M, Rowan AJ, Horswell S, Larkin J, Endesfelder D, Gronroos E, Martinez P, Matthews N, Stewart A, Tarpey P, Varela I, Phillimore B, Begum S, McDonald NQ, Butler A, Jones D, Raine K, Latimer C, Santos CR, Nohadani M, Eklund AC, Spencer-Dene B, Clark G, Pickering L, Stamp G, Gore M, Szallasi Z, Downward J, Futreal PA, Swanton C (2012). Intratumor heterogeneity and branched evolution revealed by multiregion sequencing. N Engl J Med.

[40] Massard C, Michiels S, Ferté C, le Deley MC, Lacroix L, Hollebecque A, Verlingue L, Ileana E, Rosellini S, Ammari S, Ngo-Camus M, Bahleda R, Gazzah A, Varga A, Postel-Vinay S, Loriot Y, Even C, Breuskin I, Auger N, Job B, de Baere T, Deschamps F, Vielh P, Scoazec JY, Lazar V, Richon C, Ribrag V, Deutsch E, Angevin E, Vassal G, Eggermont A, André F, Soria JC (2017). High-throughput genomics and clinical outcome in hard-to-treat advanced cancers: results of the MOSCATO 01 trial. Cancer Discov.

[41] Le Tourneau C (2015). Molecularly targeted therapy based on tumour molecular profiling versus conventional therapy for advanced cancer (SHIVA): a multicentre, open-label, proof-of-concept, randomised, controlled phase 2 trial. Lancet Oncol.

[42] Stockley TL, Oza AM, Berman HK, Leighl NB, Knox JJ, Shepherd FA, Chen EX, Krzyzanowska MK, Dhani N, Joshua AM, Tsao MS, Serra S, Clarke B, Roehrl MH, Zhang T, Sukhai MA, Califaretti N, Trinkaus M, Shaw P, van der Kwast T, Wang L, Virtanen C, Kim RH, Razak ARA, Hansen AR, Yu C, Pugh TJ, Kamel-Reid S, Siu LL, Bedard PL (2016). Molecular profiling of advanced solid tumors and patient outcomes with genotype-matched clinical trials: the Princess Margaret IMPACT/COMPACT trial. Genome Med.

[43] Hainsworth JD, Meric-Bernstam F, Swanton C, Hurwitz H, Spigel DR, Sweeney C, Burris HA, Bose R, Yoo B, Stein A, Beattie M, Kurzrock R (2018). Targeted therapy for advanced solid tumors on the basis of molecular profiles: results from MyPathway, an open-label, phase IIa multiple basket study. J Clin Oncol.

[44] Tredan O (2019). Molecular screening program to select molecular-based recommended therapies for metastatic cancer patients: analysis from the ProfiLER trial. Ann Oncol.

[45] Chapman PB, Hauschild A, Robert C, Haanen JB, Ascierto P, Larkin J, Dummer R, Garbe C, Testori A, Maio M, Hogg D, Lorigan P, Lebbe C, Jouary T, Schadendorf D, Ribas A, O'Day SJ, Sosman JA, Kirkwood JM, Eggermont AM, Dreno B, Nolop K, Li J, Nelson B, Hou J, Lee RJ, Flaherty KT, McArthur G, BRIM-3 Study Group (2011). Improved survival with vemurafenib in melanoma with BRAF V600E mutation. N Engl J Med.

[46] Bang YJ, van Cutsem E, Feyereislova A, Chung HC, Shen L, Sawaki A, Lordick F, Ohtsu A, Omuro Y, Satoh T, Aprile G, Kulikov E, Hill J, Lehle M, Rüschoff J, Kang YK (2010). Trastuzumab in combination with chemotherapy versus chemotherapy alone for treatment of HER2-positive advanced gastric or gastro-oesophageal junction cancer (ToGA): a phase 3, open-label, randomised controlled trial. Lancet.

[47] Le DT (2015). PD-1 Blockade in tumors with mismatch-repair deficiency. N Engl J Med.

[48] Sicklick JK, Kato S, Okamura R, Kurzrock R (2020). Precision oncology: the intention-to-treat analysis fallacy. Eur J Cancer.

