# Supplementary material for: Molecular profiling of advanced malignancies guides first-line N-of-1 treatments in the I-PREDICT treatment-naïve study
Source: Genome Med. 2021 Oct 4;13:155. doi: 10.1186/s13073-021-00969-w (PMC8491393; doi:10.1186/s13073-021-00969-w)
Supplement: Supplementary file 2 — Additional file 2:. I-PREDICT study protocol [file 13073_2021_969_MOESM2_ESM.pdf]

I-PREDICT

An open label navigational *Investigation* of molecular Profile-Related Evidence Determining Individualized Cancer Therapy for patients with incurable malignancies and poor prognoses.

LIST OF INVESTIGATORS

PRINCIPAL INVESTIGATOR
Jason Sicklick, MD
Associate Professor of Surgery
UC San Diego Moores Cancer Center
3855 Health Sciences Drive
La Jolla, CA 92093
jsicklick@ucsd.edu

CO-PRINCIPAL INVESTIGATORS
Razelle Kurzrock, MD
Sr. Deputy Center Director, Clinical Science,
Director, Center for Personalized Cancer Therapy,
UC San Diego Moores Cancer Center,
3855 Health Sciences Drive
La Jolla, CA 92093
rkurzrock@ucsd.edu

Brian Leyland-Jones, MD, PhD
Avera Cancer Institute
1000 E. 23rd Street, Suite 370
Sioux Falls, SD 57105
Brian.LeylandJones@avera.org

Casey Williams, PharmD, BCOP
Avera Cancer Institute
1000 E. 23rd Street, Suite 370
Sioux Falls, SD 57105
Casey.Williams@avera.org

BIOSTATISTICIAN
J. Jack Lee, Ph.D.
Professor, Department of Biostatistics
University of Texas M.D. Anderson Cancer Center
1400 Pressler Street
Houston, Texas 77030
Tel: 713-794-4158
Fax: 713-563-4243
jjlee@mdanderson.org

TABLE OF CONTENTS

STUDY SCHEMA **5**

STUDY SUMMARY **6**

1.0 BACKGROUND AND RATIONALE **11**

1.1 CANCER THERAPY BACKGROUND 11
1.2 ONCOGENE-DRIVEN TARGETED THERAPY 14
1.3 RATIONALE 15
1.4 STUDY OBJECTIVES 16
1.5 ENDPOINTS 17

2.0 PATIENT ELIGIBILITY **18**

2.1 INCLUSION CRITERIA 18
2.2 EXCLUSION CRITERIA 20

3.0 STUDY PLAN **20**

3.1 STUDY OVERVIEW 20
3.2 NAVIGATION TO TREATMENT 23
3.3 DOSING DELAYS/DOSE MODIFICATIONS 36
3.4 PERMITTED CONCOMITANT THERAPY 36
3.5 PROHIBITED CONCOMITANT THERAPY 36
3.6 OTHER MODALITIES OR PROCEDURES 36
3.7 DURATION OF FOLLOW UP 36
3.8 PATIENT REPLACEMENT 36

4.0 STUDY PROCEDURES **37**

4.1 SCREENING/BASELINE PROCEDURES 37
4.2 PROCEDURES DURING TREATMENT 39
4.3 FOLLOW-UP PROCEDURES 40
4.4 REMOVAL OF SUBJECTS FROM STUDY 40

5.0 MEASUREMENT OF EFFECT **40**

5.1 FEASIBILITY 40
5.2 TUMOR RESPONSE 40
5.3 CHANGE IN RESECTABILITY 40
5.4 ADVERSE EVENTS 41

6 STATISTICAL CONSIDERATIONS **43**

6.1 STUDY DESIGN 43
6.2 SAMPLE SIZE AND ACCRUAL 44
6.3 STATISTICAL METHODS 44
6.4 SAMPLE SIZE CALCULATION 44
6.5 PLANNED ANALYSES 45
6.6 EXPLORATORY ANALYSES 46

7 STUDY MANAGEMENT **48**

7.1 CONFLICT OF INTEREST 48
7.2 INSTITUTIONAL REVIEW BOARD (IRB) APPROVAL AND CONSENT 48
7.3 SUBJECT DATA PROTECTION 49
7.4 DATA AND SAFETY MONITORING 49
7.5 RECORD RETENTION 49
7.6 OBLIGATIONS OF INVESTIGATORS 50

8 REFERENCES **51**

9 APPENDICES 52
APPENDIX A. RESPONSE EVALUATION CRITERIA IN SOLID TUMORS (RECIST) 52

Abbreviations	
**ALCHEMIST**	Adjuvant Lung Cancer Enrichment Marker Identification and Sequencing Trials
**CR**	Complete response
**CT**	Computed tomography
**DSMB**	Data and Safety Monitoring Board
**FFPE**	Formalin-fixed paraffin-embedded
**I-PREDICT**	Investigation of Profile-Related Evidence Determining Individualized Cancer Therapy
**MR**	Magnetic resonance
**NCDB**	National Cancer Database
**NCI**	National Cancer Institute
**OS**	Overall survival
**PD**	Progressive disease
**PET**	Positron emission tomography
**PFS**	Progression-free survival
**PR**	Partial response
**RECIST**	Response Evaluation Criteria in Solid Tumors
**SD**	Stable disease
**TTP**	Time to progression
**WIN**	Worldwide Innovative Networking (WIN)

STUDY SCHEMA

The schema represents a broad overview of the study design; please see Section 3 for more detail. This is an open label **navigational** investigation of molecular profile-related evidence used to determine individualized cancer therapy for patients with incurable malignancies. This will serve as a **feasibility** study in incurable malignancies, and consist of three patient groups (i.e., Groups 1, 2, and 3) and two treatment arms (i.e., Arms A and B).

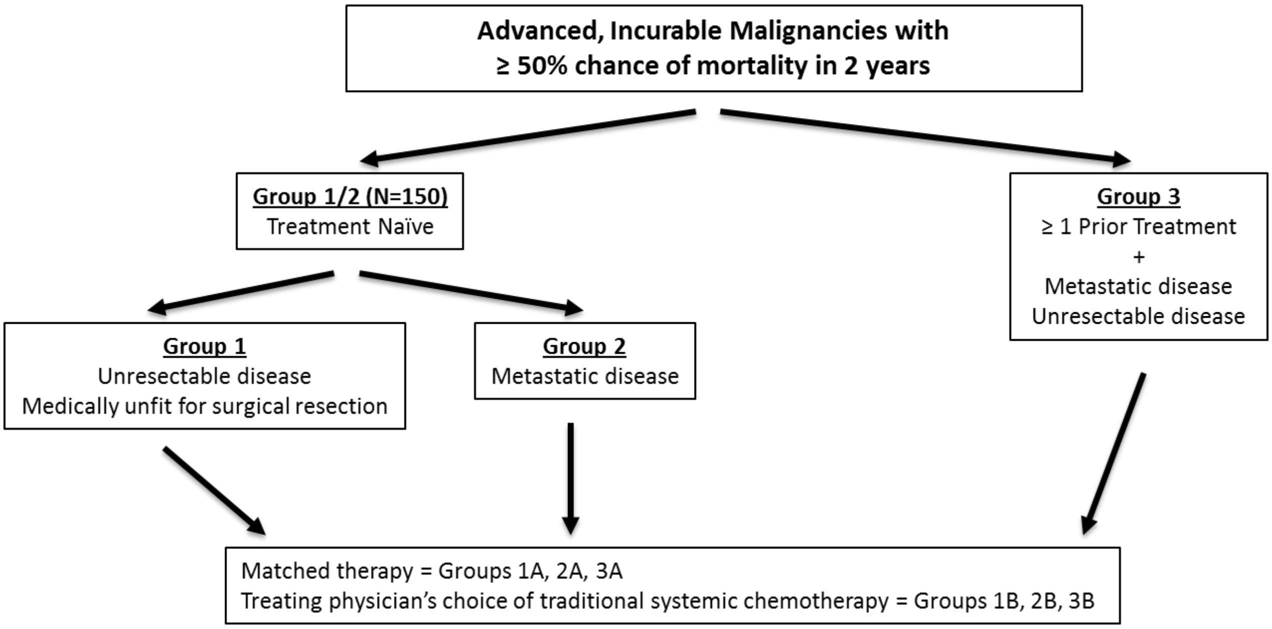

STUDY SUMMARY

Title	An open label navigational Investigation of molecular Profile-Related Evidence Determining Individualized Cancer Therapy for patients with incurable malignancies
Short Title	*I*-PREDICT
Phase	Feasibility
Methodology	Open label, non-randomized
Study Duration	3 years (2.5 years of accrual + 0.5 year of additional follow-up)
Study Center(s)	Multicenter
Objectives	Goal: To perform a prospective, descriptive feasibility study that is a histology-independent personalized navigation approach to cancer therapy based upon tissue/blood from primary tumor or metastases to be analyzed by Foundation Medicine's FoundationOne™ or FoundationOne Heme ™, or FoundationACT™ genomic analysis. Included are groups conducted as a window of opportunity trial for treatment of naïve patients (Group 1 and Group 2), as well as a group of previously treated patients (Group 3).<br>**Primary Objective:**<br>To determine the feasibility of using molecular testing to determine targeted therapy for patients with newly diagnosed (Groups 1 and 2) and previously treated (Group 3) cancers with incurable biology (≥50% 2-year cancer-associated mortality) after their treating physician receives recommendations based upon genomic analysis for molecular alterations.<br>**Secondary Objectives:**<br>Assess the:<br>• ability to enroll patients in Groups 1, 2 and 3<br>• turnaround time for genomic analysis<br>• ability to identify actionable genomic alterations<br>• ability of treating physicians to wait for molecular information in order to start treatment<br>• ability to obtain insurance coverage in order for patients to receive molecularly targeted matched treatment based on genomic analysis<br>**Exploratory Objectives:**<br>Assess the:

	• activity of molecularly targeted matched treatment and physician's choice of traditional systemic chemotherapy treatment<br>• incidence of serious adverse events in molecularly targeted matched treatment and physician's choice of traditional systemic chemotherapy treatment
Endpoints	Primary Endpoint:<br>The proportion of patients who receive molecularly targeted matched treatment after recommendations based on genomic analysis<br>Secondary Endpoints:<br>Determine the:<br>1. Proportion of patients that consent to all patients approached for the study<br>2. Time from informed consent to the receipt of FoundationOne™ genomic analysis<br>3. Proportion of patients with actionable genomic alterations<br>4. Proportion with insurance coverage for receiving molecularly targeted matched treatment based on genomic analysis<br>Exploratory Endpoints<br>Estimate the:<br>a. overall response rate (ORR) defined as partial response (PR) or complete response (CR) occurring at any point during treatment according to Response Criteria in Solid Tumors (RECIST) 1.1 in all treatment groups<br>b. regression rate as a percent defined by best percent size reduction in target lesions from baseline to discontinuation in all groups<br>c. progression free survival (PFS) defined as time from first dose to disease progression or death whichever occurs first<br>d. overall survival (OS) defined as time from first dose to death due to any cause<br>e. resection rate as a percent of patients previously considered unresectable that are able to be resected at 8 weeks or at any later time in all groups<br>f. change in biochemical tumor markers of disease as defined as the best percent change from baseline in relevant tumor markers from first dose to discontinuation<br>g. incidence of serious adverse events in all groups according to CTCAE v4.03

Number of Subjects	1150
Diagnosis and Main Inclusion Criteria	Patients with incurable malignancies (defined as ≥50% 2-year cancer-associated mortality as deemed by 2 oncologists and according to 2014 NCDB data in Figure 1), with at least one of the following:<br>• Metastatic disease<br>• Unresectable disease<br>• Medically unfit for surgical resection but with an expected survival of > 3 months, ECOG < 2 and NYHA status ≤ II<br>More specifically:<br>Group 1: incurable biology without prior systemic therapy (i.e., treatment naïve): Unresectable disease, medically unfit for surgical resection.<br>Group 2: incurable biology without prior systemic therapy (i.e., treatment naïve): Metastatic disease.<br>Group 3: incurable biology with ≥ 1 prior unmatched systemic therapy: Unresectable disease (as deemed by 2 surgeons), or medically unfit for surgical resection.<br>Furthermore, patients in each group will be classified to two arms based on treatment type received:<br>• Arm A: Matched therapy<br>• Arm B: Unmatched therapy (treating physician's choice of traditional systemic chemotherapy)
Study Intervention	Recommendation of personalized targeted therapy based upon the individual's tumor genomic analysis (Foundation Medicine's FoundationOne™ assay) for molecular alterations.
Duration of administration	Arm A<br>• Groups 1 and 2 = ~ 8 week cycles of a regimen of ≥ 1 drug that targets molecular alteration(s) followed by disease appropriate imaging (i.e., PET-CT vs. CT vs. MR imaging) followed by crossover to treating physician's choice of systemic chemotherapy for SD or PD per RECIST, or unacceptable toxicity but to remain on I-PREDICT recommended regimen if CR, PR by RECIST 1.1<br>• Group 3 = ~ 8 week cycles of a regimen of ≥ 1 drug that targets molecular alteration(s) followed by disease appropriate imaging (i.e., PET-CT vs. CT vs. MR imaging) with cessation of therapy if PD or unacceptable toxicity<br>Arm B

	1. treating physician's choice of traditional systemic chemotherapy = ~ 8 week cycles for Groups 1, 2, and 3.<br><br>Continue until:<br>• Progressive disease<br>• Unacceptable toxicity<br>• Complete response x 6 months<br>• Deemed resectable
Statistical Methodology	**Groups 1, 2, and 3:**<br>• Determine the feasibility of the genomic-based individualized targeted therapy in patients with incurable malignancies in all patients and in each of the patient groups.<br>• Determine the response rate [(biochemical = tumor markers; radiographic = RECIST)]<br>• Determine the tumor regression rate (%) among groups in order to determine if there is a higher rate in the treatment-naïve groups (i.e., Groups 1 and 2) than in the previously treated group (Group 3)<br>• Determine progression-free survival (PFS) and overall survival (OS)<br>• Determine serious adverse events<br>**Group 1 and Group 2:**<br>• The treatment efficacy in the matched cohorts (i.e., Groups 1A and 2A) and the cohort of patients receiving treating physician's choice of traditional systemic chemotherapy [e.g., no drug(s) available for target(s), no insurance coverage for recommended drug(s) = Group 1B and 2B, respectively] will be described.<br>• For the unresectable patients, determine the resectability rate in Groups A and B at 8 weeks and if ever resected.<br>**Group 3:**<br>1. For patients in 3A and 3B, we will assess the PFS of the last unmatched therapy (PFS1) versus the PFS of the current therapy (PFS2). We will estimate the percent of patients with PFS2/PFS1 > 1.5.<br>2. The treatment efficacy in the matched cohort (i.e., matched cohort = 3A) and the cohort of patients receiving treating physician's choice of traditional systemic chemotherapy [i.e., no drug(s) available for target(s), no insurance coverage for recommended drug(s) = Group 3B] will be described.

1.0 BACKGROUND AND RATIONALE

Cancer Therapy Background

The paradigms for treating advanced malignancies have drastically changed over the past 60 years. Historically, chemotherapeutic agents have been used to treat numerous types of cancers. Scientists are now attempting to use their growing knowledge of cancer biology to create targeted therapies that are less harmful to benign cells and more deadly to cancer cells. Targeted therapies may be less harmful to the body because they are more specific in their targeting of cancer cells. Although many people receiving conventional chemotherapy still experience “classic” side effects such as nausea, fatigue, hair loss, and an increased susceptibility to infections, thanks to modern techniques, these side effects are no longer universal to all cancer treatments.

Although it began, like so many discoveries, as serendipity, the science of chemotherapy continues to evolve and improve. The inception of modern chemotherapy for treating cancer can be linked to the discovery of nitrogen mustard, a chemical warfare agent developed in the 1940s. Two pharmacologists, Louis Goodman and Alfred Gilman, were recruited by the U. S. Department of Defense to study the potential application of chemical warfare agents for therapeutic purposes. Based upon autopsies of individuals exposed to mustard gas, they “accidentally” discovered profound suppression of white and red blood cells. Together, they hypothesized that this agent could be used to treat lymphoma. Following World War II, a novel approach to treating cancer was investigated by Sidney Farber, a pathologist at Harvard Medical School. He studied the effects of folic acid, a vitamin crucial for DNA synthesis and repair, in children with leukemia and discovered that it stimulated the leukemia cells to divide. This led to the development of a drug that could block this mechanism. Together with collaborators, Dr. Farber used methotrexate, an anti-folate drug, to induce remission in children with leukemia. In 1958, this same drug cured a patient with choriocarcinoma, a solid tumor that originates in the placenta. This was the first solid tumor cured by chemotherapy. Seven years later, the investigators James Holland, Emil Freireich, and Emil Frei suggested that, like the treatment for tuberculosis, chemotherapy should be given in combination rather than as a single agent. The goal was to attack the cancer from several different angles. With time, physicians realized that these treatments worked well for small tumors but not for larger ones and started using chemotherapy in combination with surgery. Medical oncologists prescribing chemotherapy regimens soon hit a wall. Despite President Nixon’s declaration of a war on cancer with the National Cancer Act of 1971, patients were still dying of their tumors. Many tumors were found to be resistant to chemotherapeutic agents. In fact, some regimens merely resulted in side effects without providing benefit because the drugs’ aims were too broad. To these drugs, certain benign cells, such as those in the intestine and hair follicles, looked just like malignant ones. In line with President Nixon’s vision, the 1980s and 1990s saw a cancer treatment renaissance. While clinicians made many discoveries, a significant number of treatment trials were being blazed in the laboratory. Scientists began studying the mechanisms underlying cancer cell biology so that they could “snipe” instead of “carpet bomb.” Up to this point, chemotherapeutic agents had been discovered essentially by chance, or by inhibiting the metabolic pathways thought to be crucial to cell division. But with new understanding of the cross-talk within normal cells and cancer cells, chemotherapeutic bullets could be aimed at the signaling networks that were allowing only the cancer cells to proliferate and survive.

The perfect example of such targeted drug development was imatinib mesylate (Gleevec®, Novartis, Pharmaceutical, Basel Switzerland). The use of imatinib in the treatment of chronic myelogenous leukemia (CML) transformed the disease by rapidly changing the median survival from 4 years to more than 20 years.[1] The underlying reasons for this revolution included: 1) the identification of a critical driving molecular alteration, namely *Bcr-Abl*; and 2) the development of a potent and specific *Bcr-Abl* inhibitor, imatinib.[2] It turns out that this small molecule also inhibits *c-KIT*, a tyrosine kinase signaling molecule. What makes *c-KIT* so special is that 90% of Gastrointestinal Stromal Tumors (GISTs) contain an activating *KIT* mutation. GIST is now recognized as the most common sarcoma of the gastrointestinal tract. It is believed that there are up to 6,000 new cases of GIST diagnosed each year in the United States alone. The application of imatinib in the treatment of GIST reflects a major advance in the treatment of all solid tumors with specific molecular targeting. In fact, matching patients to this targeted therapy is solely based upon actionable *KIT* genomic alterations and has clearly led to anti-tumor activity. Imatinib, is now the first-line treatment for metastatic, unresectable, recurrent, and resected high-risk GIST.[3]

Perhaps equally important as having the right drug for the right target was the timing of the targeted therapy; specifically, its administration to patients with newly diagnosed disease rather than advanced disease. Indeed, the response rates of patients with CML that are in blast transformation are only about 15% and all patients relapse; the median survival has increased from 6 months to about 12 months with single-agent BCR-ABL inhibitors.[1] In contrast, with the late use of these inhibitors, median survival of newly-diagnosed CML has increased from 4 years to 20-25 years. Thus, earlier treatment in the disease process has been a transformative change in this hematological malignancy. Until 2009, the same could not be said for any solid tumor. All of the available randomized controlled clinical trials in the treatment of GIST addressed the use of imatinib in advanced (surgically unresectable or metastatic) disease. Whether it was beneficial following earlier in the disease's natural history was unknown. However, in 2009, the American College of Surgeons Oncology Group, published work in "The Lancet" demonstrating the results of the first randomized, controlled trial addressing the role of imatinib in the postoperative period. Interim analysis demonstrated that daily imatinib administered for one year after complete resection of localized GIST provided a reduction in disease recurrence when compared with placebo. Thus, this study clearly demonstrated that postoperative imatinib reduced the rates of early recurrence.[4] This is now standard of care and opened the doors to earlier applications for targeted agents in solid tumors.

Taken together, physicians now take into account the tumor's genomic alteration status, as well as disease progression, possible surgery, and targeted therapy when framing a more multidisciplinary approach to treatment that can include radiologists, pathologists, medical oncologists, and surgeons.

The impact of chemotherapy on the survival of patients with cancers has not yet met the goals set by President Nixon. Cancer remains a major cause of morbidity and mortality in the United States. Unfortunately, many forms of traditional chemotherapy are still ineffective against many cancers. We aim to develop a methodology for targeted, personalized therapies following the GIST and CML paradigms in other types of cancers.

To date, molecular matching has been performed in PREDICT and now WINther, which is IRB approved at UCSD, MD Anderson, and four other sites, will match patients. Therefore, there is precedent for this methodology. Earlier studies reported by Dr. Kurzrock and colleagues have shown that this approach can be can be performed in late stage diseases and have yielded critical insights into personalized cancer therapy.[6, 8] However, the methodology of I-PREDICT is different from what is already in practice. While the protocol for previously treated patients is

similar to the manner in which one might currently treat patients that have previously progressed on other therapies, such an approach is not being utilized early in disease courses of incurable malignancies. Thus, the treatment-naïve patients in our study would not routinely be offered matched therapy at present. As such, the previously described feasibility remains unknown; previously treated patients serve as a control group in our study. While numerous publications on genomic alterations in cancer have been reported, our particular approach is novel. To our knowledge, the only other trials with a similar approach are I-SPY2 in breast cancer and Adjuvant Lung Cancer Enrichment Marker Identification and Sequencing Trials (ALCHEMIST) in early stage lung cancer. More specifically, I-SPY 2 uses novel agents, including phase I drugs, in newly diagnosed breast cancer patients with a risk of relapse of 30% at 10 years; 60% of patients would be expected to be cured in this study. In contrast, our study examines, for the first time, newly diagnosed patients with diseases other than breast cancer. In comparison, we have set a much higher bar for entry—that is patients with ≥50% chance at death at 2 years; these patients have negligible cure rates (**Figure 1**). Meanwhile, ALCHEMIST is supported by the NCI while coordination of the component trials is by the Alliance for Clinical Trials in Oncology and the ECOG-ACRIN Cancer Research Group. The three new NCI trials collectively known as ALCHEMIST aim to identify early-stage lung cancer patients with tumors that harbor certain uncommon genomic changes and evaluate whether drug treatments targeted against those molecular changes can lead to improved survival. Therefore, the NCI is supporting a genomically driven matching trial in patients with earlier stage lung cancer, despite the absence of randomized data. This further confirms that bringing genomically matched therapy to early stage patients with poor prognoses is a strategy of interest for clinical research.

Figure 1. Two-year mortality rates for cancers according to the 2014 National Cancer Database (NCDB). Red box indicates eligible patients.

	Stage III 2-yr Mortality	Stage IV 2-yr Mortality
Gallbladder	89.5%	94.8%
Pancreas*	86.5%	93.3%
Liver	83.0%	93.3%
Intrahepatic bile duct	79.1%	92.8%
Esophagus	70.6%	90.3%
Bile duct (other)	70.5%	92.2%
Lung, Bronchus - Non small cell carcinoma	65.3%	88.7%
Stomach	63.9%	90.0%
Small intestine	37.1%	70.7%
Ovary	35.2%	60.8%
Urinary*	34.2%	69.9%
Soft tissue sarcoma including heart	31.9%	72.0%
Melanoma	24.1%	77.8%
Breast*	14.2%	52.1%
Colorectal	13.4%	58.6%

** UCSD-specific data; other others are all NCDB cases*

Oncogene-driven Targeted Therapy

Current cancer therapeutics are based upon population statistics by disease site and histology and lead to standard protocols prescribed in consecutive lines (i.e., first, second, and third). However, today there are also several models for selecting molecular targeted therapies based upon tumoral molecular abnormalities (i.e., mutations, insertions, deletions, rearrangements, or gene amplifications). Examples of oncogene-driven therapy include:

- EGFR mutation : afatinib, erlotinib, gefitinib;
- BCR-ABL translocation : imatinib;
- KIT mutation: imatinib and sunitinib;
- HER2 amplification: trastuzumab and lapatinib
- B-RAF mutation: vemurafenib;
- ALK rearrangement: crizotinib;
- PI3K and mTOR pathway: everolimus and temsirolimus;
- SMO mutations : vismodegib;
- TP53 mutations: bevacizumab [5];
- Additional examples (http://www.cancer.gov/cancertopics/factsheet/Therapy/targeted).

Historically, only 10-40% of tumors had known “oncogenic drivers.” Thus, for the vast majority of tumors, possible genomic alterations were unknown. Therefore, therapeutic decisions were made according to decades-old treatment protocols that are unsatisfactory at the individual patient level, because more than 50% of patients do not respond to standard chemotherapeutic regimens. But, even for tumors with an oncogenic event identified using outdated genomic analyses, the match with selected therapeutics does not predict efficacy or clinical benefit in about half of patients. However, with next generation sequencing and our increasing understanding of cancer genomics, we may be able to improve upon these statistics.

As of October 2013, Foundation Medicine, Inc. provides a CLIA-certified assay called FoundationOne™ that utilizes comprehensive next generation sequencing of routine formalin-fixed paraffin-embedded (FFPE) tissue or blood specimens. The test simultaneously sequences the entire coding sequence of 236 cancer-related genes plus 47 introns from 19 genes often rearranged or altered in cancer. **The average turnaround time for the test to be completed is currently 14-17 days from receipt of the sample.**

The current study will perform tumor molecular profiling through FoundationOne™ to navigate patients to a matched therapy at the discretion of their treating physician. For those patients with an actionable tumor alteration, matched therapy will be recommended by the Study Committee (or Molecular Tumor Board) from either FDA approved drugs or matched agents under investigation in a separate clinical trial. Those patients, for whom there are no available matched treatments, will serve as the unmatched therapy controls.

Patients with no actionable alterations by FoundationOne***™*** will be considered to have completed their participation in the study. Their basic data will be captured including demographics and tumor type and accounted into the denominator for feasibility analysis.

Rationale

<u>Rationale for Group 3:</u> *Use of FoundationOne*^{TM} *panel to match patients with previously treated advanced disease to targeted therapies*. In solid tumors, targeted therapies are often both developed and applied in metastatic malignancies after conventional approaches have failed. In this stage, tumors often have heterogeneous cell populations and their biology is increasingly complex. Therefore, it is crucial to have comprehensive molecular testing in order to identify the underlying multiple alterations in each tumor. As in CML, detecting and targeting a single alteration may induce responses and increase survival. However, it is unlikely to result in dramatic improvements because single gene testing is not adequate to predict the behavior of these complex tumors and the majority of cases approached with "hot spot" testing will not identify a driver alteration. In line with this, we have previously performed and published on the use of molecular testing in this context. Our Phase I trial of targeted agents was designated PREDICT, and we were able to demonstrate higher than expected response rates in the advanced, refractory metastatic setting even with a panel of 12 genes.[6] In fact, response rates were 27% in patients with heavily pretreated metastatic disease and one mutation that were administered a targeted therapy that was matched for their mutation (as compared to 5% when they were not matched). Moreover, the survival was 4 months longer in the matched group versus the unmatched group (non-randomized trial; $P<0.05$). Progression-free survival was also longer in the phase I matched treatment setting, as compared to the patients' own previous conventional therapy ($P<0.05$). This trial therefore showed modest, but real, improvements, and formed a proof-of-principle for molecular matching and matched therapy.

However, as mentioned, the original PREDICT trial was limited by the molecular assays available at that time—12 single gene assays. Inevitably, small amounts of biopsy material meant that most patients could have only 3-5 of the 12 tests analyzed; furthermore, performing single gene tests was labor intensive, time consuming, and expensive. Most importantly, such a panel of only 12 genes is now known to represent only a minute fraction of the genes involved in tumor maintenance and progression. When more comprehensive molecular assays are exploited, patients with metastatic disease are often found to have multiple genomic alterations in their tumors rather than the single alteration or no alteration generally detected with far less comprehensive surveys of the cancer genome. With the current comprehensive FoundationOne^{TM} molecular test, prospective evaluations of its utility are crucial for individualizing the administration of targeted agents in incurable neoplasms. In summary, as compared to earlier studies, the current study will have increased sequencing capabilities, increased knowledge of actionable genomic alterations, and increased access to drugs to target these genomic alterations, making Group 3 an essential part of the trial.

<u>Rationale for Group 1 and Group 2:</u> *Use of FoundationOne*^{TM} *genomic profiling to match treatment naïve patients with targeted therapies*. It may also prove important to utilize molecular testing earlier in the course of the disease, when tumors are less complex, and targeting their alteration(s) could conceivably have a transformational impact. As mentioned above, moving imatinib therapy from late-stage CML to newly diagnosed disease revolutionized outcomes for this type of leukemia. Instead of inevitable death in half of patients at 4 years after diagnosis, patients now have a near-normal life expectancy. In solid tumors, matched targeted therapy has not yet been applied in newly diagnosed disease despite the fact that treatment naive solid cancers are much less heterogeneous earlier than later in their disease natural history. Furthermore, as a general principle, strategies/therapies that show proof-of-principle efficacy in late-stage malignancies can be expected to demonstrate significantly higher response rates and survival impact in early disease.

Designing such a trial is complicated because one must take into consideration conventional therapies. **However, the I-SPY trial in breast cancer has already set the precedent for introducing novel, and even experimental drugs, into patients with newly-diagnosed, high-risk breast cancer. In that trial, MammaPrint is used to define high-risk; a high-risk test result means that the cancer has a 29% risk of relapsing within 10 years without any additional treatments after surgery.** However, I-SPY uses Bayesian statistics to allocate patients to treatment. As a result, a patient with a BRCA mutation may or may not receive a PARP inhibitor despite our knowledge that these work in this patient population. With the I-PREDICT trial, we propose the use of molecular profiling (FoundationOne™ assay) and matching patients to drugs based on the target(s) that exist in their tumor. For this trial, we will employ a more conservative definition of high-risk than was used in I-SPY. That is, patients who have a 50% or higher risk of death within two years (as estimated by their physician and according to 2014 NCDB data in Figure 1), or patients that already have locally advanced or metastatic disease at the time of initial diagnosis. Thus, the I-PREDICT clinical trial employs an innovative method to estimate potential efficacy of therapeutics in order to guide the choice of therapies for each individual patient in the early or late phases of disease. This trial addresses unmet needs.

We hypothesize the following:

- Obtaining treatment with targeted therapy after the treating physician receives recommendations based upon actionable genomic alteration(s) will be feasible for treatment naïve patients.
- Patients who receive targeted therapy based upon recommendations from actionable genomic alteration(s) will yield anti-tumor activity.
- Eight-week regression rates will be higher in treatment naïve patients than in previously treated patients.
- Overall survival in treatment naïve patients will be greater than expected according to historical controls matched for stage and other known prognostic factors.
- In previously treated patients, the progression-free survival on matched therapy will be greater than on their last unmatched therapy.
- Patients who receive targeted therapy based upon recommendations from actionable genomic alteration(s) will demonstrate conversion of unresectable to resectable disease.

I-PREDICT may provide feasibility and response information that will be fundamental for designing definitive randomized trials that may change the paradigm of cancer therapy. Eventually, if clinically validated, the data would enable physicians and molecular biologists to select the optimal drug (or drug combination) for each patient based upon their personal tumor genomics. The new approach aims to address the prediction of efficacy of chemotherapeutics or targeted agents (approved or in clinical trial) at an individual level. We will look for known alterations by next generation sequencing at Foundation Medicine (DNA based). If there is a known oncogenic event, the matched targeted therapy will be recommended.

Study Objectives

Below is a detailed description of Primary and Secondary objectives of the study.

Primary Objectives

To determine the feasibility of using molecular testing to determine targeted therapy for patients with newly diagnosed (Groups 1 and 2) and previously treated (Group 3) cancers with incurable biology (≥50% 2-year cancer-associated mortality as estimated by two oncologists and where appropriate according to 2014 NCDB data in Figure 1) after their treating physician receives recommendations based upon genomic analysis for molecular alterations.

Secondary Objectives:
Assess the:
1. Ability to enroll patients in Groups 1, 2 and 3
2. Turnaround time for genomic analysis
3. Ability to identify actionable genomic alterations
4. Ability of treating physicians to wait for molecular information in order to start treatment
5. Ability to obtain insurance coverage in order for patients to receive molecularly targeted matched treatment based on genomic analysis

Exploratory Objectives:
Assess the:
1. Activity of molecularly targeted matched treatment and physician's choice of traditional systemic chemotherapy treatment
2. Incidence of serious adverse events in molecularly targeted matched treatment and physician's choice of traditional systemic chemotherapy treatment

Endpoints

Primary Endpoint:
The proportion of patients who receive molecularly targeted matched treatment **after recommendations** based on genomic analysis

Secondary Endpoints:
Determine the:
1. Proportion of patients that consent of all patients approached for the study
2. Time from informed consent to the receipt of FoundationOne^{TM} genomic analysis
3. Proportion of patients with actionable genomic alteration
4. Proportion with insurance coverage for receiving molecularly targeted matched treatment based on genomic analysis

Exploratory Endpoints
Estimate the:
1. Overall response rate (ORR) defined as partial (PR) or complete response (CR) occurring at any point during treatment according to Response Criteria in Solid Tumors (RECIST) 1.1 in all treatment groups
2. Regression rate as a percent defined by best percent size reduction in target lesions from baseline to discontinuation in all groups
3. Progression free survival (PFS) defined as time from first dose to disease progression or death, whichever occurs first
4. Overall survival (OS) defined as time from first dose to death due to any cause
5. Resection rate as a percent of patients previously considered unresectable that are able to be resected in all groups
6. Change in biochemical tumor markers of disease as defined as the best percent change from baseline in relevant tumor markers from first dose to discontinuation
7. Incidence of serious adverse events in all groups according to CTCAE v4.03

2.0 PATIENT ELIGIBILITY

Inclusion Criteria

Subjects must meet all of the inclusion criteria to participate in this study.

1. Patients with incurable malignancies with ≥50% 2-year cancer-associated mortality (as estimated by two physician and where appropriate according to 2014 NCDB data in Figure 1). Diseases include, but are not limited to:

Ampullary carcinoma	Intrahepatic cholangiocarcinoma (IHCC)
Appendiceal cancer	Melanoma
Colorectal cancer (CRC)	Non-KIT GIST (gastrointestinal stromal tumor)
Extrahepatic cholangiocarcinoma (EHCC)	Non-small cell lung cancer (NSCLC)
Esophageal adenocarcinoma	Ovarian cancer
Gallbladder cancer (GBCA)	Pancreatic ductal adenocarcinoma (PDAC)
Gastric adenocarcinoma	Sarcoma (high-grade)
Head and neck cancer	Small bowel adenocarcinoma (including duodenal)
Hepatocellular carcinoma (HCC)	Triple-negative breast cancer (TNBC)
	Urothelial cancer

   * Note: We will limit gastrointestinal malignancies to no more than 65% of the case mix.

2. Patients with cancer of unknown primary or a rare tumor (i.e., fewer than 15 cases per 100,000 per year) with no approved therapies. (Patients in this inclusion criteria must meet all other exclusion and inclusion criteria except inclusion criteria #1)

3. Patients with incurable malignancies, irrespective of 2-year mortality, who, in the opinion of the investigator have no treatment option expected to yield significant clinical benefit."

4. Patients must have at least one of the following for a diagnosis/disease status:
   a. Unresectable disease
   b. Metastatic disease
   c. Medically unfit for surgical resection but with an expected survival of > 3 months, ECOG < 2 and NYHA status ≤ II (refer to status definitions in tables below)
   d. Disease where no conventional therapy leads to a survival benefit > 6 months in the respective cohort and line of therapy for which the patient is otherwise eligible
   e. Actionable alterations determined by FoundationOne™

5. Treatment naïve for his or her newly diagnosed malignancy for enrollment to Groups 1 or 2.

6. Status post 1 or more unmatched systemic therapy regimens for enrollment to Group 3.

7. Ability to understand and the willingness to sign a written informed consent.

8. Patients must have measurable disease for malignancies: defined as at least one lesion that can be accurately measured in at least one dimension (longest diameter to be recorded for non-nodal lesions and short axis for nodal lesions) as ≥20 mm with conventional techniques or as ≥10 mm with spiral CT scan, PET-CT, MRI, or calipers by clinical exam.

9. Age ≥ 18 years.

10. ECOG Performance Status 0-1.

Grade	Description
0	Fully active, able to carry on all pre-disease performance without restriction
1	Restricted in physically strenuous activity but ambulatory and able to carry out work of a light or sedentary nature, e.g., light housework or office work
2	Ambulatory and capable of all self-care but unable to carry out any work activities. Up and about >50% of waking hours
3	Capable of only limited self-care, confined to a bed or chair >50% of waking hours
4	Completely disabled. Cannot carry on any self-care. Totally confined to bed or chair
5	Dead

11. New York Heart Association (NYHA) Functional Classification I-II.

NYHA Class	Symptoms
I	Cardiac disease, but no symptoms and no limitation in ordinary physical activity, e.g. shortness of breath when walking, climbing stairs etc.
II	Mild symptoms (mild shortness of breath and/or angina) and slight limitation during ordinary activity.
III	Marked limitation in activity due to symptoms, even during less-than-ordinary activity, e.g. walking short distances (20–100 m). Comfortable only at rest.
IV	Severe limitations. Experiences symptoms even while *at rest*. Mostly bedbound patients.

12. Adequate organ and marrow function as defined below:
    - Absolute neutrophil count ≥ 1.5 x 10^9/L
    - Platelet count ≥ 100 x 10^9/L
    - Total bilirubin ≤ 2.0 x institution's ULN
    - Patients without underlying liver disease
        - ALT and AST ≤ 2.5 X institutional upper limit of normal
    - Serum creatinine ≤ 2.0 x institution's ULN or 24-hour creatinine clearance ≥ 50 ml/min

13. At the time of treatment, patients should be off other anti-tumor agents for at least 5 half-lives of the agent or 3 weeks from the last day of treatment, whichever is shorter to enroll in Group 3. Patients must not have been treated with anti-tumor agents to enroll in Group 1 or Group 2. Patients must be off prior antibody therapy for at least 3 half-lives before starting treatment. Patients may enroll on study even while receiving treatment.

14. Able to swallow and retain oral medication if needed.

15. Patients must have evaluable tissue/blood for testing as specified by the concurrent FoundationOne™ criteria. This will be obtained during the standard of care tumor diagnosis and tumor staging evaluation.

16. Female patients of childbearing potential must have a negative serum pregnancy test and agree to use at least one form of pregnancy prevention during the study and for at least one month after treatment discontinuation. For the purposes of this study, child- bearing potential is defined as: all female patients that were not in post-menopause for at least one year or are surgically sterile (site-specific criteria applying to Avera only)

17. Male patients must use a form of barrier pregnancy prevention approved by the investigator / treating physician during the study and for at least one month after treatment discontinuation (site-specific criteria applying to Avera only).

Exclusion Criteria

Subjects meeting any of the exclusion criteria at baseline will be excluded from study participation.

1. Two oncologists disagree on prognosis or resectability.

2. Severe or uncontrolled medical disorder that would, in the investigator's opinion, confound study analyses of treatment response (i.e., uncontrolled diabetes, chronic renal disease, chronic pulmonary disease or active, uncontrolled infection, psychiatric illness/social situations that would limit compliance with study requirements).

3. Are pregnant or breast-feeding patients or any patient with childbearing potential not using adequate pregnancy prevention (site-specific criteria applying to Avera only).

3.0 Study Plan

Study Overview

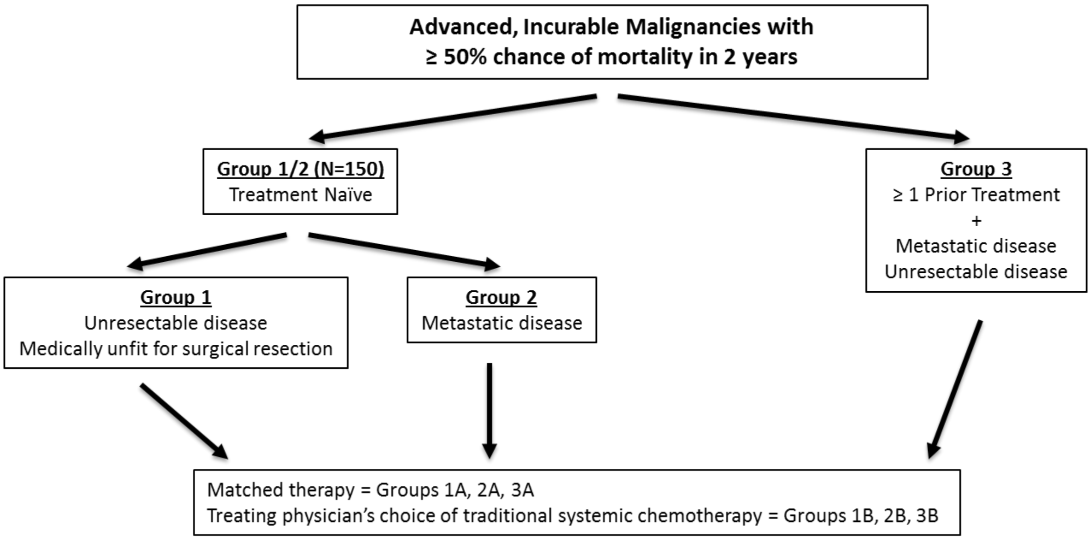

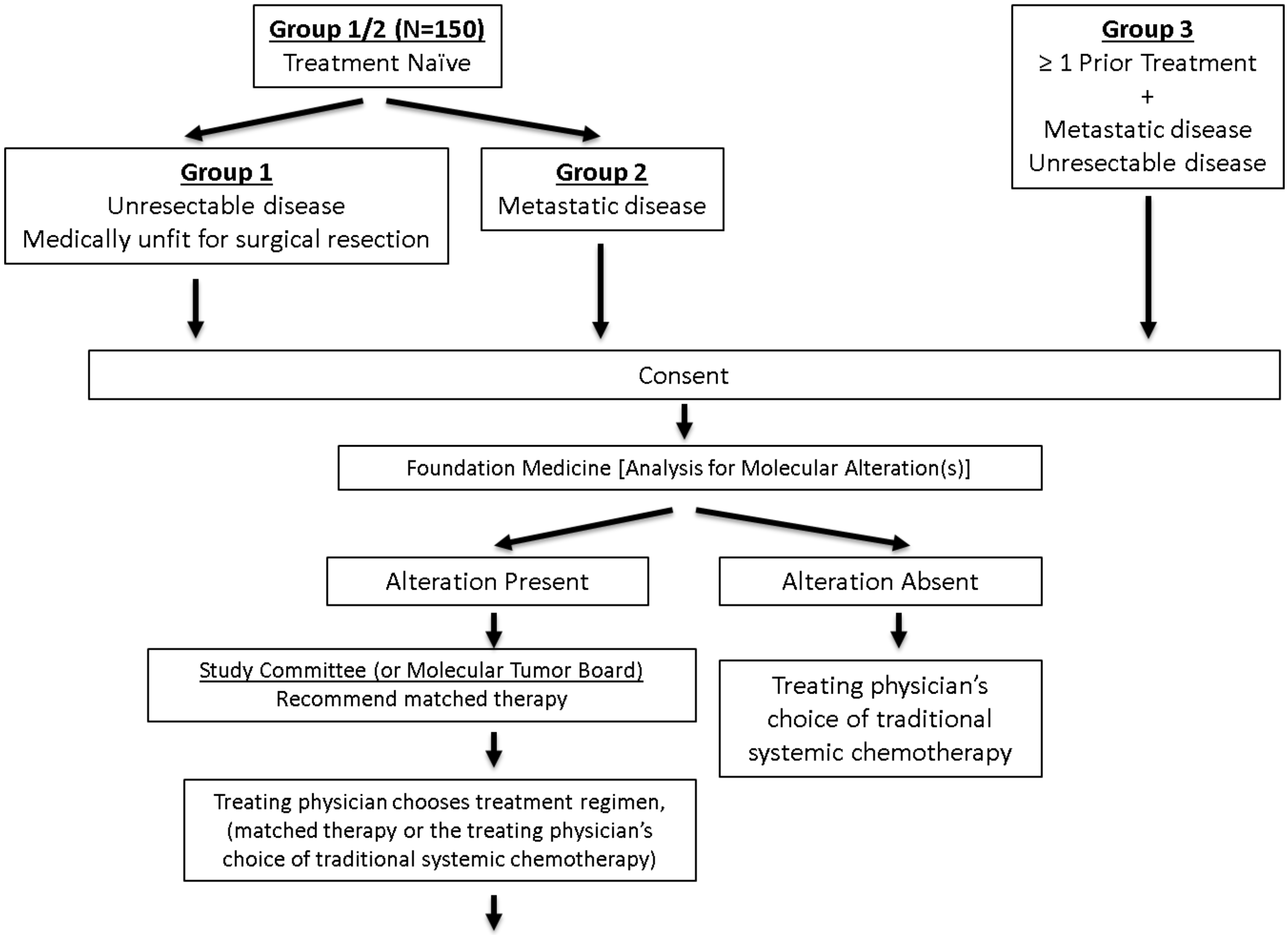
Group 1/2 (N=150)
Treatment Naïve
Group 1
Unresectable disease
Medically unfit for surgical resection
Group 2
Metastatic disease
Group 3
≥ 1 Prior Treatment
+
Metastatic disease
Unresectable disease
Consent
Foundation Medicine [Analysis for Molecular Alteration(s)]
Alteration Present
Alteration Absent
Study Committee (or Molecular Tumor Board)
Recommend matched therapy
Treating physician's choice of traditional systemic chemotherapy
Treating physician chooses treatment regimen, (matched therapy or the treating physician's choice of traditional systemic chemotherapy)

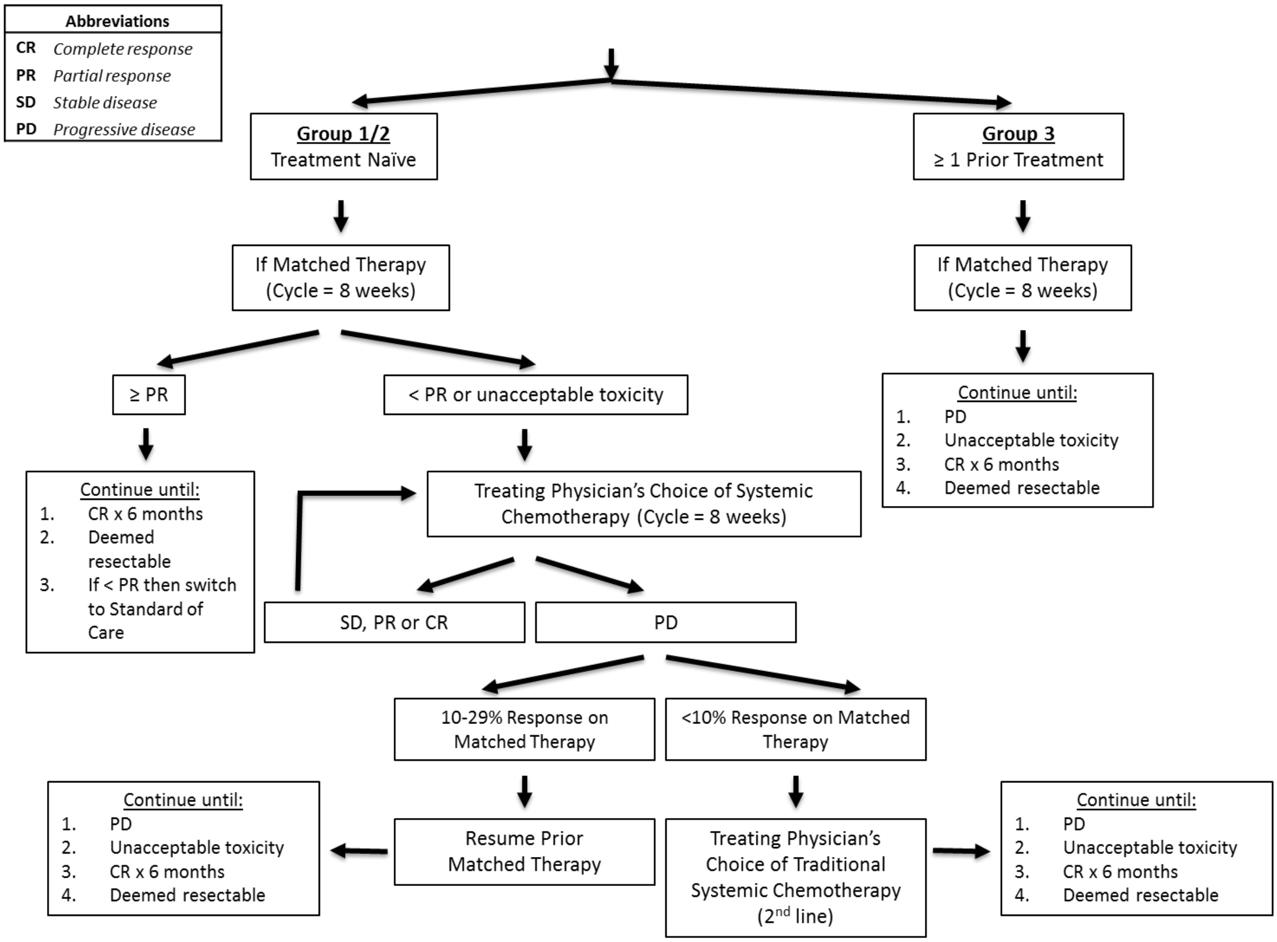
Abbreviations
CR Complete response
PR Partial response
SD Stable disease
PD Progressive disease
Group 1/2
Treatment Naïve
Group 3
≥ 1 Prior Treatment
If Matched Therapy (Cycle = 8 weeks)
If Matched Therapy (Cycle = 8 weeks)
≥ PR
< PR or unacceptable toxicity
Continue until:
1. PD
2. Unacceptable toxicity
3. CR x 6 months
4. Deemed resectable
Continue until:
1. CR x 6 months
2. Deemed resectable
3. If < PR then switch to Standard of Care
Treating Physician's Choice of Systemic Chemotherapy (Cycle = 8 weeks)
SD, PR or CR
PD
10-29% Response on Matched Therapy
<10% Response on Matched Therapy
Continue until:
1. PD
2. Unacceptable toxicity
3. CR x 6 months
4. Deemed resectable
Resume Prior Matched Therapy
Treating Physician's Choice of Traditional Systemic Chemotherapy (2nd line)
Continue until:
1. PD
2. Unacceptable toxicity
3. CR x 6 months
4. Deemed resectable

Navigation to Treatment

Study Groups

Patients will be grouped into one of three study Groups (Groups 1, 2, or 3) based on their disease status and history of prior cancer therapy:

Group 1: Treatment naïve patients with unresectable disease or medically unfit for surgical resection.

Group 2: Treatment naïve patients with metastatic disease.

Group 3: Patients with metastatic or unresectable disease who have received at least one prior unmatched systemic therapy and no prior matched therapy.

Patient and Physician Participation

Licensed and board-certified medical oncologists (who will also be sub-investigators on the study) treating patients with incurable malignancies with aggressive biology will have access to enrolling their patients in the trial. These patients will be offered one of the Foundation Medicine assays for tumor analysis. If a clinician chooses not to enroll a patient, the patient will not participate in the study even if he or she would have been otherwise eligible. Unless a practitioner chooses not to accrue patients, there will be no a priori selection for practitioners who already feel that the approach is feasible in their patient population. Every practitioner will have equal access to enrolling appropriate patients. Finally, it should be noted that this limitation applies to all trials at Moores Cancer Center and elsewhere. Physicians may always choose or not choose to enroll patients on a trial.

At the Avera McKennan site this will require a consultation to the Avera McKennan Precision Oncology clinic.

Tumor Profiling

Patients who meet the eligibility criteria and provide consent will have their tumor and/or blood profiled via Foundation Medicine's FoundationOne™ and/or FoundationACT™ assays. Available tissue/blood will constitute an adequate biopsy for molecular testing in the metastatic setting. If the patient has not had intercurrent targeted therapy, an archived diagnostic tumor sample will suffice. If the patient has received targeted therapy (Group 3), all attempts will be made to acquire a post-treatment biopsy.[7] Previous studies have shown that, in the absence of intercurrent targeted therapy, archival tissue is adequate for molecular testing and that matched treatment based on results from archival tissue is associated with significantly higher than expected response rates.[6, 8] If an eligible patient has had their tumor profiled via Foundation Medicine's FoundationOne™ assay under the auspices of routine clinical care, but they meet all criteria for enrollment to I-PREDICT, they may be consented and enrolled.

If the sample is not able to be tested or fails testing, additional sections will be sent to Foundation Medicine. If re-testing fails, a repeat biopsy may be performed if clinically appropriate or the patient may receive traditional chemotherapy. We will not delay beyond 6 weeks while awaiting Foundation Medicine testing.

Determining Actionability of Genomic Abnormalities

The process for determining actionability of genomic abnormalities relies upon the genomic alterations, mutant allele frequency, and diagnoses. This process remains in evolution. I-PREDICT assesses the combined approach of using a Molecular Tumor Board and the readings from Foundation Medicine to determine actionability, hence mimicking what practitioners would do in the community. Foundation Medicine readings are based on one of the largest databases available in the country, and include about 17,000 citations. This approach is identical to that in the IRB approved WINther protocol (approved at 6 sites, including USCD, and in 5 countries) which uses a clinical management committee as well as Foundation Medicine readings to ascertain genomic actionability. The current protocol uses the same methodology, except that the clinical management committee is called a molecular tumor board.

Alterations identified by the computational biology analysis are presented in the form of a variant report. Variant reports are routed to Foundation Medicine, Inc.'s proprietary content curation pipeline for report content compilation. Interpretive text for each alteration is prepared based on review of applicable scientific literature. The alteration interpretation addresses the following:

- Role of the affected gene in normal and tumor biology.
- Impact of the observed alteration on the function of the affected gene, if known.
- Prognostic implications of the alteration in the patient's tumor type, if any.
- Therapeutic implications of the alteration in the patient's tumor type, if any, including targeted therapies currently in development.

Foundation Medicine, Inc. also provides annotation that lists potential targeted therapies based on the genomic profile of the patient's tumor as follows:

- Targeted therapies for which the observed alteration implies either sensitivity or resistance in the patient's tumor type are listed, if available. Therapies are presented in separate groups based on their FDA approval status for the patient's tumor type. Only therapies that are FDA approved for at least one oncology indication are eligible to be listed in this report section.
- Literature references cited in support of interpretive text are included in the report bibliography.

In light of the aforementioned, we have adopted a process for defining actionability based upon a more systematic methodology. We will perform a classification similar to what is being done in germline genetic tests. This classification will include the following three-part definitions:

A. Significance Classification:
   - Deleterious: Mutation has been studied before and is known to be tumorigenic.
   - Likely deleterious: Mutation is located in a known hotspot or functional domain.
   - Variant of unknown significance: Variant is located in an unknown region or domain.
   - Non-deleterious: Mutation is known to be non-tumorigenic.

B. Functionality Classification:
   - Loss of function (e.g., tumor suppressor gene).
   - Gain of function (e.g., oncogene).
   - Unknown

C. Actionability Classification:
   - Actionable: FDA-approved therapies exist in the patient's tumor type or another tumor type. Alternatively, a potential clinical trial exists at UCSD for which the patient may be a candidate based upon matched genomic alterations.

- Non-actionable: No FDA-approved therapies exist in the patient's tumor type or another tumor type. A potential clinical trial does not exist at UCSD for which the patient may be a candidate based upon matched genomic alterations.

*Note: regarding equivocal copy numbers per FoundationOne™ assay reports:

- Amplified: An alteration denoted as "amplification – equivocal" implies that FoundationOne ™ data provide some, but not unambiguous, evidence that the copy number of a gene exceeds the threshold for identifying copy number amplification. The threshold used in FoundationOne ™ for identifying a copy number amplification is five (5) for ERBB2 and six (6) for all other genes. Specific gene amplifications or amplified chromosomal segments meeting this threshold will be considered for actionability.
- Loss: An alteration denoted as "loss – equivocal" implies that FoundationOne data provide some, but not unambiguous, evidence for homozygous deletion of the gene in question.

**Note: regarding tumor heterogeneity

- Tumor heterogeneity is a biological limitation inherent to all treatments, whether matched or unmatched. Targetable mutations present in a minority of cells may not predict response in the same way that mutations in the dominant clone might. Sub-clonality will be considered on a case-by-case basis and combinations of matched therapies may be considered.

***Note: regarding mutations and copy number changes of genes

- We will distinguish between mutations and copy number changes of genes. We agree that amplifications and deletions can include large territories and the targets of these events reported by Foundation Medicine may not be the relevant drivers of the disease. However, drivers from passengers cannot be reliably distinguished at this point of time; therefore, this feasibility study will be based on making decisions that are to the "best of our knowledge."

****Note: regarding genomic alterations with unclear functional significance

- Foundation Medicine analyses may reveal genomic alterations with unclear functional significance. As previously discussed, we will rely on expert opinion and literature review of the Study Committee or Molecular Tumor Board rather than a preconceived algorithm to assess the functional impact of a mutation. This will include location of the mutation in a functional domain, the level of amplification, consistency with assumed tumor suppressor functions or oncogenic role of a given gene, or published functional assessment of the mutation. Those alterations considered to be non-deleterious will not be targeted. In line with our methodology, we will not employ a computationally predicted deleterious score because an FDA-approved device for such decision-making does not exist.

FDA-approved Companion Diagnostics

The current study will perform tumor molecular profiling through FoundationOne™ to navigate patients to a matched therapy. For those patients with an actionable tumor alteration identified by the FoundationOne™ assay, we will determine if an FDA-approved companion diagnostic test exists and is indicated for the patient based upon their malignancy (**Tables 1 and 2**). Appropriate companion diagnostic test(s) will be performed for the patient, along with any other clinically indicated standard of care laboratory tests (e.g., CEA for colorectal cancer and CA-19-9 for pancreas cancer). If there is a discrepancy between results of FoundationOne™ next generation sequencing and the FDA-approved test, the FDA-approved test results will supersede. For those patients with an actionable tumor alteration that is confirmed by a corresponding companion diagnostic test, matched therapy will be recommended by the Study Committee (or Molecular Tumor Board) from either FDA-approved drugs or matched agents under investigation in a separate clinical trial. Those patients, for whom there are no available matched treatments, will serve as the unmatched therapy controls. All final treatment decisions are the responsibility of the treating physician.

Table 1. Companion Diagnostic Tests: *In vitro* and Imaging Tools
(http://www.fda.gov/MedicalDevices/ProductsandMedicalProcedures/InVitroDiagnostics/ucm301431.htm; April 18, 2014**)**

Drug Trade Name (Generic Name)	NDA/BLA	Test Trade Name	PMA	Test Manufacturer	Intended Use (IU)/ Indications for Use (IFU)
Erbitux (cetuximab)	BLA 125084	*therascreen* KRAS RGQ PCR Kit	P110030	Qiagen Manchester, Ltd.	The *therascreen* KRAS RGQ PCR Kit is a real-time qualitative PCR assay used on the Rotor-Gene Q MDx instrument for the detection of seven somatic mutations in the human KRAS oncogene, using DNA extracted from formalin fixed paraffin-embedded (FFPE) colorectal cancer (CRC) tissue. The *therascreen* KRAS RGQ PCR Kit is intended to aid in the identification of CRC patients for treatment with Erbitux (cetuximab) based on a KRAS no mutation detected test result.
Erbitux (cetuximab) Vectibix (panitumumab)	BLA 125084; BLA 125147	DAKO EGFR PharmDx Kit	P030044 S001-S002	Dako North America, Inc.	The EGFR pharmDx™ assay is a qualitative immunohistochemical (IHC) kit system to identify epidermal growth factor receptor (EGFR) expression in normal and neoplastic tissues routinely-fixed for histological evaluation EGFR pharmDx specifically detects the EGFR (HER1) protein in EGFR-expressing cells.<br><br>EGFR pharmDx is indicated as an aid in identifying colorectal cancer patients eligible for treatment with Erbitux (cetuximab) or Vectibix (panitumumab).
Exjade (deferasirox)	NDA 021882	Ferriscan	K124065	Resonance Health Analysis Services Pty Ltd	The FerriScan R2-MRI Analysis System is intended to measure liver iron concentration to aid in the identification and monitoring of non-transfusion dependent thalassemia patients receiving therapy with deferasirox.
Gilotrif (afatinib)	NDA 201292	therascreen EGFR RGQ PCR Kit	P120022	Qiagen Manchester, Ltd.	The therascreen EGFR RGQ PCR Kit is a real-time PCR test for the qualitative detection of exon 19 deletions and exon 21 (L858R) substitution mutations of the epidermal growth factor receptor (EGFR) gene in DNA derived from formalin-fixed paraffin-embedded (FFPE) non-small cell lung cancer (NSCLC) tumor tissue. The test is intended to be used to select patients with NSCLC for whom GILOTRIF (afatinib), an EGFR tyrosine kinase inhibitor (TKI), is indicated. Safety and efficacy of GILOTRIF (afatinib) have not been established in patients whose tumors have L861Q, G719X, S768I, exon 20 insertions, and T790M mutations, which are also detected by the therascreen EGFR RGQ PCR Kit. Specimens are processed using the QIAamp DSP DNA FFPE Tissue Kit for manual sample preparation and the Rotor-Gene Q MDx instrument for automated amplification and detection.
Gleevec/Glivec (imatinib mesylate)	NDA 021335; NDA 021588	DAKO C-KIT PharmDx	P040011 S001-S002	Dako North America, Inc.	The c-Kit pharmDX assay is a qualitative immunohistochemical (IHC) kit system used on the Dako Autostainer, for the identification of c-kit protein/CD 117 antigen (c-kit protein) expression in normal and neoplastic formalin-fixed paraffin-embedded tissues for histological evaluation. The c-Kit pharmDX rabbit polyclonal antibodies specifically detect the c-kit protein in CD 117 antigen-expressing cells.<br><br>The c-Kit pharmDx is indicated as an aid in the differential diagnosis of gastrointestinal stromal tumors (GIST). After diagnosis of GIST, results from c-Kit pharmDx may be used as an aid in identifying those patients

Drug Trade Name (Generic Name)	NDA/BLA	Test Trade Name	PMA	Test Manufacturer	Intended Use (IU)/ Indications for Use (IFU)
					eligible for treatment with Gleevec/Glivec (imatinib mesylate). Results from hematoxylin and eosin (H&E) stains and a panel of antibodies can aid in the differential diagnosis of GIST. Interpretation must be made by a qualified pathologist, within the context of a patient's clinical history, proper controls, and other diagnostic tests.
Herceptin (trastuzumab)	BLA 103792	INFORM HER-2/NEU	P940004 S001	Ventana Medical Systems, Inc.	The Inform Her-2/Neu gene detection system is a fluorescence in situ hybridization (FISH) DNA probe assay that determines the qualitative presence of Her-2/Neu gene amplification on formalin-fixed, paraffin embedded human breast tissue as an aid to stratify breast cancer patients according to risk for recurrence or disease-related death. It is indicated for use as an adjunct to existing clinical and pathologic information currently used as prognostic indicators in the risk stratification of breast cancer in patients who have had a priori invasive, localized breast carcinoma and who are lymph node-negative.
Herceptin (trastuzumab)	BLA 103792	PATHVYSION HER-2 DNA Probe Kit	P980024 S001-S010	Abbott Molecular Inc.	The PathVysion HER-2 DNA Probe Kit (PathVysion Kit) is designed to detect amplification of the HER-2/neu gene via fluorescence in situ hybridization (FISH) in formalin-fixed, paraffin-embedded human breast cancer tissue specimens. Results from the PathVysion Kit are intended for use as an adjunct to existing clinical and pathologic information currently used as prognostic factors in stage II, node-positive breast cancer patients. The PathVysion Kit is further indicated as an aid to predict disease-free and overall survival in patients with stage II, node positive breast cancer treated with adjuvant cyclophosphamide, doxorubicin, and 5-fluorouracil (CAF) chemotherapy. The Pathvysion Kit is indicated as an aid in the assessment of patients for whom herceptin (trastuzumab) treatment is being considered (see herceptin package insert).
Herceptin (trastuzumab)	BLA 103792	PATHWAY ANTI-HER-2/NEU (4B5) Rabbit Monoclonal Primary Antibody	P990081 S001-S016	Ventana Medical Systems, Inc.	Ventana Medical Systems' PATHWAY Her2 (clone CB11) is a mouse monoclonal antibody intended for laboratory use for the semi-quantitative detection of c-erbB-2 antigen in sections of formalin-fixed, paraffin-embedded normal and neoplastic tissue on a Ventana automated immunohistochemistry slide staining device. It is indicated as an aid in the assessment of breast cancer patients for whom Herceptin treatment is being considered.
Herceptin (trastuzumab)	BLA 103792	INSITE HER-2/NEU KIT	P040030	Biogenex Laboratories, Inc.	InSite Her-2/neu Mouse Monoclonal Antibody (Clone C1B11) kit is intended for In Vitro Diagnostic use in Immunohistochemistry (IHC) assays to semi-quantitatively localize by light microscopy the over-expression of Her-2/neu (i.e., c-erbB-2) in formalin-fixed, paraffin-embedded normal and neoplastic tissue sections. InSite Her-2/neu is indicated as an aid in the assessment of breast cancer patients for whom Herceptin (Trastuzumab) therapy is being considered. Clinical interpretation of InSite Her-2/neu immunostaining results (absence or presence; semi-quantitative intensity score) should be complemented by appropriate controls and morphological tissue analysis and be evaluated by a qualified pathologist within the context of patient clinical history and other diagnostic results.

Drug Trade Name (Generic Name)	NDA/BLA	Test Trade Name	PMA	Test Manufacturer	Intended Use (IU)/ Indications for Use (IFU)
Herceptin (trastuzumab)	BLA 103792	SPOT-LIGHT HER2 CISH Kit	P050040 S001-S003	Life Technologies, Inc.	For In Vitro Diagnostic Use:<br>The SPOT-Light HER2 CISH Kit is intended to quantitatively determine HER2 gene amplification in formalin-fixed, paraffin-embedded (FFPE) breast carcinoma tissue sections using Chromogenic In Situ Hybridization (CISH) and brightfield microscopy.<br><br>This test should be performed in a histopathology laboratory.<br><br>The SPOT-Light HER2 CISH Kit is indicated as an aid in the assessment of patients for whom Herceptin (trastuzumab) treatment is being considered. The assay results are intended for use as an adjunct to the clinicopathological information currently being used as part of the management of breast cancer patients. Interpretation of test results must be made within the context of the patient's clinical history by a qualified pathologist.
Herceptin (trastuzumab)	BLA 103792	Bond Oracle Her2 IHC System	P090015	Leica Biosystems	The Bond Oracle Her2 IHC system is a semi-quantitative immunohistochemical (IHC) assay to determine Her2 (human epidermal growth factor receptor 2) oncoprotein status in formalin-fixed, paraffin-embedded breast cancer tissue processed for histological evaluation following automated staining on the bond-max slide staining instrument. The Bond Oracle Her2 IHC system is indicated as an aid in the assessment of patients for whom herceptin (trastuzumab) treatment is being considered.
Herceptin (trastuzumab)	BLA 103792	HER2 CISH PharmDx Kit	P100024 S001-S004	Dako Denmark A/S	HER2 CISH PharmDx kit is intended for dual-color chromogenic visualization of signals achieved with directly labeled in situ hybridization probes targeting the HER2 gene and centromeric region of chromosome 17. The kit is designed to quantitatively determine HER2 gene status in formalin-fixed, paraffin-embedded breast cancer tissue specimens. Red and blue chromogenic signals are generated on the same tissue section for evaluation under bright field microscopy. The CISH procedure is automated using Dako Autostainer instruments.<br><br>HER2 CISH pharmDx Kit is indicated as an aid in the assessment of patients for whom Herceptin (trastuzumab) treatment is being considered. Results from the HER2 CISH pharmDx Kit are intended for use as an adjunct to the clinicopathologic information currently used for estimating prognosis in stage II, node-positive breast cancer patients.<br><br>This kit is for in vitro diagnostic (IVD) use only.
Herceptin (trastuzumab)	BLA 103792	INFORM HER2 DUAL ISH DNA Probe Cocktail	P100027 S001-S006	Ventana Medical Systems, Inc.	The INFORM HER2 Dual ISH DNA Probe Cocktail is intended for use in determining HER2 gene status by enumeration of the ratio of the HER2 gene to Chromosome 17. The HER2 and Chromosome 17 probes are detected using two color chromogenic in situ hybridization (ISH) in formalin-fixed, paraffin-embedded human breast cancer tissue specimens following staining on Ventana BenchMark XT automated slide stainers (using NexES software), by light microscopy. The INFORM HER2 Dual ISH DNA Probe Cocktail is indicated as an aid in the assessment of

Drug Trade Name (Generic Name)	NDA/BLA	Test Trade Name	PMA	Test Manufacturer	Intended Use (IU)/ Indications for Use (IFU)
					patients for whom Herceptin (trastuzumab) treatment is being considered.<br><br>This product should be interpreted by a qualified reader in conjunction with histological examination, relevant clinical information, and proper controls.<br><br>This reagent is intended for in vitro diagnostic (IVD) use.
Herceptin (trastuzumab); Perjeta (pertuzumab); Kadcyla (ado-trastuzumab emtansine)	BLA 103792; BLA 125409	HERCEPTEST	P980018 S001-S017	Dako Denmark A/S	For in vitro diagnostic use.<br><br>HercepTest is a semi-quantitative immunocytochemical assay to determine HER2 protein overexpression in breast cancer tissues routinely processed for histological evaluation and formalin-fixed, paraffin-embedded cancer tissue from patients with metastatic gastric or gastroesophageal junction adenocarcinoma. HercepTest is indicated as an aid in the assessment of breast and gastric cancer patients for whom Herceptin (trastuzumab) treatment is being considered and for breast cancer patients for whom PERJETA (pertuzumab) treatment or KADCYLA (ado-trastuzumab emtansine) treatment is being considered (see Herceptin, PERJETA and KADCYLA package inserts).<br><br>**NOTE for breast cancer only:** All of the patients in the Herceptin clinical trials were selected using an investigational immunocytochemical clinical trial assay (CTA). None of the patients in those trials were selected using the HercepTest. The HercepTest was compared to the CTA on an independent set of samples and found to provide acceptably concordant results. The actual correlation of the HercepTest to Herceptin clinical outcome has not been established.<br><br>**NOTE for gastric cancer only:** All of the patients in the phase III BO18255 (ToGA) study sponsored by Hoffmann-La Roche were selected using Dako HercepTest (IHC) and Dako HER2 FISH pharmDx™ Kit (FISH). However, enrollment in the BO18255 study was limited to patients whose tumors were HER2 protein overexpressing (IHC 3+) or gene amplified (FISH+; HER2/CEN-17 ratio ≥ 2.0). No patients were enrolled whose tumors were not gene amplified but HER2 protein weakly to strongly overexpressing [FISH(-)/IHC 2+], therefore it is unclear if patients whose tumors are not gene amplified but HER2 protein overexpressing [i.e., FISH(-), IHC 2+ or 3+] will benefit from Herceptin treatment. The study also demonstrated that gene amplification and protein overexpression (IHC) are not as correlated as with breast cancer, therefore a single method should not be used to determine HER2 status.
Herceptin (trastuzumab); Perjeta (pertuzumab); Kadcyla (ado-	BLA 103792; BLA 125409	HER2 FISH PharmDx Kit	P040005 S001-S009	Dako Denmark A/S	HER2 IQFISH pharmDx is a direct fluorescence in situ hybridization (FISH) assay designed to quantitatively determine HER2 gene amplification in formalin-fixed, paraffin-embedded (FFPE) breast cancer tissue specimens and FFPE specimens from patients with metastatic gastric or gastroesophageal junction adenocarcinoma.

Drug Trade Name (Generic Name)	NDA/BLA	Test Trade Name	PMA	Test Manufacturer	Intended Use (IU)/ Indications for Use (IFU)
trastuzumab emtansine)					HER2 IQFISH pharmDx is indicated as an aid in the assessment of breast and gastric cancer patients for whom Herceptin (trastuzumab) treatment is being considered and for breast cancer patients for whom Perjeta (pertuzumab) or Kadcyla (ado-trastuzumab emtansine) treatment is being considered (see Herceptin, Perjeta and Kadcyla package inserts).<br><br>For breast cancer patients, results from the HER2 IQFISH pharmDx are intended for use as an adjunct to the clinicopathologic information currently used for estimating prognosis in stage II, node-positive breast cancer patients.
Mekinist (tramatenib) Tafinlar (dabrafenib)	NDA 204114; NDA 202806	THxID™ BRAF Kit	P120014	bioMérieux Inc.	The THxID BRAF kit is an In Vitro Diagnostic device intended for the qualitative detection of the BRAF V600E and V600K mutations in DNA samples extracted from formalin-fixed paraffin-embedded (FFPE) human melanoma tissue. The THxID™ BRAF kit is a real-time PCR test on the ABI 7500 Fast Dx system and is intended to be used as an aid in selecting melanoma patients whose tumors carry the BRAF V600E mutation for treatment with dabrafenib [Tafinlar] and as an aid in selecting melanoma patients whose tumors carry the BRAF V600E or V600K mutation for treatment with trametinib [Mekinist].
Tarceva (erlotinib)	NDA 021743	cobas EGFR Mutation Test	P120019	Roche Molecular Systems, Inc.	The cobas® EGFR Mutation Test is a real-time PCR test for the qualitative detection of exon 19 deletions and exon 21 (L858R) substitution mutations of the epidermal growth factor receptor (EGFR) gene in DNA derived from formalin-fixed paraffin-embedded (FFPET) human non-small cell lung cancer (NSCLC) tumor tissue. The test is intended to be used as an aid in selecting patients with metastatic NSCLC for whom Tarceva® (erlotinib), an EGFR tyrosine kinase inhibitor (TKI), is indicated.
Xalkori (crizotinib)	NDA 202570	VYSIS ALK Break Apart FISH Probe Kit	P110012 S001-S003	Abbott Molecular Inc.	The Vysis ALK Break Apart FISH Probe Kit is a qualitative test to detect rearrangements involving the ALK gene via fluorescence in situ hybridization (FISH) in formalin-fixed, paraffin-embedded (FFPE) non-small cell lung cancer (NSCLC) tissue specimens to aid in identifying patients eligible for treatment with Xalkori (crizotinib). This is for prescription use only.
Zelboraf (vemurafenib)	NDA 202429	COBAS 4800 BRAF V600 Mutation Test	P110020 S001-S006	Roche Molecular Systems, Inc.	The Cobas 4800 BRAF V600 Mutation Test is an in vitro diagnostic device intended for the qualitative detection of the BRAF V600E mutation in DNA extracted from formalin-fixed, paraffin-embedded human melanoma tissue. The Cobas 4800 BRAF V600 Mutation Test is a real-time PCR test on the Cobas 4800 system, and is intended to be used as an aid in selecting melanoma patients whose tumors carry the BRAF V600E mutation for treatment with vemurafenib.

Table 2. Nucleic Acid Based Tests
(http://www.fda.gov/MedicalDevices/ProductsandMedicalProcedures/InVitroDiagnostics/ucm330711.htm#human; April 18, 2014)

Disease	Trade Name	Manufacturer	Submission
Acute Myeloid Leukemia	Vysis D7S486/CEP 7 FISH Probe Kit	Abbott Molecular Inc.	K131508
	Vysis EGR1 FISH Probe Kit	Abbott Molecular Inc.	K123951, K091960
B-cell chronic lymphocytic leukemia	Vysis CLL FISH Probe Kit	Vysis	K100015
	CEP 12 SpectrumOrange Direct Labeled Chromosome Enumeration DNA Probe	Vysis	K962873
Bladder Cancer	Vysis UroVysion Bladder Cancer Recurrence Kit	Vysis	K033982, K013785, K011031
Breast Cancer	Prosigna Breast Cancer Prognostic Gene Signature Assay	Nanostring Technologies	K130010
	MammaPrint	Agendia BV	K101454, K081092, K080252, K070675, K062694
	GeneSearch Breast Lymph Node (BLN) Test Kit	Veridex, LLC.	P060017 S001-S004
	Dako TOP2A FISH PharmDx Kit	Dako Denmark A/S	P050045 S001-S004
Prostate Cancer	NADiA ProsVue	Iris Molecular Diagnostics	K101185
	PROGENSA PCA3 Assay	Gen-Probe, Inc.	P100033
Tissue of Origin	Pathwork Tissue of Origin Test Kit – FFPE	Pathwork Diagnostics Inc.	K120489, K092967
	Pathwork Tissue of Origin Test	Pathwork Diagnostics Inc.	K080896

Molecular Matching and Molecular Tumor Board/Study Committee
FoundationOne™ results of patients who have genomic alterations detected will be provided to the Molecular Tumor Board, which consists of the study principal investigators, co-investigators, the treating medical oncologist, other medical oncologists, surgical oncologists, radiologist, pathologist, other physicians, scientist specializing in genomics, a genetic counselor, and basic/translational scientists.[9] Since timeliness is essential for managing patients enrolled in this study, a Study Committee, that includes, but is not limited to, the study principal investigators and the treating medical oncologist may provide recommendations on an as needed basis between Molecular Tumor Board meetings in order to avoid delaying treatment. Using the aforementioned molecular aberrations, which represent druggable targets, either through direct pathway inhibition or through an associated pathway (via "crosstalk"), the "molecular individuality" of these tumors will be approached via a customized strategy.[9] Using an "N-of-One" model of precision medicine, the independent Study Committee will make a recommendation for a matched therapy that consists of at least one drug that targets a molecular alteration. Actionability will be limited by our ability to obtain drug(s). We expect 50-70% of patients will have potentially actionable alterations. This percentage is increasing rapidly as we learn more about the pathways and the drugs. We anticipate approximately 40% of patients with actionable alterations will be able to receive molecularly targeted "matched" treatment. These patients will be considered ARM A in each group. Expert opinion, rather than a preconceived algorithm will determine a molecularly matched therapy because a preconceived algorithm [6, 10] will likely be out of date by the time the trial ends. We realize there are downsides to "expert" opinion. However, as a feasibility trial,

we feel that it is a reasonable way to address this issue, and mimics what is done in the community.

Patients for whom we are unable to procure a matched treatment will be considered ARM B in each group. ARM B patients will receive treating physician's choice of traditional systemic chemotherapy treatment for their malignancy, defined by National Comprehensive Cancer Network (NCCN) guidelines and/or tumor board recommendation(s). If there is no standard of care treatment, the treating physician may select a regimen based upon published reports, series, and/or clinical trials.

Patients with no actionable alterations by FoundationOne***TM*** will receive treating physician's choice of traditional systemic chemotherapy treatment for their malignancy, defined by National Comprehensive Cancer Network (NCCN) guidelines and/or tumor board recommendation(s). If there is no standard of care treatment, the treating physician may select a regimen based upon published reports, series, and/or clinical trials.

Treatment Decisions
The Study Committee or Molecular Tumor Board will give suggestions but ultimately the treating physician chooses the treatment, whether matched or unmatched. While flexible choice is not a feature of many protocols, it was a feature of PREDICT and is a feature of WINther.[6] This has the advantage that the flexibility allows the treating physician to make the best informed choice for their patient, mimicking what would happen in the community, as well as making the trial logistically possible. This protocol is flexible and allows for the treating physician to make the best-informed choice for their patient. If a patient has a matched abnormality that suggests that they should receive a targeted agent that is different than an unmatched therapy offered in a clinical trial, the treating physician will have the final decision and not the study PIs. However, this is likely the case only in the previously treated patients because most treatment naïve patients will not be candidates for most clinical trials. **It is our intent to recommend potential therapy based upon the following Levels of Evidence as is being utilized by the NCI-MATCH Trial:**

- Level 1: FDA approved; evidence of target inhibition, or proof of mechanism; demonstration that patient selection with companion diagnostic is more likely to respond.
- Level 2: Agent met a clinical endpoint (objective response, PFS, or OS); with evidence of target inhibition; plausible evidence of a predictive or selection assay/analyte.
- Level 3: Agent demonstrated evidence of clinical activity with evidence of target inhibition; some evidence of a predictive or selection assay/analyte.
- Level 4: Preclinical evidence of anti-tumor activity and evidence of target inhibition; hypothesis for a predictive or selective assay/analyte.

In recommending therapies, treatments may include the following:
- FDA-approved single agents or investigational single agents in umbrella or basket trials.
- Two agents may be utilized if there is data published in peer-reviewed journals or abstract form demonstrating safety and/or efficacy of a two-drug combination.

But, the treating physician will choose based on his/her knowledge of the medical situation, the test results, and molecular tumor board recommendations. Therefore, all final treatment decisions are the responsibility of the treating physician. This feasibility study will determine whether molecular aberrations, which represent druggable targets, will be offered by treating oncologists to treatment naïve patients with incurable malignancies in order for patients to receive molecularly targeted matched treatment based on genomic analysis.

Evaluation of Treatment Decisions
Separately, the independent Study Committee will evaluate the degree of matching that occurred using the best information available at the end of the trial. They will be blinded to individual patient outcomes. However, this committee will also rely upon expert opinion rather than a preconceived algorithm, which would likely be out of date by the time the trial ends.

Insurance Coverage
A medication acquisition specialist will liaise and negotiate with insurance carriers to obtain authorization to prescribe matched regimens. If we cannot obtain insurance coverage for the matched therapy, the patient will receive their physician's choice of traditional systemic chemotherapy. **We will make all efforts to secure matched therapy, but this cannot be promised. If an insurance carrier denies the matched therapy, the patient will still receive their physician's choice of traditional systemic chemotherapy.**

Summary
Treatment will be selected and performed by the patient's treating physician and will not fall under the auspices of this study protocol. Drugs will be administered according to the guidelines found in the product label and the treating physician will account for special precautions or warnings relevant for each drug administered (e.g., incompatibility of agent with commonly used intravenous solutions, necessity of administering agent with food, pre-medications, hydration, whether any monitoring of vital signs during or shortly after treatment is required, etc.). Patients treated as part of clinical interventional clinical trials will be consented and treated according to the clinical study protocol. Regarding the recommended therapy regimen, the Study Committee, blinded to outcomes, will assess whether or not matching occurred. This determination will indicate whether the patient is Arm A or B.

Study Group 1 and Group 2 (treatment naïve)
ARM A:

A matched treatment regimen will be administered for approximately 8 weeks as dictated by recommendations for approved anti-cancer agents or as dictated by clinical trials. Disease-appropriate imaging (i.e., PET-CT vs. CT vs. MR imaging) will be performed at approximately week 8 according to standard of care or required clinical trial practices. Objective tumor response will determined according to RECIST v1.1 and this <u>First Response</u> assessment will guide subsequent treatment according to the following:

3. Patients demonstrating a PR or CR per RECIST 1.1 will continue treatment until
    - a complete response persists for at least six months
    - the tumor becomes resectable
    - their tumor response declines to SD or PD
    - they experience unacceptable toxicity.

4. Patients demonstrating SD or PD per RECIST 1.1 will be removed from matched therapy and placed onto the treating physician's choice of systemic chemotherapy appropriate to their disease. Alternatively, the treating physician may continue matched therapy at their discretion if there are no effective traditional systemic chemotherapy regimens available.

Patients who experience SD but short of PR while on matched treatment may, after experiencing progressive disease while receiving treating physician's choice of systemic chemotherapy appropriate to their disease, return to their prior matched treatment and will be monitored again for benefit. The degree of objective tumor response observed at the patient's First Response Assessment on the original matched treatment, will then guide subsequent treatment according to the following:

1. Patients demonstrating a 10-29% decrease in the sum of diameters of target lesions (First Response Assessment) will resume treatment with the original matched treatment regimen. Treatment will continue until:
    - a complete response persists for at least 6 months,
    - their tumor becomes resectable,
    - they experience progressive disease (PD), or
    - they experience unacceptable toxicity.
2. Patients demonstrating a <10% decrease in the sum of diameters of target lesions (First Response Assessment) will then receive the traditional second-line standard of care treatment appropriate for their disease.

ARM B:

Patients will receive treating physician's choice of traditional systemic chemotherapy for their malignancy, defined by National Comprehensive Cancer Network (NCCN) guidelines and/or tumor board recommendation(s). If there is no standard of care treatment, the treating physician may select a regimen based upon published reports, series, and/or clinical trials

A treatment regimen will be administered for approximately 8 weeks as dictated by recommendations for approved anti-cancer agents or as dictated by clinical trials. Disease-appropriate imaging (i.e., PET-CT vs. CT vs. MR imaging) will be performed at approximately week 8 according to standard of care or required clinical trial practices.

Study Group 3
ARM A:

A treatment regimen will be administered for approximately 8 weeks as dictated by recommendations for approved anti-cancer agents or as dictated by clinical trials. Disease-appropriate imaging (i.e., PET-CT vs. CT vs. MR imaging) will be performed at approximately week 8 according to standard of care or required clinical trial practices. Objective tumor response will determined according to RECIST v1.1 and this <u>First Response</u> assessment will guide subsequent treatment according to the following:

2. Patients demonstrating a PR or CR per RECIST 1.1 will continue treatment until
    - a complete response persists for at least six months
    - the tumor becomes resectable
    - their tumor response declines to SD or PD
    - they experience unacceptable toxicity.

ARM B:

Patients will receive treating physician's choice of traditional systemic chemotherapy for their malignancy, defined by National Comprehensive Cancer Network (NCCN) guidelines and/or tumor board recommendation(s). If there is no standard of care treatment, the treating physician may select a regimen based upon published reports, series, and/or clinical trials.

A treatment regimen will be administered for approximately 8 weeks as dictated by recommendations for approved anti-cancer agents or as dictated by clinical trials. Disease-appropriate imaging (i.e., PET-CT vs. CT vs. MR imaging) will be performed at approximately week 8 according to standard of care or required clinical trial practices.

Dosing Delays/Dose Modifications
Dose modifications will be determined by the treating physician appropriate for the particular agent administered. Investigational agents administered on clinical trials will have specific dose modifications provided in that trial. FDA approved drugs will be dosed in accordance with the package insert. Toxicities, dose reductions and monitoring will follow protocol for investigational drugs and package inserts for approved drugs. All toxicities grade 3 and above will be tabulated for FDA approved drugs. For investigational drugs on protocol, toxicities will be tabulated per protocol.

Permitted concomitant therapy
Supportive care and medical management drugs/treatments are allowed as deemed appropriate by the treating medical oncologist and consistent with treatment guidelines and clinical trials.

Prohibited concomitant therapy
Investigational-targeted therapies may be used while the patient is on the study if they are the appropriate matched therapy for the patient's disease. If additional un-matched anti-cancer agents are administered in combination with matched therapy, then the patient will no longer be considered to be on matched therapy unless the patient already received that unmatched therapy and did not achieve a PR or CR.

Other Modalities or Procedures
Modalities (e.g., surgery, radiotherapy) or procedures (e.g., tumor biopsy by a surgeon, interventional radiologist/gastroenterologist/pulmonologist) will be standard of care. No additional modalities or procedures will be offered within the protocol.

Duration of Follow Up
Patients will be followed every 3 months (±1 month) until death for survival contacts.

Patient Replacement
Patients may be replaced if they have withdrawn from the study prior to having their tumor profiled in order to obtain 1150 evaluable patients with FoundationOne^{TM} analyses.

4.0 STUDY PROCEDURES

Screening/Baseline Procedures

Assessments performed exclusively to determine eligibility for this study will be done only after obtaining informed consent. Assessments performed for clinical indications (not exclusively to determine study eligibility) may be used for baseline values even if the studies were done before informed consent was obtained.

Informed Consent

Subjects will be asked to provide written consent using an IRB-approved consent form. The investigator or study coordinator will describe the study, including detailed information about risks, benefits, and voluntary nature of the study, to potential subjects. Subjects will be given ample time to read the consent form at the same visit or may take it with them to read at another time. Potential study subjects will be given the opportunity to ask any questions they may have about the study, including its risks and benefits, or about the consent form itself before signing the consent form. As this research is subject to HIPAA privacy rule provisions, participants will also be requested to sign a separate authorization for the use of protected health information (i.e., HIPAA form specific to the research study).

The investigator or study coordinator will obtain informed consent in a language understood by the prospective participant or their legally authorized representative, using certified translations of study documents and qualified translators, where applicable.

Patients who fulfill the eligibility criteria will be offered further participation in this study. Only patients who have consented and provided HIPAA authorization will have identifiers or linked information (e.g., patients initials, study numbers, etc.) recorded on the Screening/Enrollment Log.

Medical history

Complete medical, surgical and oncology history as well as history of infections are obtained at screening.

Demographics

Demographic profile will include date of birth, gender, race, and ethnicity.

Review subject eligibility criteria

Review of eligibility criteria to ensure subject qualification for study entry.

Performance status

ECOG performance status evaluated prior to study entry.

New York Heart Association Classification

Classification evaluated prior to study entry.

Hematology

As dictated by standard of care or a clinical trial.

Serum chemistries

As dictated by standard of care or a clinical trial, including pregnancy test for women of child bearing potential.

Assessments for an individual protocol or drug

Assessments specific for an individual treatment protocol or drug will be performed as indicated by that protocol. Examples may include additional blood work and an echocardiogram. Patients must meet eligibility criteria for enrollment on any other given trial.

Tumor assessment

Tumor imaging (or clinical exam when applicable) will be performed as part of standard of care. Imaging based evaluation should always be done rather than clinical examination unless the lesion(s) being followed cannot be imaged but are assessable by clinical exam. The same method of assessment and the same technique should be used to characterize each identified and reported lesion at baseline and during follow-up.

Patients will be considered evaluable for the study if they had their baseline scan within 30 days of the start of therapy. Tumor measurements will be assessed according to RECIST v1.1 (see Appendix A).

In addition, biochemical markers (tumor markers) may be considered as a secondary method for assessing tumor response.

Tumor markers (for diseases included, but not limited to)

Ampullary carcinoma	CEA
Appendiceal cancer	CEA
Breast cancer	CA15-3
Colorectal cancer (CRC)	CEA
Esophageal adenocarcinoma	CEA
Extrahepatic cholangiocarcinoma (EHCC)	CEA, CA-19-9
Gallbladder cancer (GBCA)	CEA, CA-19-9
Gastric adenocarcinoma	CEA
Hepatocellular carcinoma (HCC)	AFP, DCP
Intrahepatic cholangiocarcinoma (IHCC)	CEA, CA-19-9
Ovarian cancer	CA-125
Pancreatic ductal adenocarcinoma (PDAC)	CA-19-9
Prostate cancer	PSA
Small bowel adenocarcinoma (including duodenal)	CEA

Molecular Profiling

A sample of the subject's blood will be collected or the subject's tumor will be obtained via biopsy, surgical resection, or fine needle aspiration during screening and prior to study treatment. For new tumor samples procured at UCSD, tissue will be placed in formalin and sent to the UCSD Department of Pathology for clinical care. For new tumor samples procured at Avera McKennan, tissue will be placed in formalin and sent to the Avera McKennan Department of Pathology for clinical care. The tissue will be formalin-fixed paraffin-embedded (FFPE). All clinical diagnoses will be performed. For subject's tumor samples previously biopsied at UCSD or outside institutions, pathological re-examination and diagnosis confirmation by the UCSD Department of Pathology faculty will be performed at the discretion of the treating physician. . For Avera McKennan's subject's tumor samples previously biopsied at Avera McKennan or outside institutions, pathological re-examination and diagnosis confirmation by the Avera McKennan Department of Pathology faculty will be performed at the discretion of the treating physician. For tumor samples procured from outside institutions, a slide case request will be performed prior to receipt of tissue. A pathologist will then review the FFPE blocks as described below. Subsequently, 8-10 unstained FFPE slides plus 1 H&E slide as needed will be sent to Foundation Medicine for FoundationOne™ analysis.

Alternatively, for tumor samples (FFPE slides or blocks) procured from outside institutions where pathological re-review is not deemed to be clinically necessary according to the treating physician, FFPE blocks or about 8-10 unstained FFPE slides plus 1 H&E slide as needed will be directly sent to Foundation Medicine for FoundationOne™ analysis.

Tissue/blood samples will be shipped ambient by priority overnight FedEx to the following address:

John Curran
Foundation Medicine, Inc.
150 Second Street, 1st Floor
Cambridge, MA 02141

No additional tissue/blood will be retained for this protocol.

Dr. Donna Hansel, Director of Anatomic Pathology, or her designee, will be responsible for reviewing and approving requests for clinical specimen to be sent to Foundation Medicine, a CLIA certified laboratory. Any data obtained from the use of clinical specimen will be the property of UCSD for publication and any licensing agreement will be strictly adhered to. Following patient consent and HIPAA compliance, annotated clinical information will be sent to Foundation Medicine. There will be secure storage of linking data kept in the form of paper and electronic files. These will be stored in the Clinical Trials Office. Results of genomic testing will also be uploaded to the patient's electronic medical record system and clinical trial management system. No future uses are envisioned.

Procedures during treatment
As dictated by standard of care or a clinical trial.
Tumor Assessments
To be done approximately every 8 weeks.

Follow-up Procedures
Patients will be followed every 3 months (± 1 month) until death. Follow-up will consist of survival contacts via medical record review, telephone call, or review of the Social Security Index.

Removal of Subjects from Study
Patients can be taken off the study at any time at their own request, or they may be withdrawn at the discretion of the investigator for behavioral or administrative reasons. The reason(s) for discontinuation will be documented and may include:

- **Patient withdraws consent;**
- **Patient is unable to comply with protocol requirements;**
- **Treating physician judges continuation on the study would not be in the patient's best interest.**

5.0 Measurement of Effect

Feasibility
This feasibility study is an evaluation and analysis of the potential of a proposed methodology that is based upon molecular profiling to support decision making in individualized cancer therapy for patients with incurable malignancies. Like other feasibility studies, we will objectively and rationally uncover the strengths and weaknesses of this approach in the academic environment of UCSD with our collaborators. This feasibility study will evaluate this methodology's potential for success. We termed this a feasibility study because genomically driven trials, especially in this setting, are relatively new. As such, we might encounter logistical hurdles that will need to be addressed and overcome before a definitive trial is performed.

For the primary objective, we consider matching therapy for ≥40% of patients (N≥30) in each Group to define a successful feasibility endpoint. Additional measure of feasibility will be the ability to enroll patients in each Group, the turnaround time for genomic analysis, and the ability to identify actionable genomic alterations.

Tumor Response
Response and progression will be evaluated in this study using the international criteria proposed by the Response Evaluation Criteria in Solid Tumors (RECIST) Committee [*JNCI* 92(3):205-216, 2000]. Changes in only the largest diameter (one-dimensional measurement) of the tumor lesions are used in the RECIST v1.1.

Best Overall Response
Best response is the maximal response recorded from the start of a treatment until the end of treatment taking into account any requirement for confirmation.

Progression-Free Survival
Progression-free survival (PFS) is defined as the duration of time from the start of a treatment until objective tumor progression or death.

Overall Survival
Overall survival (OS) is defined as the duration of time from the start of treatment to death.

Change in Resectability
For those patients in Group 1 who are classified as having unresectable disease (as opposed to being medically unfit), their disease will be assessed throughout the study to determine if

treatment leads to their disease becoming resectable as deemed by two surgeons. Resectability is based upon tumor location, vascular involvement, and technical feasibility issues.

Adverse Events

Adverse events will be evaluated by the NCI Common Terminology Criteria for Adverse Events CTCAE version 4.03 (http://ctep.cancer.gov/reporting/ctc.html) to determine the incidence of serious adverse events, as defined below.

5.4.1 Adverse Event Monitoring

Information about all adverse events, whether volunteered by the subject, discovered by investigator questioning, or detected through physical examination, laboratory test or other means, will be collected and followed as appropriate by the patient's treating physician.

This study will document all serious adverse events (as defined below in section 5.4.2 – Definitions) experienced by a subject while they are participating on the study and for 4 weeks after discontinuation of the matched and/or unmatched regimens chosen by their treating physician based on study navigation, or the start of a new therapy (whichever comes first).

5.4.2. Definitions

Adverse Event

An adverse event (AE) is any undesirable sign, symptom or medical condition occurring after starting treatment even if the event is not considered to be related to the treatment.

Medical conditions/diseases present before starting treatment are only considered adverse events if they worsen after starting treatment.

Grade

All adverse events will be graded according to the NCI Common Terminology Criteria for Adverse Events (CTCAE) version 4.03. The CTCAE v4.03 is available at http://ctep.cancer.gov/reporting/ctc.html

If no CTCAE grading is available, the severity of an AE is graded as follows:

Mild (grade 1): the event causes discomfort without disruption of normal daily activities.
Moderate (grade 2): the event causes discomfort that affects normal daily activities.
Severe (grade 3): the event makes the patient unable to perform normal daily activities or significantly affects his/her clinical status.
Life-threatening (grade 4): the patient was at risk of death at the time of the event.
Fatal (grade 5): the event caused death.

Relationship

Attribution categories for adverse events in relationship to treatment are as follows:

Definite – The AE *is clearly related* to the study treatment.
Probable – The AE *is likely related* to the study treatment.
Possible – The AE *may be related* to the study treatment.

Unlikely – The AE *is doubtfully related* to the study treatment.
Unrelated – The AE *is clearly NOT related* to the study treatment.

Seriousness
A serious adverse event is an undesirable sign, symptom or medical condition which:

- is fatal or life-threatening
- required or prolonged hospitalization
- results in persistent or significant disability/incapacity
- constitutes a congenital anomaly or a birth defect
- is medically significant, may jeopardize the subject and may require medical or surgical intervention to prevent one of the outcomes listed above.

Events not considered to be serious adverse events are hospitalizations for the:

- routine treatment or monitoring of the studied indication not associated with any deterioration in condition.
- elective or pre-planned treatment for a pre-existing condition that did not worsen.
- treatment on an emergency, outpatient basis for an event not fulfilling any of the definitions of serious given above and not resulting in hospital admission.

5.4.3 Prior experience

Expected events are those that have been previously identified as resulting from administration of the agent. An adverse event is considered unexpected, for expedited reporting purposes only, when either the type of event or the severity of the event is not currently known.

5.4.4 Expedited Reporting

The Principal Investigator must be notified within 24 hours of learning of any serious adverse events, occurring during the study.

The UCSD Human Research Protections Program (HRPP) must be notified within 10 business days of "any unanticipated problems involving risk to subjects or others" (UPR).

Avera McKennan Cancer Institute Regulatory Department will be notified within 10 business days of "any unanticipated problems involving risk to subjects or others" and inform UCSD within the same time interval.

5.4.5. The following events meet the definition of UPR:

- Any serious event (injuries, side effects, deaths or other problems), which in the opinion of the Principal Investigator was unanticipated, involved risk to subjects or others, and was possibly related to the research procedures.
- Any serious accidental or unintentional change to the IRB-approved protocol that alters the level of risk.

- Any deviation from the protocol taken without prior IRB review to eliminate apparent immediate hazard to a research subject.
- Any new information (e.g., publication, safety monitoring report, updated sponsor safety report), interim result or other finding that indicates an unexpected change to the risk/benefit ratio for the research.
- Any breach in confidentiality that may involve risk to the subject or others.
- Any complaint of a subject that indicates an unanticipated risk or that cannot be resolved by the Principal Investigator.

5.4.6 Instructions for routine reporting of adverse events:

The UCSD HRPP will be notified of any adverse events that are not unanticipated problems involving risk to subjects or other (non-UPRs) at the time of the annual Continuing Review.

6 STATISTICAL CONSIDERATIONS

6.1 Study Design

This is a prospective, open label navigational investigation to evaluate the feasibility of using molecular profile-based evidence to determine individualized cancer therapy for patients with incurable malignancies. This is a non-randomized, histology-agnostic trial. Although there will be a case mix of histologies, we now know that individual histologies are composed of a heterogeneous mix of genomic alterations. It is not clear that one case mix is better or worse than another. Thus, we are testing a strategy of molecular matching that may apply across cancers. All eligible and consented patients will have their tumor tissues/blood genomic profiled by Foundation Medicine's FoundationOne™ genomic analysis. Patients will be stratified into Group 1 (treatment naïve, unresectable/medically unfit for surgery), Group 2 (treatment naïve, metastatic), and Group 3 (prior treated), respectively. Following analysis for genomic alterations, matched therapy, if available, will be recommended by the Study Committee or Molecular Tumor Board. If the patients received the matched therapy, they are designated as in Arm A. Otherwise, if the patients received the unmatched therapy (i.e., treating physician's choice of traditional systemic chemotherapy), they are designated as in Arm B. The study feasibility will be measured by the ability to enroll patients, the acceptable turnaround time and the actionable information obtained from the genomic profiling, and the viability of identifying and delivering the matched therapy. The treatment efficacy will be determined among the patients groups and treatment arms. The safety profile of the treatment will be assessed as well.

6.2 Sample Size and Accrual

This is a feasibility study with descriptive analysis as the primary analysis to characterize the study findings. We plan to enroll a total of 150 patients in Groups 1 and 2 and an unlimited number of patients in Group 3 (of up to 1000 patients) with their blood/tissues subject to the FoundationOne™ assay. We project to enroll patients in 30 months plus additional 6 months of follow-up. The total study period is 3 years. We expect that the accrual rate is 2 patients per month for Groups 1 and 2, and 4 patients per month for Group 3 in the first year. After the study mechanism is established and if we begin to see objective responses with matched therapy, we expect these rates to increase to 3 patients per months for Groups 1 and 2, and 5 patients per month for Group 3 in subsequent years. The total and percent of patients in Arm A and Arm B for each group will be determined to assess the feasibility of this study. With the comprehensive genomic profiling, we expect that 40% of the patients (N=30) will be in Arm A and 60% of the patients (N=45) will be in Arm B for all three patients groups.

6.3 Statistical Methods

This is a hypothesis-generating trial and not a hypothesis-driven trial. We will collect data prospectively by running concurrent trials (e.g., Groups and Arms) and describing outcomes. We will then analyze the data.

Therefore, the primary statistical analysis is descriptive in nature. Due to the moderate size of the study, the statistical analysis is primarily exploratory and descriptive rather than definitive. The information gathered in this exploratory study will be used for designing future confirmative studies. Summary statistics such as the frequency counts for the categorical variables and minimum, maximum, median, mean, and standard deviation for continuous variables will be computed. In addition, for the analyses of exploratory endpoints, Kaplan-Meier analyses will be computed to estimate the survival rate for time-to-event endpoints. Estimation with both point estimator and 95% confidence estimation for the study endpoints will be provided. Hypothesis testing with appropriate statistical methods will be performed as indicated. For example, Fisher's exact test and chi-square test will be applied to test equal proportion among groups for categorical data such as the response status. Two-sample t-test and Wilcoxon rank sum test will be used to assess the distribution of continuous variable such as the tumor regression percentage between two independent groups. Analysis of variance (ANOVA) and Friedman's test will be applied to assess the distribution of continuous variable among 3 or more groups. Log-rank test and Cox regression model will be applied for comparing the time-to-event endpoints between groups.

6.4 Sample Size Calculation

All exploratory analysis will test the hypothesis of greater response rate in Arm A than that in Arm B. With an estimated sample size of 30 (ARM A) and 45 (ARM B) in each group, we can estimate the response rate with a standard error no larger than 0.09 and 0.07, respectively. We will have sufficient statistical power to detect the difference between groups only if the difference between groups is sufficiently large. For example, with the sample size of 30 and 45 for Arm A and Arm B, we will have 79% power to detect the response rate of 0.25 and 0.05 between the two arms with one-sided 10% type I error rate using the continuity corrected chi-square test.

We will have more than 80% power to detect the difference between Arm A and Arm B using the log-rank test when the median PFS is 4 months and 2 months for the two arms, respectively.

In addition, in Group 1A, we will estimate the proportion of patients who are initially unresectable (as deemed by two surgeons) but converted to resectable (as deemed by two surgeons) after 8 weeks of the matched treatment, or if ever resected. With 30 patients, we expect to see at least one patient becomes resectable with a probability of 0.79, 0.96, and 0.99 if the conversion rate is 0.05, 0.10, and 0.15, respectively. We expect to see at least 2 patients become resectable with a probability of 0.82 and 0.95 if the conversion rate is 0.10 and 0.15, respectively. One-sided nominal level at 10% will be used to declare statistical significance. No P value adjustment for multiplicity will be applied due to the exploratory nature of the study. All study results need to be confirmed by future studies with larger sample sizes.

- The response rate (proportion of patients reaches CR or PR) will be estimated based on RECIST for each patient group and each treatment arm.
- The tumor regression rate (in %) will be calculated by the percent tumor size reduction from baseline to after treatment will be determined in and across patient groups.
- Progression-free survival (PFS) and overall survival (OS) will be determined in and across patient groups and treatment arms.
- The results of PFS and OS outcomes will be described relative to historical controls or published data. Due to the potential difference in patient populations between the current study and historical controls, we will also perform multivariate analyses to adjust the difference in prognostic factor(s) when comparing the PFS and OS between the different cohorts.
- The treatment grade 3-5 adverse events will be documented and analyzed by descriptive statistics.

As described earlier, this is a feasibility and descriptive study that will generate preliminary data for designing future studies. The exact numbers of patients in Arm A and Arm B for each group correspond to the feasibility endpoints. Although these numbers are unknown in the design stage, with the comprehensive genomic profiling and dedicated efforts in finding and securing the matched therapy, we expect that 40% of the patients (N=30) will be in Arm A and 60% of the patients (N=45) will be in Arm B for all three patients groups.

6.5 Planned Analyses

This primary statistical analysis is descriptive in nature. Summary statistics such as the frequency counts for the categorical variables and minimum, maximum, median, mean, and standard deviation for continuous variables will be computed. The Kaplan-Meier curve will be computed to estimate the survival rate for time-to-event endpoints. Estimation with both point estimator and 95% confidence estimation for the study endpoints will be provided. Hypothesis testing with appropriate statistical methods will be performed as indicated. For example, Fisher's exact test and chi-square test will be applied to test equal proportion among groups for categorical data such as the response status. Two-sample t-test and Wilcoxon rank sum test will be used to describe the distribution of continuous variable such as the tumor regression percentage between two independent groups. Analysis of variance (ANOVA) and Friedman's test will be applied to describe the distribution of continuous variable among 3 or more groups. Log-rank test and Cox regression model will be applied for comparing the time-to-event endpoints between groups. However, due to the moderate size of the study, the statistical analysis is primarily exploratory rather than definitive. The information gathered in this exploratory study will be used for designing future confirmative studies.

The primary and secondary are for feasibility and will be estimated based upon four parameters:

Primary Endpoint:

- To determine the proportion of patients who receive molecularly targeted matched treatment based on genomic analysis.

Secondary Endpoints:

- We will determine the proportion of patients who consent to enroll out of those approached for study participation.
- We will determine the time of consent to physician receipt of Foundation Medicine's FoundationOne™ genomic analysis.
- We will determine the proportion of patients with actionable alterations identified in the groups (Groups 1-3), and the entire study population

The point estimate and the 95% confidence interval estimation on the proportions will be computed. For the time to consent to receiving the genomic analysis, the distribution including the mean, standard deviation, minimum, 25th percentile, median, 75th percentile, and maximum will be computed. One dimensional distribution plot will be constructed to further characterize the time distribution.

6.6 Exploratory Analyses

Exploratory analyses will be conducted to evaluate efficacy and adverse event outcomes in each group and ARM and for comparison across groups.

All exploratory analysis will test the hypothesis of greater response rate in Arm A than that in Arm B. With an estimated sample size of 30 (ARM A) and 45 (ARM B) in each group, we can estimate the response rate with a standard error no larger than 0.09 and 0.07, respectively. We will have sufficient statistical power to detect the difference between groups only if the difference between groups is sufficiently large. For example, with the sample size of 30 and 45 for Arm A and Arm B, we will have 79% power to detect the response rate of 0.25 and 0.05 between the two arms with one-sided 10% type I error rate using the continuity corrected chi-square test.

We will have more than 80% power to detect the difference between Arm A and Arm B using the log-rank test when the median PFS is 4 months and 2 months for the two arms, respectively.

In addition, in Group 1A, we will estimate the proportion of patients who are initially unresectable but converted to resectable after 8 weeks of the matched treatment, or if ever resected. With 30 patients, we expect to see at least one patient becomes resectable with a probability of 0.79, 0.96, and 0.99 if the conversion rate is 0.05, 0.10, and 0.15, respectively. We expect to see at least 2 patients become resectable with a probability of 0.82 and 0.95 if the conversion rate is 0.10 and 0.15, respectively. One-sided nominal level at 10% will be used to declare statistical significance. No P value adjustment for multiplicity will be applied due to the exploratory nature of the study. All study results need to be confirmed by future studies with larger sample sizes.

- The response rate (proportion of patients reaches CR or PR) will be estimated based on RECIST for each patient group and each treatment arm.
- The tumor regression rate (in %) will be calculated by the percent tumor size reduction from baseline to after treatment will be determined in and across patient groups.
- Progression-free survival (PFS) and overall survival (OS) will be determined in and across patient groups and treatment arms.
- The results of PFS and OS outcomes will be described relative to historical controls or published data. Due to the potential difference in patient populations between the current study and historical controls, we will also perform multivariate analyses to adjust the

difference in prognostic factor(s) when comparing the PFS and OS between the different cohorts.
- The treatment grade 3-5 adverse events will be documented and analyzed by descriptive statistics.

Analysis Plan for Group 1 and Group 2

- The treatment efficacy in the matched cohorts (i.e., Groups 1A and 2A) and in the cohort of patients receiving treating physician's choice of traditional systemic chemotherapy [i.e., no drug(s) available for target(s), no insurance coverage for recommended drug(s) = Group 1B and 2B, respectively] will be described.
- For the unresectable patients, determine whether the resectability rate in Group 1A is higher than in Group 1B.

Analysis Plan for Group 3:

- For patients in 3A and 3B, the PFS of the last unmatched therapy (PFS1) and the PFS of the current therapy (PFS2) will be described. PFS1 will be collected retrospectively unless prospectively collected data is available. We will be limited in our ability to control for this. Thus, in order to avoid bias, we will have an independent physician assess the prior PFS. We will then estimate the percent of patients with PFS2/PFS1 > 1.5, and PFS2/PFS1 > 1.3.
- The treatment efficacy in the matched cohort (i.e., matched cohorts = 3A) and in the cohort of patients receiving treating physician's choice of traditional systemic chemotherapy [i.e., no drug(s) available for target(s), no insurance coverage for recommended drug(s) = Group 3B] will be described.

For PFS2/PFS1 ratio analyses, patients with matched therapy for PFS1 will be analyzed separately from patients with unmatched therapy for PFS1.

Analysis Plan for adverse events

Treatment adverse events will be evaluated by the NCI Common Terminology Criteria for Adverse Events CTCAE version 4.03 (http://ctep.cancer.gov/reporting/ctc.html). Analyses will be performed for all patients enrolling to the study that subsequently receive matched therapy or physician's choice of traditional systemic chemotherapy. Frequency tabulation for all toxicities Grades 3-5 will be generated by the following:

- **Incidence.**
- **Patient.**
- **Disease.**
- **Drug.**
- **Drug combination (Note: given the infinite number of potential drugs and combinations, we cannot ascertain this *a priori*).**

Safety Stopping Rule

Suspension of study enrollment will occur for ANY of the following overall (irrespective of patient group or treatment arm):

a. Greater than 10 drug related SAEs.
b. Greater than 10 drug related Grade 4-5 toxicities.
c. Simon's two-stage design (Simon, 1989) will be used. The null hypothesis that the true response rate is 0.05 will be tested against a one-sided alternative. In the first stage, 13 patients will be accrued. If there are 0 responses in these 13 patients, the study will be stopped. Otherwise, 14 additional patients will be

accrued for a total of 27. The null hypothesis will be rejected if 4 or more responses are observed in 27 patients. This design yields a type I error rate of 0.0416 and power of 0.8011 when the true response rate is 0.20. (http://www.cscc.unc.edu/cscc/aivanova/SimonsTwoStageDesign.aspx)

Simon's Two-Stage design

This program generates Simon's optimal two-stage designs (Simon, 1989) and admissible designs from Jung et al. (2004) for Phase II single arm clincial trials.

1. Simon R (1989). Controlled Clinical Trials 10: 1-10. Click here to download Simon's (1989) article.
2. Jung SH, Lee TY, Kim KM, George S (2004). Admissible two-stage designs for phase II cancer clinical trials, Statistics in Medicine 23: 561-569.

Type I error rate, α (one-sided): 0.05
Power: 0.8
Response probability of poor drug, p_0: 0.05
Response probability of good drug, p_1: 0.20
Calculate

n	n_1	r_1	r_2	Type 1 Error	Power	EN_0	Probability of early stopping	Interval for w	Comment
27	13	0	3	0.0416	0.8011	19.8	0.5133	[0.5973,1]	Minimax

n is the total number of subjects
n_1 is the number of subjects accrued during stage 1
r_1, if r_1 or fewer responses are observed during stage 1, the trial is stopped early for futility
r_2, if r_2 or fewer responses are observed by the end of stage two, then no further investigation of the drug is warranted
EN_0 is the expected sample size for the trial when response rate is p_0
Interval for w is the set of values w such that the design minimizes $w * n + (1 - w) * EN_0$

The above referenced protocol has been audited since 2016 by the UCSD Moores Cancer Center Data Safety and Monitoring Board (DSMB). The study is not interventional in the traditional sense and as the title states is a navigational investigation. Since patients receive multiple different therapies or no therapies at all, it is not feasible to track safety results due the multiple modalities used. It has been decided that the DSMB will no longer audit this study, as of January 6, 2020.

7 STUDY MANAGEMENT

7.1 Conflict of Interest

Any investigator who has a conflict of interest with this study (patent ownership, royalties, or financial gain greater than the minimum allowable by their institution, etc.) must have the conflict reviewed according to UCSD conflict of interest policy.

7.2 Institutional Review Board (IRB) Approval and Consent

The IRB should approve the consent form and protocol prior to any study-related activities. It is expected that the IRB will have the proper representation and function in accordance with federally mandated regulations.

Avera Cancer Institute is conducting this trial under the purview of their local IRB. UCSD is not the IRB of record for Avera Cancer Institute.

In obtaining and documenting informed consent, the investigator should comply with the applicable regulatory requirement(s), and should adhere to Good Clinical Practice (GCP) and to ethical principles that have their origin in the Declaration of Helsinki.

Before recruitment and enrollment onto this study, the patient will be given a full explanation of the study and will be given the opportunity to review the consent form. Each consent form must include all the relevant elements currently required by the FDA Regulations and local or state regulations. Once this essential information has been provided to the patient and the investigator is assured that the patient understands the implications of participating in the study, the patient will be asked to give consent to participate in the study by signing an IRB-approved consent form.

Prior to a patient’s participation in the trial, the written informed consent form should be signed and personally dated by the patient and by the person who conducted the informed consent discussion.

7.3 Subject Data Protection

In accordance with the Health Information Portability and Accountability Act (HIPAA), subjects who have provided written informed consent must also sign a subject authorization to release medical information to the study Sponsor and allow a regulatory authority, or Institutional Review Board access to subject’s medical information relevant to the study.

7.4 Data and Safety Monitoring

The above referenced protocol has been audited since 2016 by the UCSD Moores Cancer Center Data Safety and Monitoring Board (DSMB). The study is not interventional in the traditional sense and as the title states is a navigational investigation. Since patients receive multiple different therapies or no therapies at all, it is not feasible to track safety results due the multiple modalities used. It has been decided that the DSMB will no longer audit this study, as of January 6, 2020.

I

7.5 Record Retention

Study documentation includes all Case Report Forms, data correction forms or queries, source documents, Sponsor-Investigator correspondence, monitoring logs/letters, and regulatory documents (e.g., protocol and amendments, IRB correspondence and approval, signed patient consent forms).

Source documents include all recordings of observations or notations of clinical activities and all reports and records necessary for the evaluation and reconstruction of the clinical research study.

Government agency regulations and directives require that the study investigator must retain all study documentation pertaining to the conduct of a clinical trial. In the case of a study with a drug seeking regulatory approval and marketing, these documents shall be retained for at least two years after the last approval of marketing application in an International Conference on Harmonization (ICH) region. In all other cases, study documents should be kept on file until three years after the completion and final study report of this investigational study.

7.6 Obligations of Investigators

The Principal Investigators are responsible for the conduct of the clinical trial at the site in accordance with Title 21 of the Code of Federal Regulations and/or the Declaration of Helsinki. The Principal Investigators are not responsible for personally overseeing the treatment of all study patients. This is the responsibility of the treating physician. The Principal Investigator must assure that all study site personnel, including sub-investigators and other study staff members, adhere to the study protocol and all FDA/GCP/NCI regulations and guidelines regarding clinical trials both during and after study completion.

The Principal Investigators will be responsible for assuring that all the required data will be collected and entered onto the Case Report Forms. Periodically, monitoring visits will be conducted and the Principal Investigator will provide access to his/her original records to permit verification of proper entry of data. At the completion of the study, all case report forms will be reviewed by the Principal Investigators and will require his/her final signature to verify the accuracy of the data.

8 REFERENCES

1. Westin, J.R. and R. Kurzrock, *It's about time: lessons for solid tumors from chronic myelogenous leukemia therapy.* Mol Cancer Ther, 2012. **11**(12): p. 2549-55.
2. Pindolia, V.K. and B.J. Zarowitz, *Imatinib mesylate, the first molecularly targeted gene suppressor.* Pharmacotherapy, 2002. **22**(10): p. 1249-65.
3. Sicklick, J.K. and N.E. Lopez, *Optimizing Surgical and Imatinib Therapy for the Treatment of Gastrointestinal Stromal Tumors.* J Gastrointest Surg, 2013.
4. Dematteo, R.P., et al., *Adjuvant imatinib mesylate after resection of localised, primary gastrointestinal stromal tumour: a randomised, double-blind, placebo-controlled trial.* Lancet, 2009. **373**(9669): p. 1097-104.
5. Said, R., et al., *P53 Mutations in Advanced Cancers: Clinical Characteristics, Outcomes, and Correlation between Progression-Free Survival and Bevacizumab-Containing Therapy.* Oncotarget, 2013. **4**(5): p. 705-14.
6. Tsimberidou, A.M., et al., *Personalized medicine in a phase I clinical trials program: the MD Anderson Cancer Center initiative.* Clin Cancer Res, 2012. **18**(22): p. 6373-83.
7. Kim, E.S., et al., *The BATTLE trial: personalizing therapy for lung cancer.* Cancer Discov, 2011. **1**(1): p. 44-53.
8. Janku, F., et al., *Assessing PIK3CA and PTEN in early-phase trials with PI3K/AKT/mTOR inhibitors.* Cell Rep, 2014. **6**(2): p. 377-87.
9. Schwaederle, M., et al., *Molecular Tumor Board: The University of California San Diego Moores Cancer Center Experience.* Oncologist, 2014.
10. Von Hoff, D.D., et al., *Pilot study using molecular profiling of patients' tumors to find potential targets and select treatments for their refractory cancers.* J Clin Oncol, 2010. **28**(33): p. 4877-83.
11. Peters, W.P. and M.C. Rogers, *Variation in approval by insurance companies of coverage for autologous bone marrow transplantation for breast cancer.* N Engl J Med, 1994. **330**(7): p. 473-7.
12. Klamerus, J.F., et al., *The impact of insurance on access to cancer clinical trials at a comprehensive cancer center.* Clin Cancer Res, 2010. **16**(24): p. 5997-6003.
13. Heinrich, M.C., et al., *Phase II, open-label study evaluating the activity of imatinib in treating life-threatening malignancies known to be associated with imatinib-sensitive tyrosine kinases.* Clin Cancer Res, 2008. **14**(9): p. 2717-25.

Appendix A. Response Evaluation Criteria in Solid Tumors (RECIST)

Tumor assessments will be made according to the schedule of assessments. Response and progression will be evaluated using Response Evaluation Criteria in Solid Tumors (RECIST) version 1.1 guidelines [Eisenhauer et al. 2009].

1 Measurability of Tumor at Baseline

1.1 <u>Definitions</u>

At baseline, tumor lesions will be categorized as follows:

1.1.1 Measurable

Tumor lesions: must be accurately measured in at least one dimension (longest diameter in the plane of measurements is to be recorded) with a minimum size of:

4. 10 mm by CT scan (CT scan slice thickness no greater than 5 mm).
5. 10 mm caliper measurement by clinical exam (lesions which cannot be accurately measured with calipers should be recorded as non-measureable).
6. 20 mm by chest X-ray.

Malignant lymph nodes: to be considered pathologically enlarged and measurable, a lymph node must be >= 15 mm in *short* axis when assessed by CT scan. At baseline and in follow-up, only the *short* axis will be measured and followed.

1.1.2 Non-measurable

All other lesions, including small lesions (longest diameter < 10 mm or pathological lymph nodes with >= 10 to < 15 mm short axis) as well as truly non-measurable lesions. Lesions considered truly non-measurable include: leptomeningeal disease, ascites, pleural or pericardial effusion, inflammatory breast disease, lymphangitic involvement of skin or lung, abdominal masses/abdominal organomegaly identified by physical exam that is not measurable by reproducible imaging techniques.

1.1.3 Special considerations regarding lesion measurability

Bone lesions, cystic lesions, and lesions previously treated with local therapy require particular comment:

Bone lesions:

- Bone scan, PET scan or plain films are not considered adequate imaging techniques to measure bone lesions. However, these techniques can be used to confirm the presence or disappearance of bone lesions.
- Lytic bone lesions or mixed lytic-blastic lesions, with *identifiable soft tissue components*, that can be evaluated by cross sectional imaging techniques such as CT or MRI can be considered as measurable lesions if the soft tissue component meets the definition of measurability described above.
- Blastic bone lesions are non-measurable.

Cystic lesions:

- Lesions that meet the criteria for radiographically defined simple cysts should not be considered as malignant lesions (neither measurable nor non-measurable) since they are, by definition, simple cysts.
- 'Cystic lesions' thought to represent cystic metastases can be considered as measurable lesions, if they meet the definition of measurability described above. However, if non-cystic lesions are present in the same patient, these are preferred for selection as target lesions.

Lesions with prior local treatment:

- Tumor lesions situated in a previously irradiated area, or in an area subjected to other loco-regional therapy, are usually not considered measurable unless there has been demonstrated progression in the lesion. Study protocols should detail the conditions under which such lesions would be considered measurable.

1.2 Specifications by methods of measurements

1.2.1 Measurement of lesions

All measurements should be recorded in metric notation, using calipers if clinically assessed. All baseline evaluations should be performed as close as possible to the treatment start and never more than 4 weeks before the beginning of the treatment.

1.2.2 Method of assessment

The same method of assessment and the same technique should be used to characterize each identified and reported lesion at baseline and during follow-up. Imaging based evaluation should always be done rather than clinical examination unless the lesion(s) being followed cannot be imaged but are assessable by clinical exam.

Clinical lesions: Clinical lesions will only be considered measurable when they are superficial and P10mm diameter as assessed using calipers (e.g. skin nodules). For the case of skin lesions, documentation by color photography including a ruler to estimate the size of the lesion is suggested. As noted above, when lesions can be evaluated by both clinical exam and imaging, imaging evaluation should be undertaken since it is more objective and may also be reviewed at the end of the study.

Chest X-ray: Chest CT is preferred over chest X-ray, particularly when progression is an important endpoint, since CT is more sensitive than X-ray, particularly in identifying new lesions. However, lesions on chest X-ray may be considered measurable if they are clearly defined and surrounded by aerated lung.

CT, PET-CT, MRI: CT is the best currently available and reproducible method to measure lesions selected for response assessment. This guideline has defined measurability of lesions on CT scan based on the assumption that CT slice thickness is 5mm or less. When CT scans have slice thickness greater than 5 mm, the minimum size for a measurable lesion should be twice the slice thickness. PET-CT is also acceptable in certain situations (e.g. for body scans). MRI is also acceptable in certain situations (e.g. for body scans).

Ultrasound: Ultrasound is not useful in assessment of lesion size and should not be used as a method of measurement. Ultrasound examinations cannot be reproduced in their entirety for independent review at a later date and, because they are operator dependent, it cannot be guaranteed that the same technique and measurements will be taken from one assessment to the next. If new lesions are identified by ultrasound in the course of the study, confirmation by CT or MRI is advised. If there is concern about radiation exposure at CT, MRI may be used instead of CT in selected instances.

Endoscopy, laparoscopy: The utilization of these techniques for objective tumor evaluation is not advised. However, they can be useful to confirm complete pathological response when biopsies are obtained or to determine relapse in trials where recurrence following complete response or surgical resection is an endpoint.

Tumor markers: Tumor markers alone cannot be used to assess objective tumor response. If markers are initially above the upper normal limit, however, they must normalize for a patient to be considered in complete response. Because tumor markers are disease specific, instructions for their measurement should be incorporated into protocols on a disease specific basis.

Cytology, histology: These techniques can be used to differentiate between PR and CR in rare cases if required by protocol (for example, residual lesions in tumor types such as germ cell tumors, where known residual benign tumors can remain). When effusions are known to be a potential adverse effect of treatment (e.g. with certain taxane compounds or angiogenesis inhibitors), the cytological confirmation of the neoplastic origin of any effusion that appears or worsens during treatment can be considered if the measurable tumor has met criteria for response or stable disease in order to differentiate between response (or stable disease) and progressive disease.

2 Tumor Response Evaluation

2.1 <u>Baseline documentation of ‘target’ and ‘non-target’ lesions</u>

When more than one measurable lesion is present at baseline all lesions up to a maximum of five lesions total (and a maximum of two lesions per organ) representative of all involved organs should be identified as target lesions and will be recorded and measured at baseline (this means in instances where patients have only one or two organ sites involved a maximum of two and four lesions respectively will be recorded).

Target lesions should be selected on the basis of their size (lesions with the longest diameter), be representative of all involved organs, but in addition should be those that lend themselves to reproducible repeated measurements. It may be the case that, on occasion, the largest lesion does not lend itself to reproducible measurement in which circumstance the next largest lesion that can be measured reproducibly should be selected.

Lymph nodes merit special mention since they are normal anatomical structures that may be visible by imaging even if not involved by tumor. Pathological nodes that are defined as measurable and may be identified as target lesions must meet the criterion of a short axis of P15mm by CT scan. Only the short axis of these nodes will contribute to the baseline sum. The short axis of the node is the diameter normally used by radiologists to judge if a node is involved by solid tumor. Nodal size is normally reported as two dimensions in the plane in which the image is obtained (for CT scan this is almost always the axial plane; for MRI the plane of acquisition may be axial, sagittal or coronal). The smaller of these measures is the short axis.
For example, an abdominal node which is reported as being 20mm· 30mm has a short axis of 20mm and qualifies as a malignant, measurable node. In this example, 20mm should be recorded as the node measurement. All other pathological nodes (those with short axis P10mm but <15 mm) should be considered non-target lesions. Nodes that have a short axis <10mm are considered non-pathological and should not be recorded or followed.

A *sum of the diameters* (longest for non-nodal lesions, short axis for nodal lesions) for all target lesions will be calculated and reported as the baseline sum diameters. If lymph nodes are to be included in the sum, then as noted above, only the short axis is added into the sum. The baseline sum diameters will be used as reference to further characterize any objective tumor regression in the measurable dimension of the disease.

All other lesions (or sites of disease) including pathological lymph nodes should be identified as non-target lesions and should also be recorded at baseline. Measurements are not required and these lesions should be followed as ‘present’, ‘absent’, or in rare cases ‘unequivocal progression’ (more details to follow). In addition, it is possible to record multiple non-target lesions involving the same organ as a single item on the case record form (e.g. ‘multiple enlarged pelvic lymph nodes’ or ‘multiple liver metastases’).

2.2 <u>Response criteria</u>

This section provides the definitions of the criteria used to determine objective tumor response for target lesions.

2.2.1 Evaluation of target lesions

Complete Response (CR): Disappearance of all target lesions. Any pathological lymph nodes (whether target or non-target) must have reduction in short axis to <10 mm.

Partial Response (PR): At least a 30% decrease in the sum of diameters of target lesions, taking as reference the baseline sum diameters.

Progressive Disease (PD): At least a 20% increase in the sum of diameters of target lesions, taking as reference the smallest sum on study (this includes the baseline sum if that is the smallest on study). In addition to the relative increase of 20%, the sum must also demonstrate an absolute increase of at least 5 mm. (Note: the appearance of one or more new lesions is also considered progression).

Stable Disease (SD): Neither sufficient shrinkage to qualify for PR nor sufficient increase to qualify for PD, taking as reference the smallest sum diameters while on study.

2.2.2 Special notes on the assessment of target lesions

Lymph nodes. Lymph nodes identified as target lesions should always have the actual short axis measurement recorded (measured in the same anatomical plane as the baseline examination), even if the nodes regress to below 10mm on study. This means that when lymph nodes are included as target lesions, the ‘sum’ of lesions may not be zero even if complete response criteria are met, since a normal lymph node is defined as having a short axis of <10mm. Case report forms or other data collection methods may therefore be designed to have target nodal lesions recorded in a separate section where, in order to qualify for CR, each node must achieve a short axis <10mm. For PR, SD and PD, the actual short axis measurement of the nodes is to be included in the sum of target lesions.

Target lesions that become ‘too small to measure’. While on study, all lesions (nodal and non-nodal) recorded at baseline should have their actual measurements recorded at each subsequent evaluation, even when very small (e.g. 2mm). However, sometimes lesions or lymph nodes which are recorded as target lesions at baseline become so faint on CT scan that the radiologist may not feel comfortable assigning an exact measure and may report them as being ‘too small to measure’. When this occurs it is important that a value be recorded on the case report form. If it is the opinion of the radiologist that the lesion has likely disappeared, the measurement should be recorded as 0mm. If the lesion is believed to be present and is faintly seen but too small to measure, a default value of 5mm should be assigned (Note: It is less likely that this rule will be used for lymph nodes since they usually have a definable size when normal and are frequently surrounded by fat such as in the retroperitoneum; however, if a lymph node is believed to be present and is faintly seen but too small to measure, a default value of 5mm should be assigned

in this circumstance as well). This default value is derived from the 5mm CT slice thickness (but should not be changed with varying CT slice thickness). The measurement of these lesions is potentially non-reproducible; therefore providing this default value will prevent false responses or progressions based upon measurement error. To reiterate, however, if the radiologist is able to provide an actual measure, that should be recorded, even if it is below 5mm.

Lesions that split or coalesce on treatment. When non-nodal lesions 'fragment', the longest diameters of the fragmented portions should be added together to calculate the target lesion sum. Similarly, as lesions coalesce, a plane between them may be maintained that would aid in obtaining maximal diameter measurements of each individual lesion. If the lesions have truly coalesced such that they are no longer separable, the vector of the longest diameter in this instance should be the maximal longest diameter for the 'coalesced lesion'.

2.2.3 Evaluation of non-target lesions

This section provides the definitions of the criteria used to determine the tumor response for the group of non-target lesions. While some non-target lesions may actually be measurable, they need not be measured and instead should be assessed only qualitatively at the time points specified in the protocol.

Complete Response (CR): Disappearance of all non-target lesions and normalization of tumor marker level. All lymph nodes must be non-pathological in size (<10mm short axis).

Non-CR/Non-PD: Persistence of one or more non-target lesion(s) and/or maintenance of tumor marker level above the normal limits.

Progressive Disease (PD): Unequivocal progression (see comments below) of existing non-target lesions. (Note: the appearance of one or more new lesions is also considered progression).

2.2.4 Special notes on assessment of progression of non-target disease

The concept of progression of non-target disease requires additional explanation as follows:

When the patient also has measurable disease. In this setting, to achieve 'unequivocal progression' on the basis of the non-target disease, there must be an overall level of substantial worsening in non-target disease such that, even in presence of SD or PR in target disease, the overall tumor burden has increased sufficiently to merit discontinuation of therapy. A modest 'increase' in the size of one or more non-target lesions is usually not sufficient to quality for unequivocal progression status. The designation of overall progression solely on the basis of change in non-target disease in the face of SD or PR of target disease will therefore be extremely rare.

When the patient has only non-measurable disease. This circumstance arises in some phase III trials when it is not a criterion of study entry to have measurable disease. The same general concepts apply here as noted above, however, in this instance there is no measurable disease assessment to factor into the interpretation of an increase in non-measurable disease burden. Because worsening in non-target disease cannot be easily quantified (by definition: if all lesions are truly non-measurable) a useful test that can be applied when assessing patients for unequivocal progression is to consider if the increase in overall disease burden based on the change in non-measurable disease is comparable in magnitude to the increase that would be required to declare PD for measurable disease: i.e.an increase in tumor burden representing an additional 73% increase in 'volume' (which is equivalent to a 20% increase diameter in a measurable lesion). Examples include an increase in a pleural effusion from 'trace' to 'large', an increase in lymphangitic disease from localized to widespread, or may be described in protocols

as ‘sufficient to require a change in therapy’. If ‘unequivocal progression’ is seen, the patient should be considered to have had overall PD at that point. While it would be ideal to have objective criteria to apply to non-measurable disease, the very nature of that disease makes it impossible to do so; therefore the increase must be substantial.

2.2.5 New lesions

The appearance of new malignant lesions denotes disease progression; therefore, some comments on detection of new lesions are important. There are no specific criteria for the identification of new radiographic lesions; however, the finding of a new lesion should be unequivocal: i.e. not attributable to differences in scanning technique, change in imaging modality or findings thought to represent something other than tumor (for example, some ‘new’ bone lesions may be simply healing or flare of pre-existing lesions). This is particularly important when the patient’s baseline lesions show partial or complete response. For example, necrosis of a liver lesion may be reported on a CT scan report as a ‘new’ cystic lesion, which it is not.

A lesion identified on a follow-up study in an anatomical location that was not scanned at baseline is considered a new lesion and will indicate disease progression. An example of this is the patient who has visceral disease at baseline and while on study has a CT or MRI brain ordered which reveals metastases. The patient’s brain metastases are considered to be evidence of PD even if he/she did not have brain imaging at baseline.

If a new lesion is equivocal, for example because of its small size, continued therapy and follow-up evaluation will clarify if it represents truly new disease. If repeat scans confirm there is definitely a new lesion, then progression should be declared using the date of the initial scan.

While FDG-PET response assessments need additional study, it is sometimes reasonable to incorporate the use of FDG-PET scanning to complement CT scanning in assessment of progression (particularly possible ‘new’ disease). New lesions on the basis of FDG-PET imaging can be identified according to the following algorithm:

- Negative FDG-PET at baseline, with a positive FDG-PET at follow-up is a sign of PD based on a new lesion.
- No FDG-PET at baseline and a positive FDG-PET at follow- up:
- If the positive FDG-PET at follow-up corresponds to a new site of disease confirmed by CT, this is PD. If the positive FDG-PET at follow-up is not confirmed as a new site of disease on CT, additional follow-up CT scans are needed to determine if there is truly progression occurring at that site (if so, the date of PD will be the date of the initial abnormal FDG-PET scan). If the positive FDG-PET at follow-up corresponds to a pre-existing site of disease on CT that is not progressing on the basis of the anatomic images, this is not PD.

2.3 Evaluation of best overall response

The best overall response is the best response recorded from the start of the study treatment until the end of treatment taking into account any requirement for confirmation. On occasion a response may not be documented until after the end of therapy so protocols should be clear if post-treatment assessments are to be considered in determination of best overall response. Protocols must specify how any new therapy introduced before progression will affect best response designation. The patient's best overall response assignment will depend on the findings of both target and non-target disease and will also take into consideration the appearance of new lesions. Furthermore, depending on the nature of the study and the protocol requirements, it may also require confirmatory measurement. Specifically, in non-randomized trials where response is the primary endpoint, confirmation of PR or CR is needed to deem either one the 'best overall response'. This is described further below.

2.3.1 Time point response

It is assumed that at each protocol specified time point, a response assessment occurs. Table 1 provides a summary of the overall response status calculation at each time point for patients who have measurable disease at baseline.
When patients have non-measurable (therefore non-target) disease only, Table 2 is to be used.

Appendix Table 1. Time point response: patients with target (+/- non-target) disease.

Target Lesions	Non-target Lesions	New Lesions	Overall Response
CR	CR	No	CR
CR	Non-CR/non-PD	No	PR
CR	Not evaluated	No	PR
PR	Non-PD or not all evaluated	No	PR
SD	Non-PD or not all evaluated	No	SD
Not all evaluated	Non-PD	No	Not evaluable
PD	Any	Yes or No	PD
Any	PD	Yes or No	PD
Any	Any	Yes	PD

Appendix Table 2. Time point response: patients with non-target disease only.

Non-target Lesions	New Lesions	Overall Response
CR	No	CR
Non-CR/non-PD	No	Non-CR/non-PD
Not all evaluated	No	Not evaluable
Unequivocal PD	Yes or No	PD
Any	Yes	PD

2.3.2 Missing assessments and not evaluable designation

When no imaging/measurement is done at all at a particular time point, the patient is not evaluable (NE) at that time point. If only a subset of lesion measurements are made at an assessment, usually the case is also considered NE at that time point, unless a convincing argument can be made that the contribution of the individual missing lesion(s) would not change the assigned time point response. This would be most likely to happen in the case of PD. For example, if a patient had a baseline sum of 50mm with three measured lesions and at follow-up only two lesions were assessed, but those gave a sum of 80 mm, the patient will have achieved PD status, regardless of the contribution of the missing lesion.

2.3.3 Best overall response: all time points

The best overall response is determined once all the data for the patient is known.

Best response determination in trials where confirmation of complete or partial response IS NOT required: Best response in these trials is defined as the best response across all time points (for example, a patient who has SD at first assessment, PR at second assessment, and PD on last assessment has a best overall response of PR). When SD is believed to be best response, it must also meet the protocol specified minimum time from baseline. If the minimum time is not met when SD is otherwise the best time point response, the patient's best response depends on the subsequent assessments. For example, a patient who has SD at first assessment, PD at second and does not meet minimum duration for SD, will have a best response of PD. The same patient lost to follow-up after the first SD assessment would be considered not evaluable.

Best response determination in trials where confirmation of complete or partial response IS required: Complete or partial responses may be claimed only if the criteria for each are met at a subsequent time point as specified in the protocol (generally 4 weeks later). In this circumstance, the best overall response can be interpreted as in Table 3.

Appendix Table 3. Best overall response when confirmation of CR and PR required.

**Overall response First time point**	**Overall response Subsequent time point**	**BEST overall response**
CR	CR	CR
CR	PR	SD, PD OR PR
CR	SD	SD provided minimum criteria for SD duration met, otherwise PD
CR	PD	SD provided minimum criteria for SD duration met, otherwise PD
CR	Not evaluable	SD provided minimum criteria for SD duration met, otherwise not evaluable
PR	CR	PR
PR	PR	PR
PR	SD	SD
PR	PD	SD provided minimum criteria for SD duration met, otherwise PD
PR	Not evaluable	SD provided minimum criteria for SD duration met, otherwise Not evaluable
Not evaluable	Not evaluable	Not evaluable

2.3.4 Special notes on response assessment

When nodal disease is included in the sum of target lesions and the nodes decrease to 'normal' size (<10 mm), they may still have a measurement reported on scans. This measurement should be recorded even though the nodes are normal in order not to overstate progression should it be based on increase in size of the nodes. As noted earlier, this means that patients with CR may not have a total sum of 'zero' on the case report form (CRF).

In trials where confirmation of response is required, repeated 'NE' time point assessments may complicate best response determination. The analysis plan for the trial must address how missing data/assessments will be addressed in determination of response and progression. For example, in most trials it is reasonable to consider a patient with time point responses of PR-NE-PR as a confirmed response.

Patients with a global deterioration of health status requiring discontinuation of treatment without objective evidence of disease progression at that time should be reported as ‘symptomatic deterioration’. Every effort should be made to document objective progression even after discontinuation of treatment. Symptomatic deterioration is not a descriptor of an objective response: it is a reason for stopping study therapy. The objective response status of such patients is to be determined by evaluation of target and non-target disease as shown in Tables 1–3.

Conditions that define ‘early progression, early death and not evaluable’ are study specific and should be clearly described in each protocol (depending on treatment duration, treatment periodicity).

In some circumstances it may be difficult to distinguish residual disease from normal tissue. When the evaluation of complete response depends upon this determination, it is recommended that the residual lesion be investigated (fine needle aspirate/biopsy) before assigning a status of complete response. FDG-PET may be used to upgrade a response to a CR in a manner similar to a biopsy in cases where a residual radiographic abnormality is thought to represent fibrosis or scarring. The use of FDG-PET in this circumstance should be prospectively described in the protocol and supported by disease specific medical literature for the indication. However, it must be acknowledged that both approaches may lead to false positive CR due to limitations of FDG-PET and biopsy resolution/sensitivity.

For equivocal findings of progression (e.g. very small and uncertain new lesions; cystic changes or necrosis in existing lesions), treatment may continue until the next scheduled assessment. If at the next scheduled assessment, progression is confirmed, the date of progression should be the earlier date when progression was suspected.

2.4 Confirmatory measurement/duration of response

2.4.1 Confirmation

In non-randomized trials where response is the primary endpoint, confirmation of PR and CR is required to ensure responses identified are not the result of measurement error. This will also permit appropriate interpretation of results in the context of historical data where response has traditionally required confirmation in such trials. However, in all other circumstances, i.e. in randomized trials (phase II or III) or studies where stable disease or progression are the primary endpoints, confirmation of response is not required since it will not add value to the interpretation of trial results. However, elimination of the requirement for response confirmation may increase the importance of central review to protect against bias, in particular in studies that are not blinded.

In the case of SD, measurements must have met the SD criteria at least once after study entry at a minimum interval (in general not less than 6–8 weeks) that is defined in the study protocol.

2.4.2 Duration of overall response

The duration of overall response is measured from the time measurement criteria are first met for CR/PR (whichever is first recorded) until the first date that recurrent or progressive disease is objectively documented (taking as reference for progressive disease the smallest measurements recorded on study).

The duration of overall complete response is measured from the time measurement criteria are first met for CR until the first date that recurrent disease is objectively documented.

2.4.3 Duration of stable disease

Stable disease is measured from the start of the treatment (in randomized trials, from date of

randomization) until the criteria for progression are met, taking as reference the smallest sum on study (if the baseline sum is the smallest, this is the reference for calculation of PD).

The clinical relevance of the duration of stable disease varies in different studies and diseases. If the proportion of patients achieving stable disease for a minimum period of time is an endpoint of importance in a particular trial, the protocol should specify the minimal time interval required between two measurements for determination of stable disease.

Note: The duration of response and stable disease as well as the progression-free survival are influenced by the frequency of follow-up after baseline evaluation. It is not in the scope of this guideline to define a standard follow-up frequency. The frequency should take into account many parameters including disease types and stages, treatment periodicity and standard practice. However, these limitations of the precision of the measured endpoint should be taken into account if comparisons between trials are to be made.
