# Supplementary material for: Molecular profiling of advanced malignancies guides first-line N-of-1 treatments in the I-PREDICT treatment-naïve study
Source: Genome Med. 2021 Oct 4;13:155. doi: 10.1186/s13073-021-00969-w (PMC8491393; doi:10.1186/s13073-021-00969-w)
Supplement: Supplementary file 3 — Additional file 3: Table S3. Multivariate Analyses of Progression-free Survival, Overall Survival, and Disease Control Rate in All Treatment-naïve Patients (N = 76) and Excluding Patients with TP53 Mutations Matched to VEGF Inhibitor (VEGFi) Therapy (N = 60). Table S4. Variables predicting outcome in I-PREDICT treatment-naïve patients (N = 76) combined with I-PREDICT patients with ≥1 prior line of therapy (N = 83) (Matching Score dichotomized at ≥60% versus < 60%). Table S5. Variables predicting outcomes among I-PREDICT treatment-naïve patients (N = 76) (Matching Score dichotomized at > 50% versus ≤50% as per prior report [5]). Table S6. Variables predicting outcome in I-PREDICT treatment-naïve patients (N = 76) combined with I-PREDICT patients with ≥1 prior line of therapy (N = 83) (Matching Score dichotomized at > 50% versus ≤50% as per prior report [5]). Table S7. Rates of serious adverse events (SAE; grades 3-5 of CTCAE v4.03) according to Matching Score in 76 treated patients. Table S8. Possibly/Probably Related serious adverse events (SAE; grades 3-5 of CTCAE v4.03, N = 45 events) according to Grade, Matching Score, and Relationship to Treatment. Table S9. All serious adverse events (SAE; grades 3-5) according to CTCAE v4.03 System Organ Class for all matched and unmatched patients. Figure S1. Co-drug plot of numbers of drugs per patient, drug dose adjustments per patient, and percent of standard drug doses for each drug per patient, and median drug dose per agent for matched patients according to Matching Score. Figure S2. CONSORT Diagram of I-PREDICT Treatment-Naïve Patients (percent of 145 patients). Figure S3. Percentage of Molecularly Matched Patients (N = 54) with a Gene or Pathway Targeted. Figure S4. Strong Linear Correlation between Matching Score and Outcome. Figure S5. Response to treatment among patients evaluable for SD ≥ 6 months/PR/CR (N = 68 of 76 treated patients) (Matching Score > 50% [N = 27] versus ≤50% [N = 41]). Figure S6. Kaplan-Meier cu [file 13073_2021_969_MOESM3_ESM.docx]

**ADDITIONAL FILE 3**

**Molecular Profiling of Advanced Malignancies Guides First-line N-of-1 Treatments in the I-PREDICT Treatment-Naïve Study**

**Authors & Affiliations:**

Jason K. Sicklick, MD^1,2,†^, Shumei Kato, MD^2,3^, Ryosuke Okamura, MD^2,3^, Hitendra Patel, MD^2,3^, Mina Nikanjam, MD, PhD^2,3^, Paul T. Fanta, MD^2,3^, Michael E. Hahn,MD^4^, Pradip De, PhD,^5^ Casey Williams, PhD^5^, Jessica Guido, BS,^2^ Benjamin M. Solomon, MD^5^, Rana R. McKay, MD^2,3^, Amy Krie, MD^5^, Sarah G. Boles, MD^2,3^, Jeffrey S. Ross, MD^6,7^, J. Jack Lee, PhD^8^, Brian Leyland-Jones, MD, PhD^5^, Scott M. Lippman, MD^2,3*^, and Razelle Kurzrock, MD^2,3^**^†*^**

^1^ Department of Surgery, Division of Surgical Oncology, UC San Diego School of Medicine, San Diego, CA

^2^ Center for Personalized Cancer Therapy, Moores Cancer Center, La Jolla CA

^3^ Department of Medicine, Division of Hematology Oncology, UC San Diego School of Medicine, San Diego, CA

^4^ Department of Radiology, UC San Diego School of Medicine, San Diego, CA

^5^ Avera Cancer Institute, Sioux Falls, SD

^6^ Foundation Medicine, Inc., Cambridge, MA

^7^ Departments of Pathology and Urology, SUNY Upstate Medical University, Syracuse, NY

^8^ Department of Biostatistics, University of Texas MD Anderson Cancer Center, Houston, Texas

† **Co-corresponding Authors:**

| Jason K. Sicklick, MD  Professor of Surgery  Division of Surgical Oncology  UC San Diego Moores Cancer Center  UC San Diego Health Sciences  3855 Health Sciences Drive  Mail Code 0987  La Jolla, CA 92093-0987  Tel: 858-822-3967  Fax: 858-228-5153  Email: [jsicklick@health.ucsd.edu](mailto:jsicklick@health.ucsd.edu) | Razelle Kurzrock, MD  Distinguished Professor of Medicine  Division of Hematology/Oncology  UC San Diego Moores Cancer Center  UC San Diego Health Sciences  3855 Health Sciences Drive  Mail Code 0658  La Jolla, CA 92093-0658  Tel: 858-246-1102  Fax: 858-246-1915  E-mail: [rkurzrock@health.ucsd.edu](mailto:rkurzrock@health.ucsd.edu) |
| --- | --- |

* Contributed equally.

**Table S3. Multivariate Analyses of Progression-free Survival, Overall Survival, and Disease Control Rate in All Treatment-naïve Patients (N = 76) and Excluding Patients with *TP53* Mutations Matched to VEGF Inhibitor (VEGFi) Therapy (N = 60).**

|  | **Median PFS (months)** | | **Median OS (months)** | | **Disease Control Rate (%)** | |
| --- | --- | --- | --- | --- | --- | --- |
|  | **All patients (n=76)** | ***TP53*/VEGFi excluded**  **(n=60)** | **All patients**  **(n=76)** | ***TP53*/VEGFi excluded**  **(n=60)** | **All patients**  **(n=68)** | ***TP53*/VEGFi excluded**  **(n=54*)** |
| **Matching Score** |  |  |  |  |  |  |
| ≥60% | 11.6 | 13.8 | 18.7 | Not reached | 68% | 79% |
| <60% | 2.8 | 2.5 | 11.6 | 13.9 | 30% | 30% |
| **P-value** | **0.008** | **0.003** | 0.053 | **0.02** | **0.005** | **0.004** |
| HR (95% CI) | 0.45 (0.25-0.83) | 0.31 (0.13-0.70) | 0.51 (0.25-1.02) | 0.33 (0.12-0.85) |  |  |

*Note: 5 had ongoing SD for less than 6 months and 1 was not yet staged at data cutoff.

**Table S4. Variables predicting outcome in I-PREDICT treatment-naïve patients (N=76) combined with I-PREDICT patients with ≥1 prior line of therapy (N=83) (Matching Score dichotomized at ≥60% versus <60%).***

|  | ***SD≥6mos/PR/CR (N=137*†*)*** | | | | | ***Progression-free Survival (N=159)*** | | | | | ***Overall Survival (N=159)*** | | | | |
| --- | --- | --- | --- | --- | --- | --- | --- | --- | --- | --- | --- | --- | --- | --- | --- |
| ***Parameters*** | ***Univariate*** | | | ***Multivariate*** | | ***Univariate*** | | | ***Multivariate*** | | ***Univariate*** | | | ***Multivariate*** | |
|  | ***N*** | ***Rate*** | ***P-***  ***value*** | ***OR (95%CI)*** | ***P-***  ***value*** | ***N*** | ***Median***  ***(months)*** | ***P-***  ***value*** | ***HR (95%CI)*** | ***P-***  ***value*** | ***N*** | ***Median***  ***(months)*** | ***P-***  ***value*** | ***HR (95%CI)*** | ***P-***  ***value*** |
| **Age, years**  ≥63  <63 | 69  68 | 39%  35% | 0.72 | -- | -- | 77  82 | 3.9  3.7 | 0.79 | -- | -- | 77  82 | 13.9  17.0 | 0.06 | 1.59 (1.01-2.50) | **0.046** |
| **Gender**  Men  Women | 53  84 | 43%  33% | 0.28 | -- | -- | 64  95 | 4.3  3.7 | 0.21 | -- | -- | 64  95 | 14.5  14.1 | 0.77 | -- | -- |
| **Treatment**  Matched  Unmatched | 108  29 | 43%  17% | **0.02** | 2.85 (0.90-9.01) | 0.08 | 127  32 | 4.2  2.1 | **0.01** | 0.64 (0.39-1.04) | 0.07 | 127  32 | 14.3  15.6 | 0.62 | -- | -- |
| **Matching score**  ≥60%  <60% | 42  95 | 60%  27% | **0.002** | 2.31 (1.01-5.27) | **0.047** | 51  108 | 6.8  3.1 | <**0.001** | 0.56 (0.36-0.88) | **0.01** | 51  108 | 18.7  11.5 | **0.008** | 0.48 (0.28-0.82) | **0.007** |
| **Number of drugs**  ≥2 drugs  Single drug | 107  30 | 38%  33% | 0.67 | -- | -- | 124  35 | 3.7  3.9 | 0.89 | -- | -- | 124  35 | 13.9  20.4 | 0.16 | -- | -- |
| **Disease stage**  Treatment naïve  ≥1 prior line | 68  69 | 44%  30% | 0.11 | 2.04 (0.94-4.41) | 0.07 | 76  83 | 4.3  3.7 | **0.03** | 0.67 (0.45-0.98) | **0.04** | 76  83 | 16.0  11.8 | 0.41 | -- | -- |
| **GI or HPB cancer‡**  Yes  No | 66  71 | 30%  44% | 0.12 | 0.62 (0.29-1.31) | 0.21 | 74  85 | 3.1  4.3 | 0.28 | -- | -- | 74  85 | 13.9  14.7 | 0.42 | -- | -- |
| **Breast cancer**  Yes  No | 13  124 | 54%  36% | 0.23 | -- | -- | 15  144 | 5.6  3.7 | 0.58 | -- | -- | 15  144 | 14.1  14.5 | 0.52 | -- | -- |

* The 83 patients with ≥1 prior line of therapy were from the prior IPREDICT report^1^; variables with *P*<0.15 in the univariate were entered into the multivariate.

† Eight patients in the treatment naïve group and 14 patients in the ≥1 prior line group were not evaluable for the SD≥6months with PR/CR analysis because they had ongoing SD<6 months or were too early; all were evaluable for progression-free survival and overall survival.

‡ Includes colorectal, GI-non-colorectal, appendiceal, hepatobiliary, and pancreatic cancers.

**Abbreviations**: CR, complete response; CUP, carcinoma of unknown primary; GI, gastrointestinal; HPB, hepato-pancreato-biliary; HR, hazard ratio; NR, not reached to median; OR, odds ratio; PR, partial response; SD, stable disease.

**Table S5. Variables predicting outcomes among I-PREDICT treatment-naïve patients (N=76) (Matching Score dichotomized at >50% versus ≤50% as per prior report**(1)**).***

|  | ***SD≥6mos/PR/CR (N=68*†*)*** | | | | | ***Progression-free Survival (N=76)*** | | | | | ***Overall Survival (N=76)*** | | | | |
| --- | --- | --- | --- | --- | --- | --- | --- | --- | --- | --- | --- | --- | --- | --- | --- |
| ***Parameters*** | ***Univariate*** | | | ***Multivariate*** | | ***Univariate*** | | | ***Multivariate*** | | ***Univariate*** | | | ***Multivariate*** | |
|  | ***N*** | ***Rate*** | ***P-***  ***value*** | ***OR (95%CI)*** | ***P-***  ***value*** | ***N*** | ***Median***  ***(months)*** | ***P-***  ***value*** | ***HR (95%CI)*** | ***P-***  ***value*** | ***N*** | ***Median***  ***(months)*** | ***P-***  ***value*** | ***HR (95%CI)*** | ***P-***  ***value*** |
| **Age, years**  ≥63  <63 | 36  32 | 44%  44% | >0.99 | -- | -- | 38  38 | 4.5  4.3 | 0.47 | -- | -- | 38  38 | 15.6  NR | 0.18 | -- | -- |
| **Gender**  Men  Women | 29  39 | 48%  41% | 0.63 | -- | -- | 36  40 | 4.3  4.3 | 0.75 | -- | -- | 36  40 | 17.3  14.7 | 0.50 | -- | -- |
| **Treatment**  Matched  Unmatched | 48  20 | 54%  20% | **0.02** | 2.46 (0.60-10.04) | 0.21 | 54  22 | 5.7  2.1 | **0.007** | 0.62 (0.31-1.22) | 0.16 | 54  22 | 17.7  15.6 | 0.27 | -- | -- |
| **Matching Score**  >50%  ≤50% | 27  41 | 67%  29% | **0.003** | 3.25 (0.99-10.68) | 0.052 | 30  46 | 11.6  2.8 | **0.008** | 0.56 (0.29-1.10) | 0.09 | 30  46 | 18.7  11.6 | 0.08 | 0.45 (0.23-0.88) | **0.02** |
| **Number of drugs**  ≥2 drugs  Single drug | 56  12 | 45%  42% | >0.99 | -- | -- | 62  14 | 4.3  5.7 | 0.88 | -- | -- | 62  14 | 14.3  23.0 | 0.14 | 2.93 (1.02-8.45) | **0.047** |
| **GI or HPB cancer‡**  Yes  No | 34  34 | 38%  50% | 0.46 | -- | -- | 39  37 | 3.1  5.7 | 0.41 | -- | -- | 39  37 | 17.3  15.6 | 0.74 | -- | -- |
| **CUP**  Yes  No | 10  58 | 60%  41% | 0.32 | -- | -- | 10  66 | 4.3  4.3 | 0.74 | -- | -- | 10  66 | 8.3  15.6 | 0.57 | -- | -- |
| **Breast cancer**  Yes  No | 3  65 | 67%  43% | 0.58 | -- | -- | 3  73 | NR  4.3 | 0.20 | -- | -- | 3  73 | 14.7  16.0 | 0.42 | -- | -- |

* Variables with *P*<0.15 in the univariate were entered into the multivariate.

† Eight patients in the treatment naïve group and 14 patients in the ≥1 prior line group were not evaluable for the SD≥6 months/PR/CR analysis because they had ongoing SD<6 months or were too early; all were evaluable for progression-free survival and overall survival.

‡ Includes colorectal, GI-non-colorectal, appendiceal, hepatobiliary, and pancreatic cancers.

**Abbreviations**: CR, complete response; CUP, carcinoma of unknown primary; GI, gastrointestinal; HPB, hepato-pancreato-biliary; HR, hazard ratio; NR, not reached to median; OR, odds ratio; PR, partial response; SD, stable disease.

**Table S6. Variables predicting outcome in I-PREDICT treatment-naïve patients (N=76) combined with I-PREDICT patients with ≥1 prior line of therapy (N=83) (Matching Score dichotomized at >50% versus ≤50% as per prior report**(1)**).***

|  | ***Clinical Benefit [SD≥6mos/PR/CR] (N=137*†*)*** | | | | | ***Progression-free Survival (N=159)*** | | | | | ***Overall Survival (N=159)*** | | | | |
| --- | --- | --- | --- | --- | --- | --- | --- | --- | --- | --- | --- | --- | --- | --- | --- |
| ***Parameters*** | ***Univariate*** | | | ***Multivariate*** | | ***Univariate*** | | | ***Multivariate*** | | ***Univariate*** | | | ***Multivariate*** | |
|  | ***N*** | ***Rate*** | ***P-***  ***value*** | ***OR (95%CI)*** | ***P-***  ***value*** | ***N*** | ***Median***  ***(months)*** | ***P-***  ***value*** | ***HR (95%CI)*** | ***P-***  ***value*** | ***N*** | ***Median***  ***(months)*** | ***P-***  ***value*** | ***HR (95%CI)*** | ***P-***  ***value*** |
| **Age, years**  ≥63  <63 | 69  68 | 39%  35% | 0.72 | -- | -- | 77  82 | 3.9  3.7 | 0.79 | -- | -- | 77  82 | 13.9  17.0 | 0.06 | 1.67 (1.06-2.64) | **0.03** |
| **Gender**  Men  Women | 53  84 | 43%  33% | 0.28 | -- | -- | 64  95 | 4.3  3.7 | 0.21 | -- | -- | 64  95 | 14.5  14.1 | 0.77 | -- | -- |
| **Treatment**  Matched  Unmatched | 108  29 | 43%  17% | **0.02** | 2.31 (0.71-7.45) | 0.16 | 127  32 | 4.2  2.1 | **0.01** | 0.72 (0.44-1.16) | 0.18 | 127  32 | 14.3  15.6 | 0.62 | -- | -- |
| **Matching Score**  >50%  ≤50% | 47  90 | 57%  26% | <**0.001** | 3.20 (1.40-7.32) | **0.006** | 58  101 | 7.3  3.0 | <**0.001** | 0.46 (0.30-0.71) | <**0.001** | 58  101 | 18.7  11.5 | **0.005** | 0.46 (0.27-0.76) | **0.003** |
| **Number of drugs**  ≥2 drugs  Single drug | 107  30 | 38%  33% | 0.67 | -- | -- | 124  35 | 3.7  3.9 | 0.89 | -- | -- | 124  35 | 13.9  20.4 | 0.16 | -- | -- |
| **Disease stage**  Treatment naïve  ≥1 prior line | 68  69 | 44%  30% | 0.11 | 1.96 (0.90-4.29) | 0.09 | 76  83 | 4.3  3.7 | **0.03** | 0.66 (0.45-0.96) | **0.03** | 76  83 | 16.0  11.8 | 0.41 | -- | -- |
| **GI or HPB cancer‡**  Yes  No | 66  71 | 30%  44% | 0.12 | 0.59 (0.27-1.27) | 0.18 | 74  85 | 3.1  4.3 | 0.28 | -- | -- | 74  85 | 13.9  14.7 | 0.42 | -- | -- |
| **Breast cancer**  Yes  No | 13  124 | 54%  36% | 0.23 | -- | -- | 15  144 | 5.6  3.7 | 0.58 | -- | -- | 15  144 | 14.1  14.5 | 0.52 | -- | -- |

* The 83 patients with ≥1 prior line of therapy were from the prior I-PREDICT report*;*(1) variables with *P*<0.15 in the univariate were entered into the multivariate.

† Eight patients in the treatment naïve group and 14 patients in the ≥1 prior line group were not evaluable for the SD≥6 months/PR/CR analysis because they had ongoing SD<6 months or were too early; all were evaluable for progression-free survival and overall survival.

‡ Includes colorectal, GI-non-colorectal, appendiceal, hepatobiliary, and pancreatic cancers.

**Abbreviations**: CR, complete response; CUP, carcinoma of unknown primary; GI, gastrointestinal; HPB, hepato-pancreato-biliary; HR, hazard ratio; NR, not reached to median; OR, odds ratio; PR, partial response; SD, stable disease.

**Table S7. Rates of serious adverse events (SAE; grades 3-5 of CTCAE v4.03) according to Matching Score in 76 treated patients.**

| **Rate of adverse events, N (%)** | **All treated**  **Patients (N=76)** | **Matching Score** | | | ***P*-values** | | |
| --- | --- | --- | --- | --- | --- | --- | --- |
|  |  | **≥60% (N=27)** | **1%-59% (N=27)** | **Unmatched**  **0% (N=22)** | **≥60% vs.**  **1%-59%** | **≥60% vs.**  **Unmatched** | **1%-59% vs.**  **Unmatched** |
| Any SAE reported (Grade 3–5)  Grade 4–5  Grade 5 (death) | 40 (52.6%)  9 (11.8%)  1 (1.3%) | 17 (63.0%)  4 (14.8%)  0 (0.0%) | 13 (48.1%)  3 (11.1%)  1 (3.7%) | 10 (45.4%)  2 (9.1%)  0 (0.0%) | 0.28  0.69  0.32 | 0.22  0.55  - | 0.85  0.82  0.37 |
| Any SAE related to treatment (≥ Grade 3)*****  Grade 4–5  Grade 5 (death) | 18 (23.6%)  4 (5.2%)  0 (0.0%) | 7 (25.9%)  2 (7.4%)  0 (0.0%) | 6 (22.2%)  0 (0.0%)  0 (0.0%) | 5 (22.7%)  2 (9.1%)  0 (%) | 0.75  0.15  - | 0.80  0.83  - | 0.97  0.11  - |

***** Events considered possibly or probably related to the regimens on the I-PREDICT study.

**Table S8. Possibly/Probably Related serious adverse events (SAE; grades 3-5 of CTCAE v4.03, N=45 events) according to Grade, Matching Score, and Relationship to Treatment.**

Matching Score: green indicates Matching Score ≥60%; yellow indicates Matching Score 1-59%; and red indicates Matching Score 0%. Shades of white to grey distinguish the relationship of SAE to treatment.

| **Study ID** | **SAE terms (CTCAE 4.03)** | **Grade** | **Received ≥1** | **Relationship to Treatment** |
| --- | --- | --- | --- | --- |
| 001 | Cholangitis / bile duct stenosis | 3 | ≥60% | Possible |
| 001 | Vomiting | 3 | ≥60% | Possible |
| 001 | Nausea | 3 | ≥60% | Possible |
| 001 | Sepsis | 4 | ≥60% | Possible |
| 076 | Rash maculo-papular | 3 | ≥60% | Possible |
| 076 | Aspartate aminotransferase increased | 3 | ≥60% | Possible |
| 076 | Alanine aminotransferase increased | 3 | ≥60% | Possible |
| 160 | Urinary tract obstruction/acute renal failure | 3 | ≥60% | Possible |
| 166 | Diarrhea | 3 | ≥60% | Possible |
| 166 | Hypokalemia | 3 | ≥60% | Possible |
| 166 | Severe malnutrition | 3 | ≥60% | Possible |
| 166 | Nausea | 3 | ≥60% | Possible |
| 166 | Pneumatosis intestinalis | 3 | ≥60% | Possible |
| 166 | Nausea and vomiting | 3 | ≥60% | Possible |
| 166 | Elevated lipase | 3 | ≥60% | Possible |
| 166 | Severe malnutrition | 3 | ≥60% | Possible |
| 248 | Elevated TSH | 3 | ≥60% | Possible |
| 248 | Hypothyroidism | 3 | ≥60% | Possible |
| 248 | Hypertension | 3 | ≥60% | Possible |
| A005 | Hypertension | 3 | ≥60% | Possible |
| A005 | Mucositis oral | 3 | ≥60% | Probable |
| A019 | Hyponatremia | 3 | ≥60% | Possible |
| A019 | Hypoxia | 3 | ≥60% | Possible |
| A019 | Duodenal ulcer | 3 | ≥60% | Possible |
| A019 | Lymphocyte count decreased | 4 | ≥60% | Possible |
| A019 | Duodenal perforation | 4 | ≥60% | Probable |
| A019 | Sepsis | 4 | ≥60% | Possible |
| A039 | Muscle weakness upper limb | 3 | 1-59% | Probable |
| A039 | Peripheral sensory neuropathy | 3 | 1-59% | Probable |
| A039 | Arm pain | 3 | 1-59% | Probable |
| 012 | Alanine aminotransferase increased | 3 | 1-59% | Probable |
| 012 | Aspartate aminotransferase increased | 3 | 1-59% | Probable |
| 040 | Atrial Flutter | 3 | 1-59% | Possible |
| 040 | Weakness | 3 | 1-59% | Possible |
| 049 | Neutrophil count decreased | 3 | 1-59% | Possible |
| 087 | Rash | 3 | 1-59% | Possible |
| 087 | Mucositis | 3 | 1-59% | Possible |
| 375 | Pancytopenia | 3 | 1-59% | Possible |
| A006 | Infusion related reaction | 4 | 0% | Probable |
| 106 | Electrocardiogram QT corrected interval prolonged | 4 | 0% | Possible |
| 126 | Diarrhea | 3 | 0% | Possible |
| 209 | Hypertension | 3 | 0% | Probable |
| 344 | Diarrhea | 3 | 0% | Probable |
| 344 | Mucositis | 3 | 0% | Probable |
| 344 | Fatigue | 3 | 0% | Probable |

**Table S9. All serious adverse events (SAE; grades 3-5) according to CTCAE v4.03 System Organ Class for all matched and unmatched patients.**

|  |  |  | **Number of patients (%)** | |
| --- | --- | --- | --- | --- |
| **Body system** | **CTCAE terms** | **Grade** | **All SAEs (N=100, n=%)** | **SAEs related to treatment (n=47, %)*** |
| Blood and lymphatic system disorders | Anemia | 3 | 2% | 0 |
| Cardiac disorders | Atrial flutter | 3 | 1% | 1 (2.1%) |
|  | Chest pain | 3 | 1% | 0 |
|  | Electrocardiogram QT interval prolonged | 4 | 1% | 1 (2.1%) |
|  | Cardiac arrest | 4 | 1% | 0 |
| Ear and labyrinth disorders | Ear pain | 3 | 1% | 0 |
| Gastrointestinal disorders | Abdominal pain | 3 | 3% | 0 |
|  | Vomiting | 3 | 1% | 1 (2.1%) |
|  | Gastric outlet obstruction | 3 | 1% | 0 |
|  | Epigastric abdominal Pain | 3 | 2% | 0 |
|  | Duodenal ulcer | 3 | 1% | 1 (2.1%) |
|  | Diarrhea | 3 | 4% | 3 (6.4%) |
|  | Duodenal perforation | 4 | 1% | 1 (2.1%) |
|  | Dysphagia | 3 | 2% | 1 (2.1%) |
|  | Enterocolitis | 3 | 2% | 0 |
|  | Mucositis oral | 6 | 4% | 3 (6.4%) |
|  | Nausea | 3 | 3% | 3 (6.4%) |
|  | Small intestinal obstruction | 3 | 3% | 0 |
|  | Pneumatosis intestinalis | 3 | 1% | 1 (2.1%) |
|  | Upper gastrointestinal hemorrhage | 3 | 2% | 0 |
| General disorders and administration site conditions | Edema limbs | 3 | 2% | 1 (2.1%) |
|  | Fever | 3 | 2% | 0 |
|  | Dizziness | 3 | 1% | 0 |
|  | Fatigue | 3 | 1% | 1 (2.1%) |
|  | Infusion related reaction | 4 | 1% | 1 (2.1%) |
| Hepatobiliary disorders | Bile duct stenosis | 3 | 4% | 1 (2.1%) |
|  | Biliary obstruction | 3 | 1% | 0 |
|  | Hyperbilirubinemia | 3 | 1% | 0 |
|  |  | 4 | 1% | 0 |
| Infections and infestations | Sepsis | 4 | 4% | 2 (4.3%) |
|  | Pneumonia | 3 | 1% | 0 |
|  | Urinary tract infection | 3 | 1% | 0 |
|  | Infection of the venous access port | 3 | 1% | 0 |
|  | Rash | 3 | 2% | 2 (4.3%) |
| Investigations | Alanine/aspartate aminotransferase increased | 3 | 4% | 4 (8.5%) |
|  | Lymphocyte count decreased | 3 | 1% | 1 (2.1%) |
|  | Elevated lipase | 3 | 1% | 1 (2.1%) |
|  | Neutrophil count decreased | 3 | 1% | 1 (2.1%) |
|  | Pancytopenia | 3 | 1% | 1 (2.1%) |
| Metabolism and nutrition disorders | Anorexia / severe malnutrition | 3 | 2% | 2 (4.3%) |
|  |  | 4 | 1% | 0 |
|  | Hyponatremia | 3 | 1% | 1 (2.1%) |
|  | Hypokalemia | 3 | 1% | 1 (2.1%) |
|  | Hypothyroidism | 3 | 1% | 1 (2.1%) |
|  | Elevated TSH | 3 | 1% | 1 (2.1%) |
| Musculoskeletal and connective tissue disorders | Generalized muscle weakness | 3 | 3% | 1 (2.1%) |
|  | Back pain | 3 | 2% | 0 |
|  | Arm pain | 3 | 1% | 1 (2.1%) |
|  | Muscle weakness upper limb | 3 | 1% | 1 (2.1%) |
| Nervous system disorders | Stroke | 3 | 1% | 0 |
|  | Peripheral sensory neuropathy | 3 | 1% | 1 (2.1%) |
| Renal and urinary disorders | Urinary tract obstruction | 3 | 2% | 1 (2.1%) |
|  | Acute renal injury | 3 | 1% | 0 |
|  | Hematuria | 3 | 1% | 0 |
|  | Obstructive nephropathy | 3 | 1% | 0 |
| Respiratory, thoracic and mediastinal disorders | Hypoxia | 3 | 1% | 1 (2.1%) |
|  | Pleural effusion | 3 | 1% | 0 |
|  | Dyspnea | 6 | 3% | 0 |
|  | Pleural effusion | 5 | 1% | 0 |
|  | Pneumothorax | 3 | 1% | 0 |
|  | Respiratory failure | 4 | 1% | 0 |
| Vascular disorders | Hypertension | 3 | 3% | 3 (6.4%) |

* Events considered possibly or probably related to the regimens on the I-PREDICT study.

**
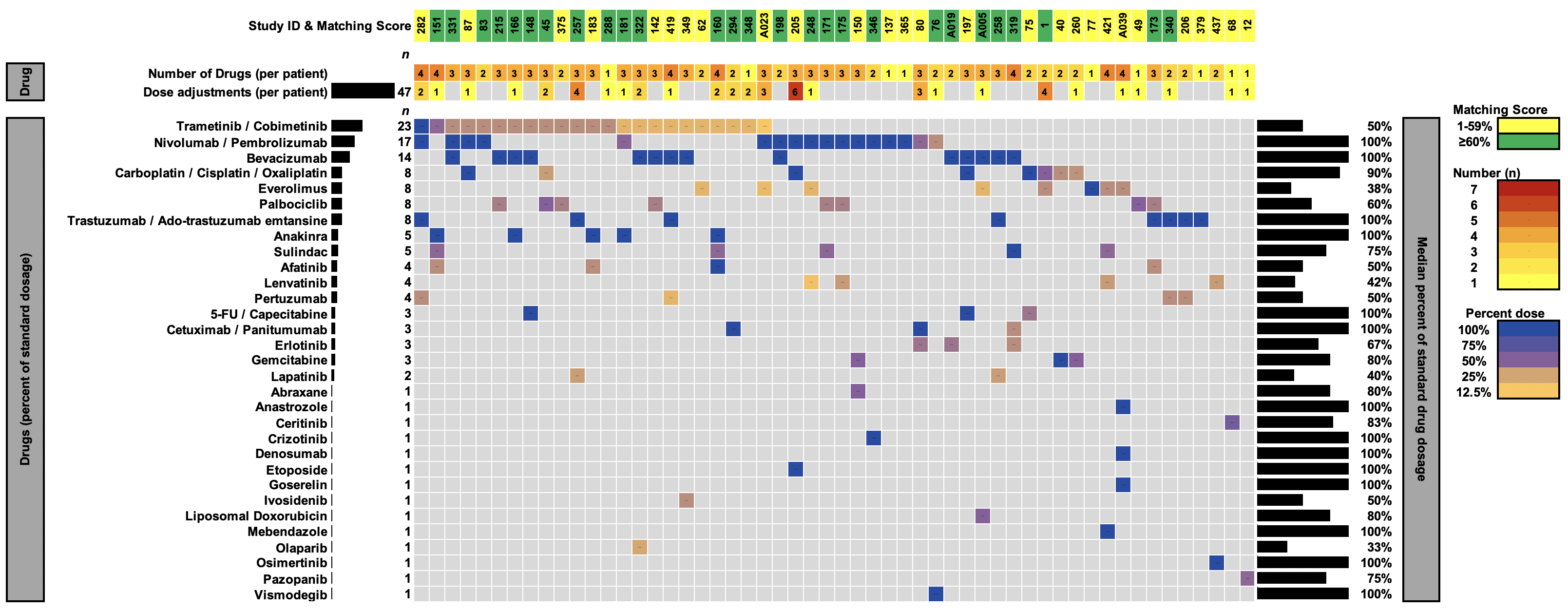
**

**Figure S1**. Co-drug plot of numbers of drugs per patient, drug dose adjustments per patient, and percent of standard drug doses for each drug per patient, and median drug dose per agent for matched patients according to Matching Score.

**Note:** Among the 76 matched patients, there were 47 intra-patient dose adjustments (median 1 per patient, range: 0-6). For each patient, we also include the last drug dose (as a percentage of the standard drug dose for each agent). We also include the median percent of standard dose for each drug in order for the community to obtain maximal information from each patient and the entire cohort. For example, the median dose of MEK inhibitors (i.e., trametinib and cobmetinib) was 50% of the standard dose, while the median dose of immune checkpoint inhibitors (i.e., nivolumab and pembrolizumab) was 100% of the standard dose. For *de novo* combinations, if the patient was responding to reduced doses of drugs and the side effect profile was reasonable, the patients were maintained on the reduced doses. Finally, consistent with the notion that no two patients had the exact same genomic profile, no two patients received the same drug combination. Therefore, there is no toxicity correlation with any specific combination therapy.

**
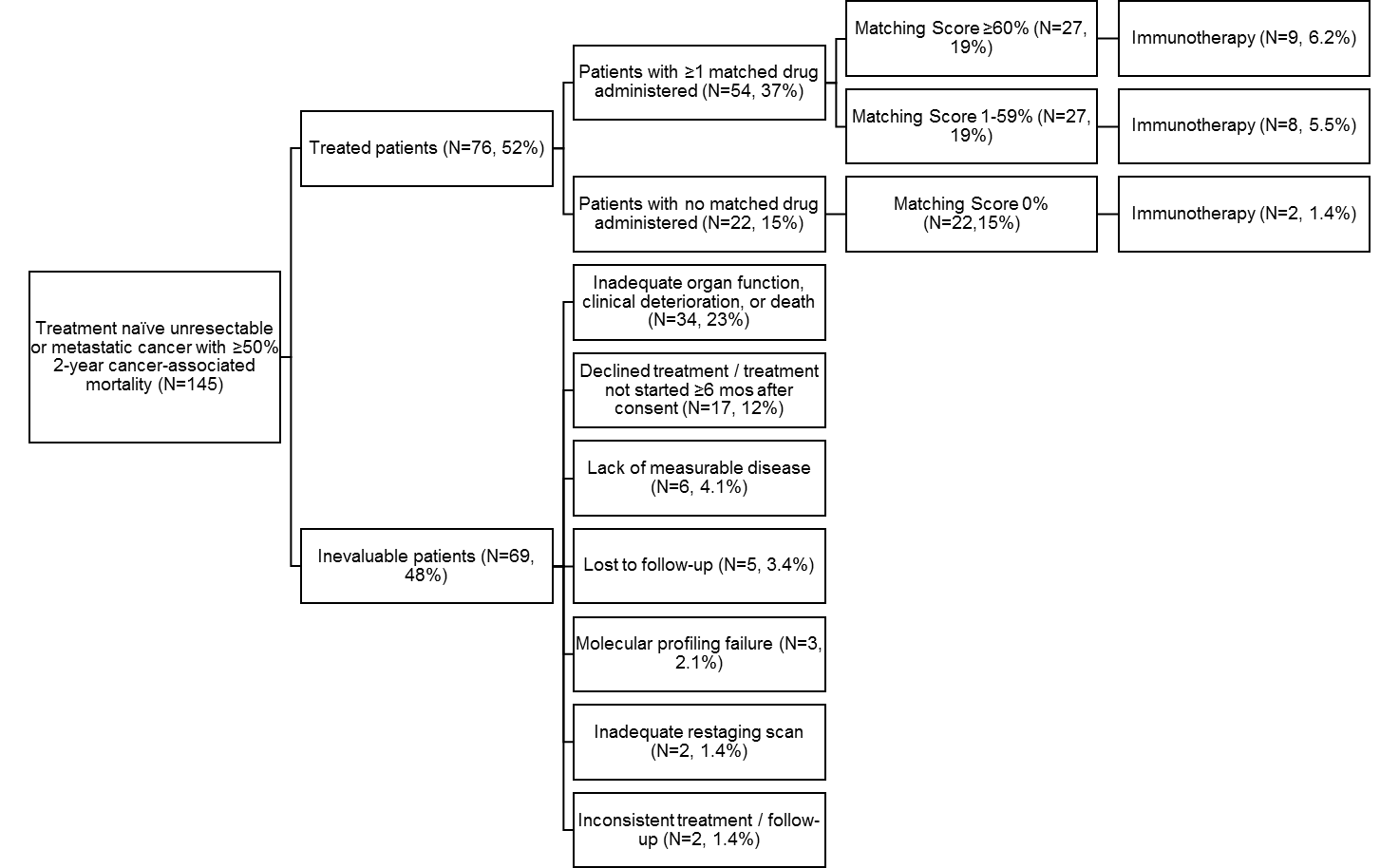
**

**Figure S2. CONSORT Diagram of I-PREDICT Treatment-Naïve Patients (percent of 145 patients).**

**Figure S3. Percentage of Molecularly Matched Patients (N = 54) with a Gene or Pathway Targeted**

**Note:**

- MAPK pathway includes alterations in *BRAF, GNAS, KRAS, NF1, NRAS*, and *RAF1* genes.
- Immune checkpoint inhibitors were matched to the following: mismatch repair *gene alterations*, PD-L1-positive IHC, *PD-L1* amplification; high/intermediate tumor mutational burden (TMB), and high microsatellite instability (MSI-H).
- Cell cycle regulation includes alterations in *CCND1/2, CDK6*, and *CDKN2A*/B genes.
- *ERBB* includes amplification and mutations in *ERBB2* and *ERBB3*.
- PI3K pathway includes alterations in *AKT1, AKT2, PIK3CA, PIK3R1*, and *PTEN*.
- *BRCA*-associated includes *ATM, BAP1, BRCA2*, and *RAD50*.
- Wnt pathway includes alterations in *APC*, *CTNNB1*, and *RNF43* genes.
- Others include *ARID1A, FGF, IDH1, IGF1R, MET, PDGFRA, PTCH1, RET*, and *SMAD4* genes and ER positive (Immunohistochemistry).


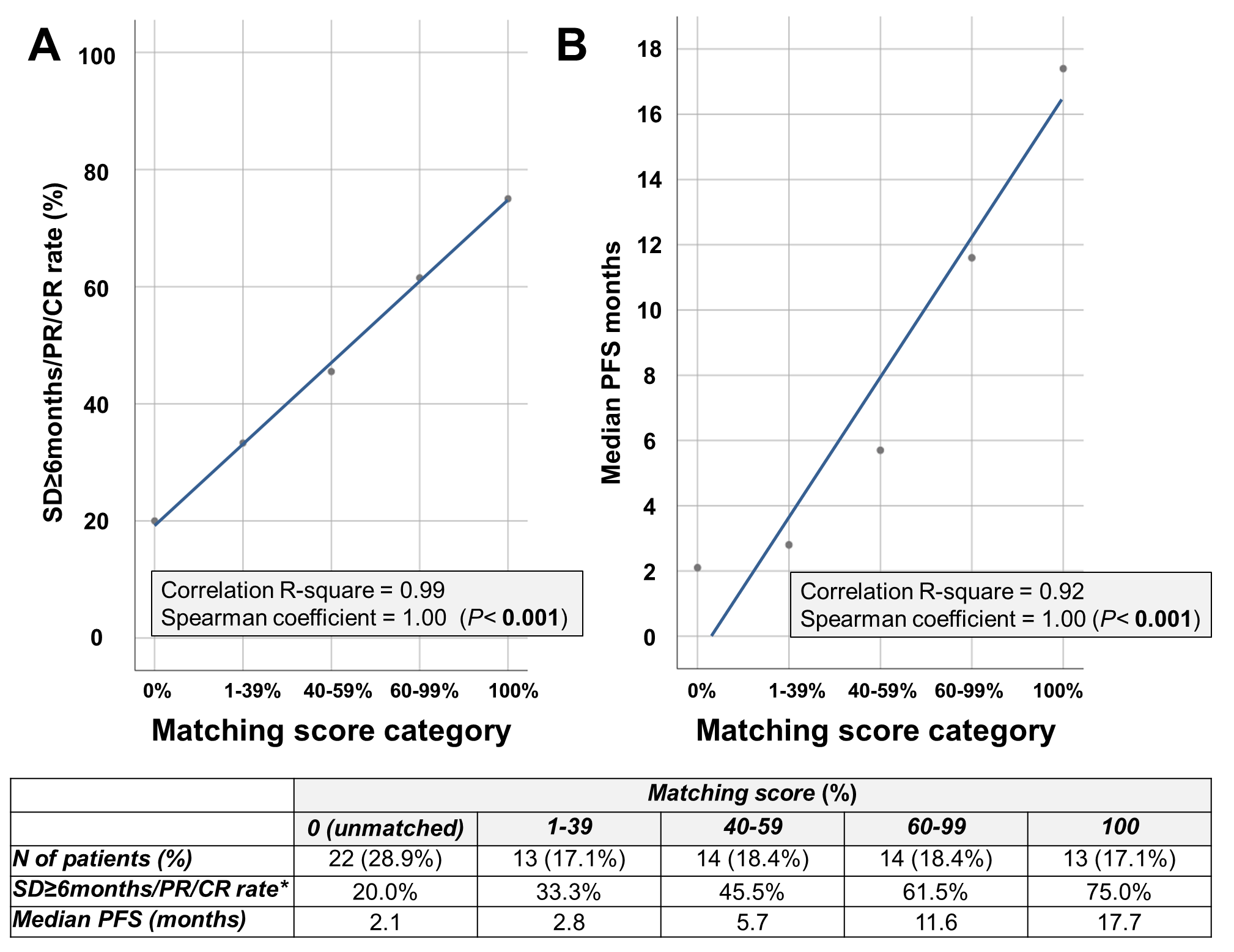


**Figure S4. Strong Linear Correlation between Matching Score and Outcome**

**A**. There was a strong correlation between DCR (SD≥6 months/PR/CR rate) and Matching Score for 68 evaluable patients (8 patients inevaluable because they had ongoing SD<6 months or were too early for evaluation).

**B**. There was a strong correlation between median PFS time (calculated by method of Kaplan-Meier) and Matching Score for 76 evaluable patients.

Note: While OS was independently associated with Matching Score dichotomized at either 60% (MS≥60% vs <60%) or 50% (MS>50% versus ≤50%) (**Table 2, Supplementary Tables 1-3** and **Supplementary Figures 4-6**) the strong linear association between DCR or PFS and Matching Score seen here was not maintained for OS (data not shown). Closer inspection revealed that the OS of unmatched patients might have been confounded by subsequent therapy; indeed, while the median PFS of unmatched patients was only 2.1 months, their median OS was 15.6 months, perhaps because 5 of 22 initially unmatched patients (23%) subsequently received matched therapy, resulting in a second longer PFS. This may have occurred because some physicians, who initially chose not to match patients, reconsidered a matched therapy once the tumor progressed on conventional regimens.)

**Abbreviations**: CR, complete response; DCR, disease control rate; MS, Matching Score; PFS, progression-free survival; PR, partial response; SD, stable disease.


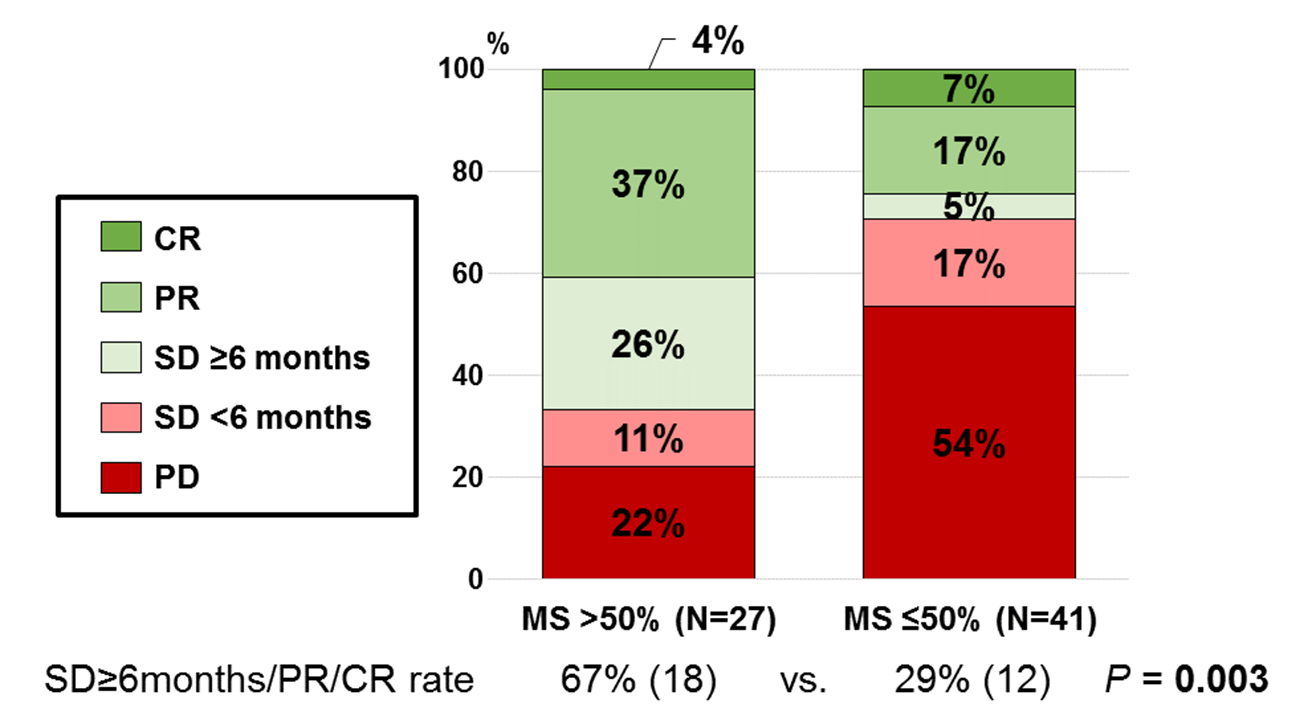


**Figure S5**. Response to treatment among patients evaluable for SD≥6 months/PR/CR (N=68 of 76 treated patients) (Matching Score >50% [N=27] versus ≤50% [N=41]). The following 8 patients were excluded from this analysis: 5 had on-going SD<6 months and 3 were not yet restaged at data cutoff. The 50% as the cutoff was used in first I-PREDICT paper.(1) Results show that dichotomized matching scores correlate with SD≥6 months/PR/CR rate.

**Abbreviations**: CR, complete response; MS, Matching Score; PD, progressive disease; PR, partial response;

SD, stable disease.


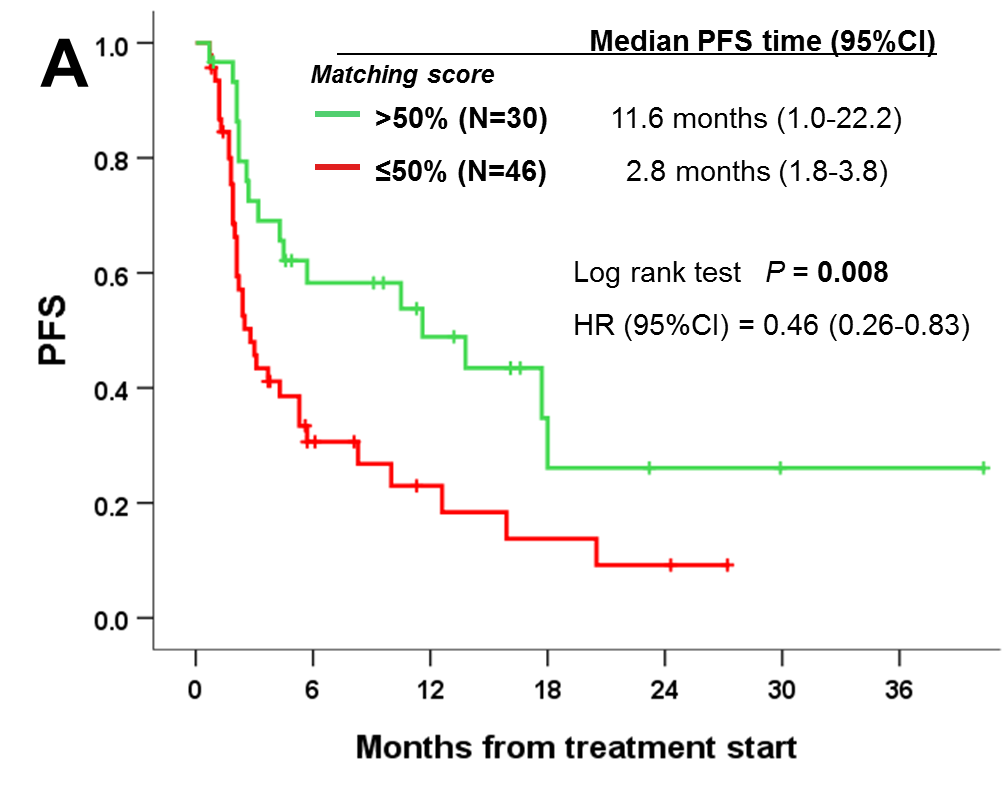


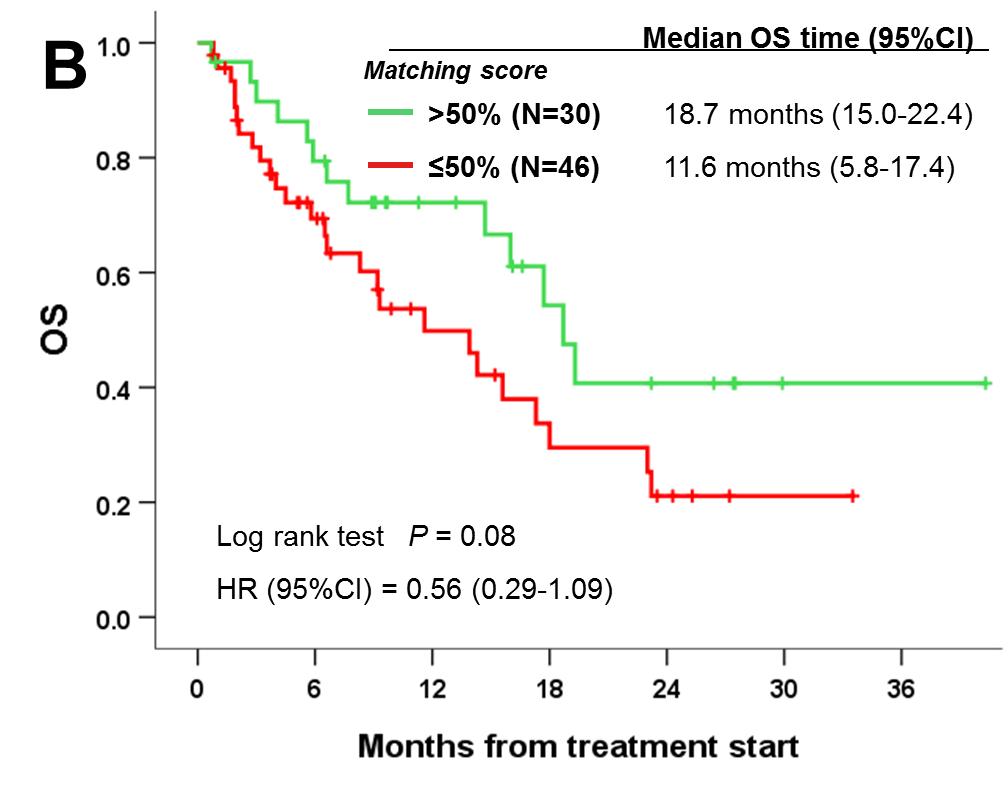


**Figure S6.** Kaplan-Meier curves for (**A**) progression-free survival and (**B**) overall survival according to Matching Score of >50% [N=30] versus ≤50% [N=46]. The 50% as the cutoff was used in first I-PREDICT paper.(1) Results show that dichotomized Matching Scores correlate with outcome.

**Abbreviations**: CI, confidence interval; HR, hazard ratio; OS, overall survival; PFS, progression-free survival.


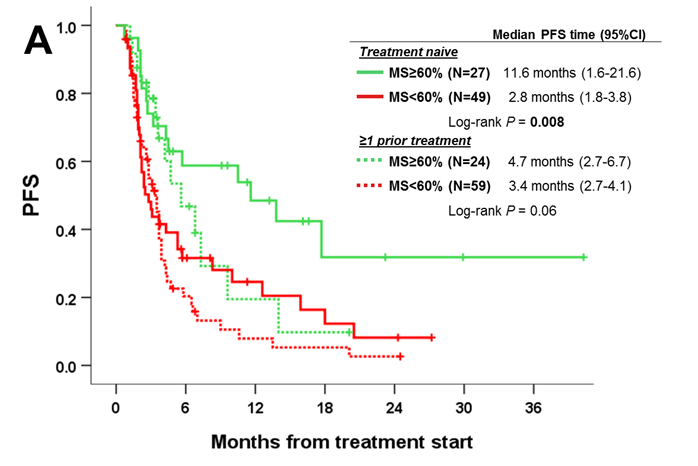


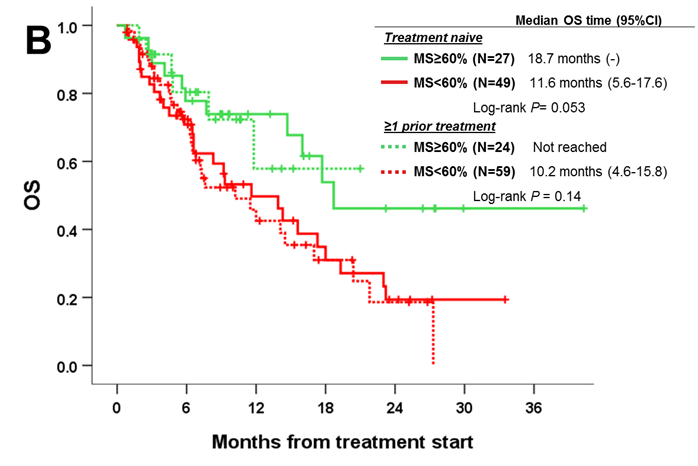


**Figure S7**. Kaplan-Meier curves for (**A**) progression-free survival and (**B**) overall survival according to Matching Score among I-PREDICT treatment-naïve patients (N=76) combined with I-PREDICT patients with ≥1 prior line of therapy (N=83).(1) Results show that dichotomized Matching Scores correlate with outcome.

**Abbreviations**: CI, confidence interval; HR, hazard ratio; MS, Matching Score; OS, overall survival; PFS, progression-free survival.

**REFERENCE FOR ADDITIONAL FILES**

1. Sicklick JK, Kato S, Okamura R, Schwaederle M, Hahn ME, Williams CB*, et al.* Molecular profiling of cancer patients enables personalized combination therapy: the I-PREDICT study. Nat Med **2019**;25(5):744-50 doi 10.1038/s41591-019-0407-5.
